# Mitochondrial CISD1 Modulates Microglial Metabolic Reprogramming to Drive Stress Susceptibility in Mice

**DOI:** 10.1002/advs.202508957

**Published:** 2025-11-11

**Authors:** Wanting Dong, Duo Liu, Songsen Fu, Jiaming Zhang, Xi Chen, Songqiang Huang

**Affiliations:** ^1^ Affiliated Hospital of Hunan University School of Biomedical Sciences Hunan University Changsha 410082 China; ^2^ Clinical Innovation & Research Center (CIRC) Shenzhen Hospital Southern Medical University Shenzhen 518100 China; ^3^ Department of Pharmacy Traditional Chinese and Western Medicine Hospital of Wuhan Tongji Medical College Huazhong University of Science and Technology Wuhan 430033 China

**Keywords:** CDGSH iron sulfur domain 1, depression, medial prefrontal cortex, microglial inflammatory activation, pioglitazone

## Abstract

Depression is one of the most prevalent neuropsychiatric disorders worldwide, and multiple studies have implicated metabolic dysfunction in its pathophysiology. However, the molecular mechanisms by which metabolic pathways modulate depressive‑like behavior remain largely uncharacterized. Here, this work finds that the CDGSH iron sulfur domain 1 (CISD1), a redox protein localized to the outer mitochondrial membrane, is upregulated in the medial prefrontal cortex after chronic stress. Pharmacological inhibition and genetic knockdown of CISD1 significantly ameliorate depressive‐like behavior in mice, and CISD1 knockdown also reverse microglial inflammatory activation. Moreover, this work finds that chronic stress specifically upregulates microglial CISD1 expression, and that conditional knockout of microglial CISD1 alleviates neuroinflammation and depressive‑like behavior in mice. Mechanistically, chronic stress promotes NADH oxidation to generate NAD⁺ by upregulating CISD1 expression. The elevated NAD⁺ functions as a cofactor for glyceraldehyde‐3‐phosphate dehydrogenase, accelerating glycolysis and promoting inflammatory activation. Pioglitazone exerts antidepressant effects by inhibiting NADH oxidation through a CISD1‐dependent pathway in microglia. In conclusion, this study elucidates the role of CISD1 in microglial metabolism, establishing a robust experimental foundation for screening potential antidepressant drugs.

## Graphical abstract

CDGSH iron sulfur domain 1 (CISD1) mitigates oxidative stress by promoting NADH oxidation and Coenzyme Q (CoQ) reduction. Under chronic stress, elevated CISD1 expression in microglia enhances NAD⁺ production, thereby increasing GAPDH activity and glycolytic flux, while reducing ATP synthesis by inhibiting proton transfer from mitochondrial complexes I and II to CoQ. These metabolic alterations collectively drive microglial inflammatory activation.

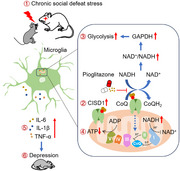

## 1 Introduction

Major depression (MDD) is among the most prevalent and debilitating personal and public health conditions worldwide,^[^ ^1^, ^2^, ^3^ ^]^ affecting ≈280 million people.^[^ ^4^ ^]^ In addition to patients with medication‐resistant depression, Positron Emission Computed Tomography (PET) scans have identified marked reductions in glucose metabolism in the medial prefrontal cortex (mPFC) of MDD.^[^ ^5^ ^]^ Additionally, treatment with antidepressants correlates with significantly enhanced glucose metabolism in the mPFC.^[^ ^6^ ^]^ These studies have shown that cellular metabolism is closely related to the occurrence and development of depression.

Clinical investigations have reported increased lactate levels in both the cortex and cerebrospinal fluid of patients with depression.^[^ ^7^, ^8^ ^]^ Animal studies have shown that in mice subjected to a social defeat stress model, increased lactate levels in the prefrontal cortex are associated with reduced social behaviors, and consistent with this, microglial lactate concentrations are markedly elevated in the brains of mice with depression and Alzheimer's disease.^[^ ^9^, ^10^ ^]^ Collectively, these findings indicate that disturbances in lactate metabolism may contribute to the development of depression. Lactate, a crucial metabolic intermediate produced from pyruvate via glycolysis in astrocytes, plays a vital role in sustaining neuronal activity and modulating depression.^[^ ^11^ ^]^ Moreover, lactate serves not only as a preferred metabolic fuel for immune cells such as microglia, but also as a signaling molecule that regulates their activity.^[^ ^12^, ^13^ ^]^ Nevertheless, there is still a lack of systematic studies to confirm whether stress promotes microglial inflammatory activation by affecting its metabolic phenotype.

Lactate is one of the major end products of glycolysis.^[^ ^14^ ^]^ The cytosolic oxidized nicotinamide adenine dinucleotide (NAD⁺)/NADH redox balance regulates glycolytic flux through the modulation of glyceraldehyde‐3‐phosphate dehydrogenase (GAPDH) activity.^[^ ^15^ ^]^ The GAPDH‐catalyzed step in glycolysis utilizes cytosolic NAD⁺ as a substrate to generate NADH.^[^ ^16^ ^]^ Consequently, the availability of cytoplasmic NAD⁺ can modulate glycolytic flux through GAPDH activity. The mitochondrial protein CDGSH iron sulfur domain 1 (CISD1, also known as mitoNEET), as a redox protein, has been shown to promote the oxidation of NADH in the cytoplasm.^[^ ^17^ ^]^ CISD1 forms a homodimer in the mitochondrial outer membrane.^[^ ^18^ ^]^ It was found that the expression of CISD1 was increased in breast cancer cells, and knockdown of CISD1 resulted in increased glycolysis coupled with decreased oxidative phosphorylation.^[^ ^19^ ^]^ This suggests that CISD1 is involved in the regulation of metabolic balance between oxidative phosphorylation and glycolysis. Previous study has shown that pharmacological inhibition of CISD1 reduces cisplatin‐induced apoptosis and the production of mitochondrial reactive oxygen species (ROS) in cochlear hair cells.^[^ ^20^ ^]^ In addition, accumulation of CISD1 in neurons is toxic, leading to mitochondrial dysfunction and impaired mitophagy.^[^ ^21^ ^]^ However, it remains unclear whether CISD1 contributes to depressive‐like behavior and modulates the microglial metabolic phenotype by altering the NAD⁺/NADH ratio.

In the present study, we found that chronic social defeat stress (CSDS) increased the expression of CISD1 in microglia of the mPFC. Moreover, CISD1 knockout in microglia ameliorated depressive‐like behavior in mice, concomitantly enhancing oxidative phosphorylation and reducing glycolytic activity in microglia. Pioglitazone, a hypoglycemic drug that binds to CISD1 in vivo, ameliorates microglial inflammatory activation and depressive‐like behavior in mice. Our findings reveal an important role for CISD1, a key metabolic regulator, in the development of depression.

## 2 Results

### 2.1 Chronic Stress Increases CISD1 Expression in the mPFC of Mice

CSDS is a classic method for constructing depressive‐like model mice.^[^ ^22^ ^]^ Based on the core characteristics of depression (anhedonia and social avoidance), mice treated with CSDS were categorized into two groups: susceptible and resilient mice.^[^ ^23^, ^24^, ^25^ ^]^ Compared with the control group, susceptible mice showed a significantly reduced social interaction ratio and time in the interaction zone, along with increased time in the corner zone, whereas resilient mice showed no significant change (Figure S1A–D, Supporting Information). The sucrose preference test (SPT) revealed that, compared with the control group, susceptible mice exhibited a reduced sucrose preference percentage, whereas resilient mice showed no significant change (Figure S1E, Supporting Information). In addition, the forced swim test (FST) and tail suspension test (TST) showed that compared with the control group, susceptible mice exhibited significantly longer immobility time, whereas no significant changes were observed in resilient mice (Figure S1F,G, Supporting Information). These results suggest that susceptible mice exhibit depressive‐like behavior compared to the control group, whereas resilient mice show no significant behavioral alterations. In addition, we observed that among the mice subjected to CSDS in this study, 42.7% were susceptible, 17.4% were resilient, and the corresponding control group showed an 87.4% eligibility rate (Figure S1H, Supporting Information).

Analysis of *Cisd1* mRNA expression in depression‐related brain regions after chronic stress (Figure S2A, Supporting Information) revealed increased expression in the mPFC, with no significant changes in the hippocampus or nucleus accumbens (NAc) (Figure S2B, Supporting Information). According to predictions from the STRING database (cn.string‐db.org/), CISD1 primarily associates with redox‐related proteins (Cisd3, Tomm70a, Nfs1, Ncoa4 and Ireb2) and mitochondrial complex I components (Ndufv1, Ndufs5 and Ndufb6) (Figure S2C, Supporting Information). The NAD⁺/NADH ratio partially represents the functional status of mitochondrial complex I.^[^ ^26^ ^]^ Compared with the control group, the NAD^+^/NADH ratio was significantly reduced in the mPFC and hippocampus of susceptible mice, while no significant change was observed in the NAc (Figure S2D, Supporting Information). In addition, we measured the expression of antioxidant genes hemeoxygenase‐1 (*Hmox1*), superoxide dismutase‐1 (*Sod1*) and superoxide dismutase‐2 (*Sod2*). The results showed that the expression of these genes was reduced in the mPFC of susceptible mice, whereas no significant changes were observed in resilient mice (Figure S2E, Supporting Information). Furthermore, no significant alterations were detected in the hippocampus or NAc of susceptible mice (Figure S2F,G, Supporting Information).

In addition, we observed increased ROS levels and a higher number of microglia in the mPFC of mice (Figure S2H–J, Supporting Information). Complex I catalyzes NADH oxidation and Coenzyme Q (CoQ) reduction to CoQH_2_ via proton‐coupled electron transfer, playing a key role in redox and stress signaling.^[^ ^27^ ^]^ We found that mitochondrial complex I activity was reduced in the mPFC of susceptible mice (Figure S2K, Supporting Information). Similarly, complex II, which oxidizes succinate to fumarate in the TCA cycle and transfers electrons to the CoQ pool to generate CoQH_2_,^[^ ^28^ ^]^ also showed reduced activity in the mPFC of susceptible mice (Figure S2L, Supporting Information). In addition, GAPDH, a key enzyme in glycolysis, exhibits elevated activity in susceptible mice (Figure S2M, Supporting Information). We also observed a progressive increase in CISD1 protein expression on days 7 and 10 following chronic stress (Figure S2N,O, Supporting Information). These results suggest that chronic stress increases CISD1 expression, leading to oxidative stress in the mPFC of susceptible mice.

### 2.2 Inhibition of CISD1 Ameliorates Stress‐Induced Depressive‐Like Behavior in Mice

**Figure 1:**
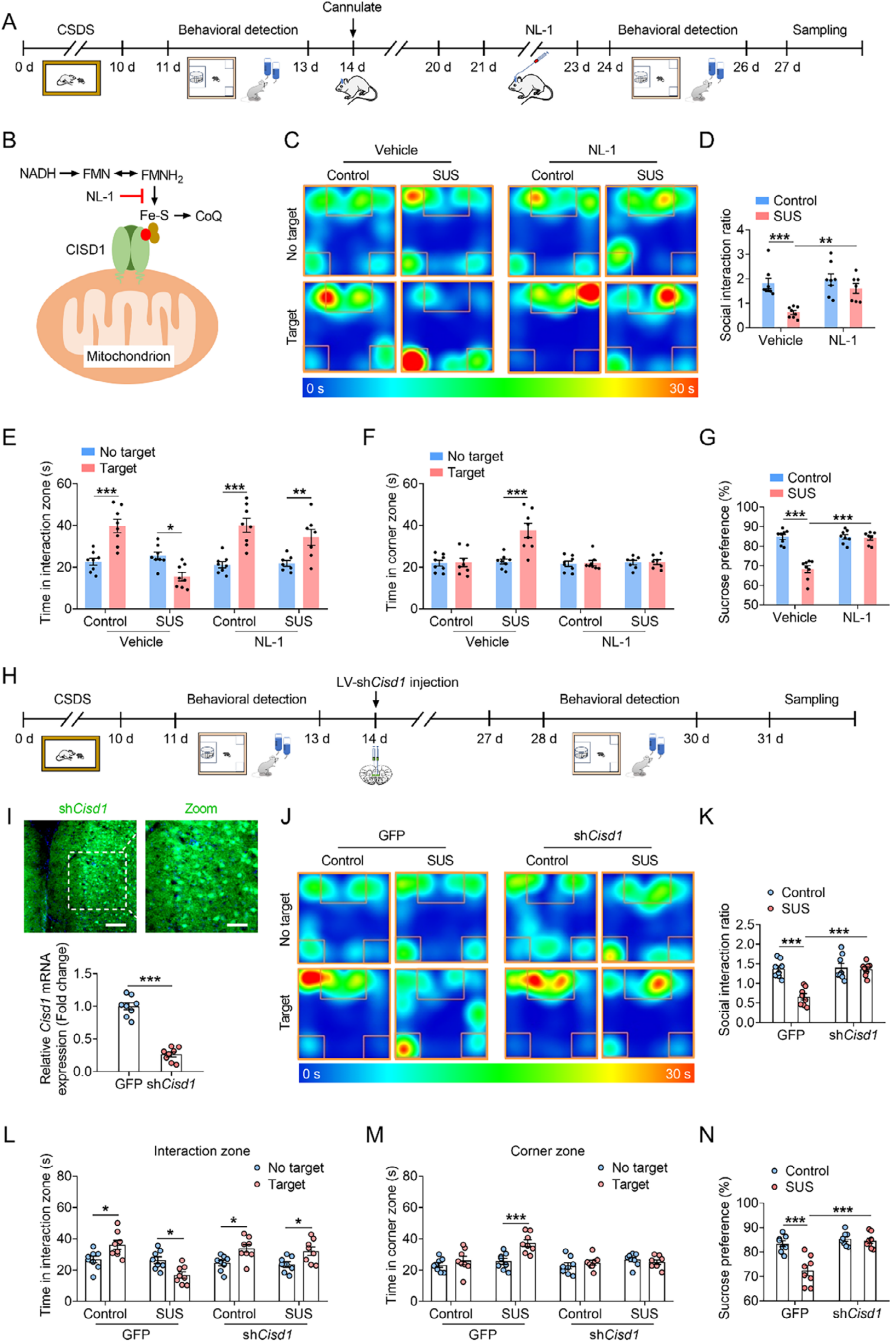
Pharmacological inhibition or knockdown of CISD1 improves depressive‐like behavior in mice. A) Experimental timelines for behavioral testing following NL‐1 (CISD1 inhibitor) treatment. B) Schematic diagram showing the pharmacological mechanism of action of NL‐1: NL‐1 inhibits Fe‐S cluster‐dependent electron transfer of CISD1. C–G) Typical heat plot (C), social interaction ratio (D), time in interaction zone (D), time in corner zone (E), percentage of sucrose preference (G) of mice from control‐vehicle, susceptible (SUS)‐vehicle, control‐NL‐1 and SUS‐NL‐1 groups (n = 7–8). H) Experimental timelines for behavioral testing following LV‐U6‐shRNA‐*Cisd1*‐CMV‐GFP (LV‐sh*Cisd1*) viruses injected into the mPFC of control and SUS mice. I) Representative immunofluorescence image of viruses injection site in the mPFC, and mRNA expression of *Cisd1* from the GFP and sh*Cisd1* group (n = 8). Scale bars indicate 200 µm (left) and 50 µm (right). J–N) Typical heat plot (J), social interaction ratio (K), time in interaction zone (L), time in corner zone (M), percentage of sucrose preference (N) of mice from control‐GFP, SUS‐GFP, control‐sh*Cisd1* and SUS‐sh*Cisd1* groups (n = 8). Data are presented as the mean ± SEM. Statistical analysis by unpaired Student's *t*‐test in (I) or two‐way ANOVA with Bonferroni's post hoc test (D–G, K–N). *p < 0.05, **p < 0.01, ***p < 0.001.

To further investigate the specific role of CISD1 in depressive‐like behavior in mice, the CISD1 inhibitor NL‐1 was administered for 3 consecutive days via microinjection into the mPFC of mice (**Figure** **1**A,B). NL‐1 inhibits electron transfer of iron‐sulfur (Fe‐S) clusters of CISD1.^[^ ^29^ ^]^ Behavioral tests demonstrated that compared with vehicle group, NL‐1 treatment significantly increased the social interaction ratio, prolonged the time in interaction zone, reduced the time spent in corner zone, and increased sucrose preference in susceptible (SUS) mice (Figure 1C–G).

Furthermore, we performed brain region‐specific, lentivirus (LV)‐mediated knockdown of CISD1 by microinjecting sh*Cisd1* viruses into the mPFC of mice and subsequently conducted behavioral tests (Figure 1H,I). The results showed that CISD1 knockdown significantly increased the social interaction ratio, interaction time, and sucrose preference rate in SUS mice (Figure 1J–N). To further explore the role of CISD1 in stress response, we microinjected overexpression viruses (LV‐*Cisd1*) into the mPFC of mice, subjected them to subthreshold social defeat stress (SSDS), and subsequently performed behavioral tests (Figure S3A,B, Supporting Information). Compared with the SSDS‐GFP group, the social interaction ratio, interaction time and sucrose preference rate were significantly decreased in SSDS‐*Cisd1* group (Figure S3C–F, Supporting Information). These results suggest that pharmacological inhibition or genetic knockdown of CISD1 ameliorates depressive‐like behavior in mice, whereas overexpression of CISD1 promotes stress susceptibility in mice.

### 2.3 Knockdown of CISD1 Ameliorates Stress‐Induced Microglial Inflammatory Activation

**Figure 2:**
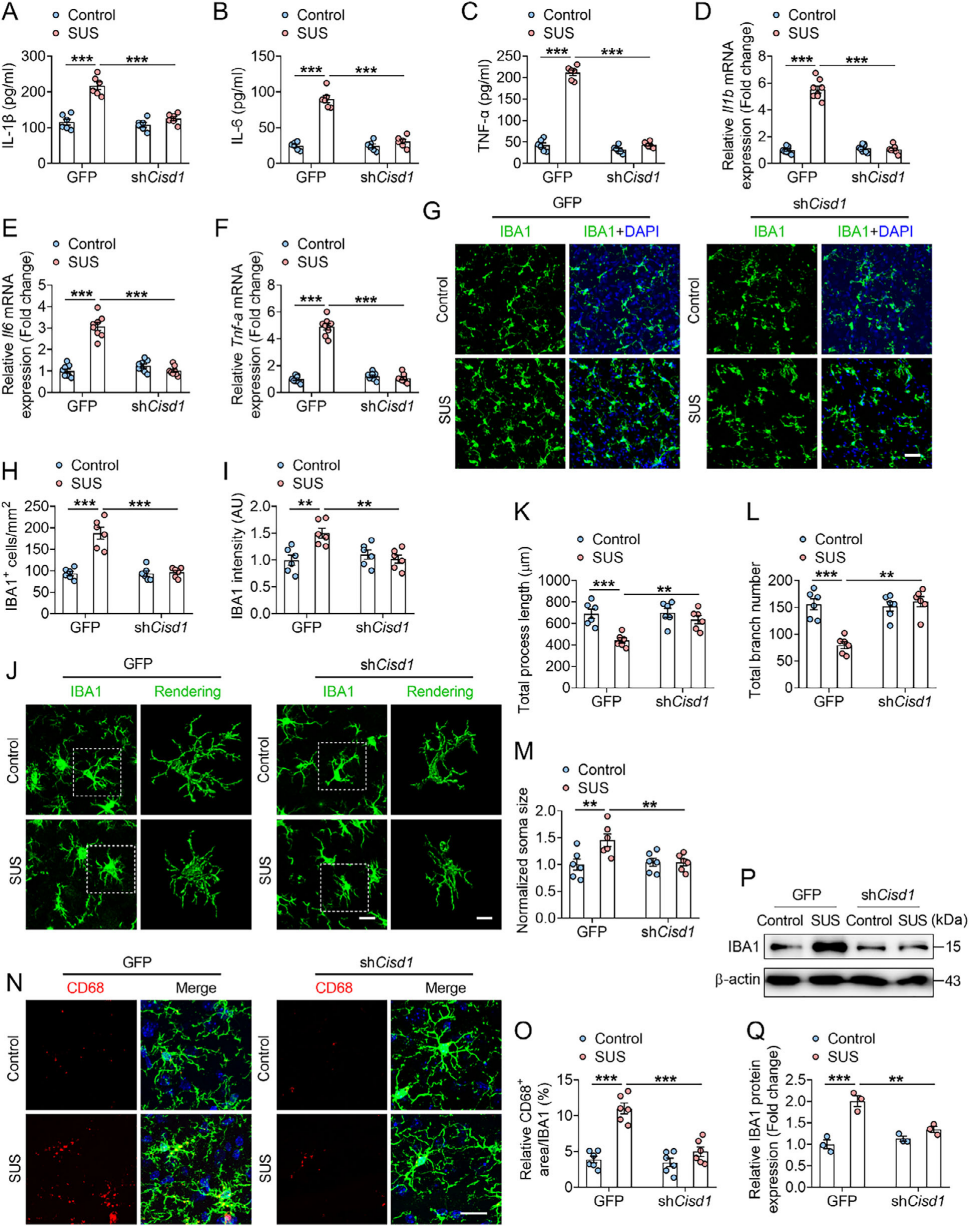
Knockdown of CISD1 ameliorates microglial inflammatory activation. A–C) The inflammatory cytokines IL‐1β (A), IL‐6 (B) and TNF‐α (C) levels in the mPFC from control‐GFP, susceptible (SUS)‐GFP, control‐sh*Cisd1* and SUS‐sh*Cisd1* mice (n = 6). D–F) The mRNA expression of inflammatory cytokines *Il1b* (D), *Il6* (E) and *Tnf‐a* (F) in the mPFC from control‐GFP, SUS‐GFP, control‐sh*Cisd1* and SUS‐sh*Cisd1* mice (n = 8). G–I) Representative immunofluorescence images (G) and quantitative analyses of the number of microglia (H) and the microglia (IBA1, green) intensity (I) in control‐GFP, SUS‐GFP, control‐sh*Cisd1* and SUS‐sh*Cisd1* mice (n = 6). Scale bars indicate 100 µm. J–M) Representative confocal images and 3D reconstruction images (J) of microglia and quantitative analyses of total process length (K), total branch number (L) and normalized soma size (M) from control‐GFP, SUS‐GFP, control‐sh*Cisd1* and SUS‐sh*Cisd1* mice (n = 6). Scale bars indicate 50 µm (left) and 10 µm (right). N, O) Representative images (N) and analyses (O) of the co‐labeling IBA1 (green) and CD68 (red) in the mPFC of control‐GFP, SUS‐GFP, control‐sh*Cisd1* and SUS‐sh*Cisd1* mice (n = 6). Scale bars indicate 10 µm. P,Q) Representative western blots (P) and analyses (Q) of IBA1 in the mPFC of control‐GFP, SUS‐GFP, control‐sh*Cisd1* and SUS‐sh*Cisd1* mice (n = 3). Data are presented as the mean ± SEM. Statistical analysis by two‐way ANOVA with Bonferroni's post hoc test (A–F, H, I, K–M, O, Q). **p < 0.01, ***p < 0.001.

Given that CISD1 is functionally associated with redox regulation and mitochondrial function,^[^ ^21^, ^30^ ^]^ we further investigated its relationship with neuroinflammation. The results showed that CISD1 knockdown reduced the protein levels and mRNA expression of IL‐1β, IL‐6, and TNF‐α (**Figure** **2**A–F). Furthermore, immunofluorescence experiments showed that chronic stress increased the number and fluorescence intensity of IBA1‐positive cells (a marker of microglia), while CISD1 knockdown reversed these effects (Figure 2G–I). In addition, CISD1 knockdown reversed chronic stress‐induced decrease in microglial process length and branch number, as well as an increase in soma size (Figure 2J–M). Cluster of Differentiation 68 (CD68) is a well‐established marker of microglial activation.^[^ ^31^ ^]^ Co‐labeling of CD68 with microglia revealed that chronic stress increased CD68 expression in microglia, whereas CISD1 knockdown reversed this effect and reduced the stress‐induced upregulation of IBA1 protein (Figure 2N–Q). These results suggest that CISD1 knockdown improves chronic stress‐induced microglial inflammatory activation.

### 2.4 Microglial CISD1 Mediates Depressive‐Like Behavior in Mice

**Figure 3:**
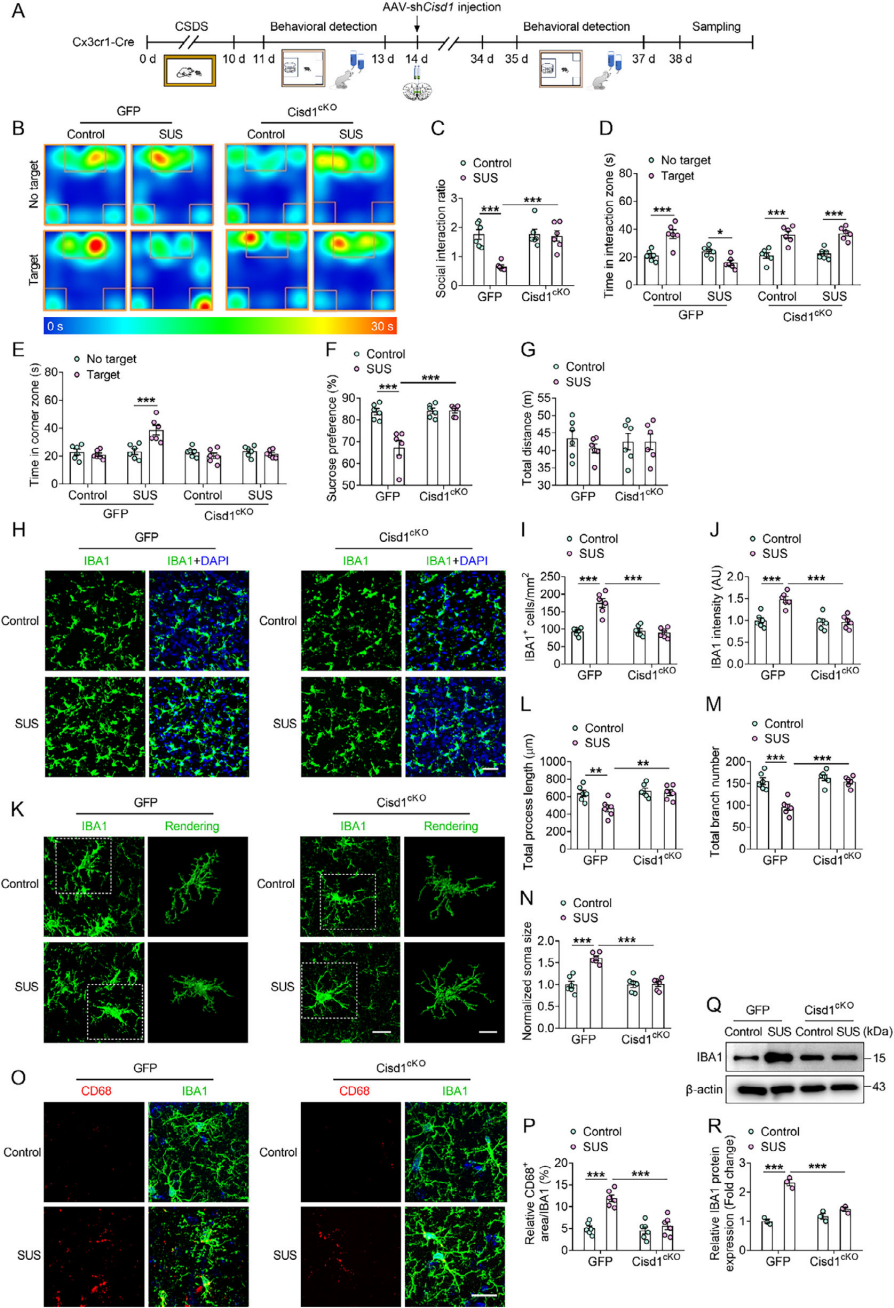
Microglia‐specific CISD1 knockout ameliorates depressive‐like behavior and microglial inflammatory activation in mice. A) Cx3cr1‐Cre mice were exposed to chronic social defeat stress, then received microinjections of AAV‐CMV‐DIO‐GFP‐miRNA30shRNA(*Cisd1*)‐WPRE (AAV‐sh*Cisd1*) viruses into the mPFC to induce microglial‐specific CISD1 knockout (Cisd1^cKO^), and finally underwent behavioral assessments. B–G) Typical heat plot (B), social interaction ratio (C), time in interaction zone (D), time in corner zone (E), percentage of sucrose preference (F) and total distance (G) of mice from control‐GFP, susceptible (SUS)‐GFP, control‐Cisd1^cKO^ and SUS‐Cisd1^cKO^ groups (n = 6). H–J) Representative immunofluorescence images (H) and quantitative analyses of the number of microglia (I) and the microglia (IBA1, green) intensity (J) in control‐GFP, SUS‐GFP, control‐ Cisd1^cKO^ and SUS‐Cisd1^cKO^ mice (n = 6). Scale bars indicate 100 µm. K–N) Representative confocal images and 3D reconstruction images (K) of microglia and quantitative analyses of total process length (L), total branch number (M) and normalized soma size (N) from control‐GFP, SUS‐GFP, control‐Cisd1^cKO^ and SUS‐Cisd1^cKO^ mice (n = 6). Scale bars indicate 50 µm (left) and 10 µm (right). O,P) Representative images (O) and analyses (P) of the co‐labeling IBA1 (green) and CD68 (red) in the mPFC of control‐GFP, SUS‐GFP, control‐ Cisd1^cKO^ and SUS‐Cisd1^cKO^ mice (n = 6). Scale bars indicate 10 µm. Q,R) Representative western blots (Q) and analyses (R) of IBA1 in the mPFC of control‐GFP, SUS‐GFP, control‐ Cisd1^cKO^ and SUS‐Cisd1^cKO^ mice (n = 3). Data are presented as the mean ± SEM. Statistical analysis by two‐way ANOVA with Bonferroni's post hoc test (C–G, I, J, L–N, P, R). *p < 0.05, **p < 0.01, ***p < 0.001.

To further clarify the cell types exhibiting differential changes in CISD1 expression, microglia, astrocytes, and neurons were freshly isolated from the mPFC tissues through fluorescence‐activated cell sorting (Figure S4A, Supporting Information). Compared with the control group, CISD1 expression was significantly increased in microglia, whereas no significant changes were observed in astrocytes or neurons (Figure S4B–F, Supporting Information). Therefore, we utilized Cx3cr1‐Cre mice to conditional knockout of microglial CISD1 (Cisd1^cKO^) by microinjecting microglia targeting adeo‐associated viruses (AAV)‐sh*Cisd1* into the mPFC and verified the knockout efficiency (**Figure** **3**A; Figure S4G, Supporting Information). Subsequently, we examined behavioral changes in the mice and collected tissues (Figure 3A). Compared with the SUS‐GFP group, the SUS‐Cisd1^cKO^ group exhibited improved social interaction behavior and increased sucrose preference (Figure 3B–F). Moreover, no significant changes were observed in the locomotor activity of mice in any group (Figure 3G). These results suggest that increased expression of CISD1 in microglia mediates depressive‐like behavior in mice.

Then, we examined microglial inflammatory activation in the mPFC by immunofluorescence assays. Compared with the SUS‐GFP group, SUS‐Cisd1^cKO^ group exhibited a significant decrease in both the number and fluorescence intensity of microglia (Figure 3H–J). Additionally, SUS‐Cisd1^cKO^ group demonstrated increased total process length and branch number, accompanied by reduced soma size of microglia (Figure 3K–N). Moreover, conditional knockout of CISD1 significantly attenuated the chronic stress‐induced upregulation of CD68 in microglia and IBA1 protein levels (Figure 3O–R). These results suggest that microglia‐specific CISD1 knockout inhibits microglial inflammatory activation and alleviates depressive‐like behavior in mice.

### 2.5 Conditional Knockout of CISD1 Restores the Microglial Metabolic Phenotype

The Fe‐S cluster of CISD1 acts as a redox enzyme catalyzing electron transfer from NADH in cytosol to oxygen or coenzyme Q (CoQ).^[^ ^17^ ^]^ Therefore, we hypothesized that the increased expression of CISD1 in microglia may affect pyruvate metabolism (Figure S5A, Supporting Information). The lactate content in the mPFC did not show a significant change in susceptible mice compared to control mice (Figure S5B, Supporting Information). Furthermore, microglia were freshly isolated from mPFC tissues, and their glycolytic activity was subsequently assessed (Figure S5C, Supporting Information). Compared with the control mice, the lactate content and lactate dehydrogenase (LDH) activity were significantly increased in microglia of SUS mice (Figure S5D,E, Supporting Information). Moreover, the activity of pyruvate dehydrogenase (PDH) was reduced in SUS mice (Figure S5F, Supporting Information). These results suggest that glycolytic activity in microglia is significantly enhanced in the mPFC of SUS mice.

**Figure 4:**
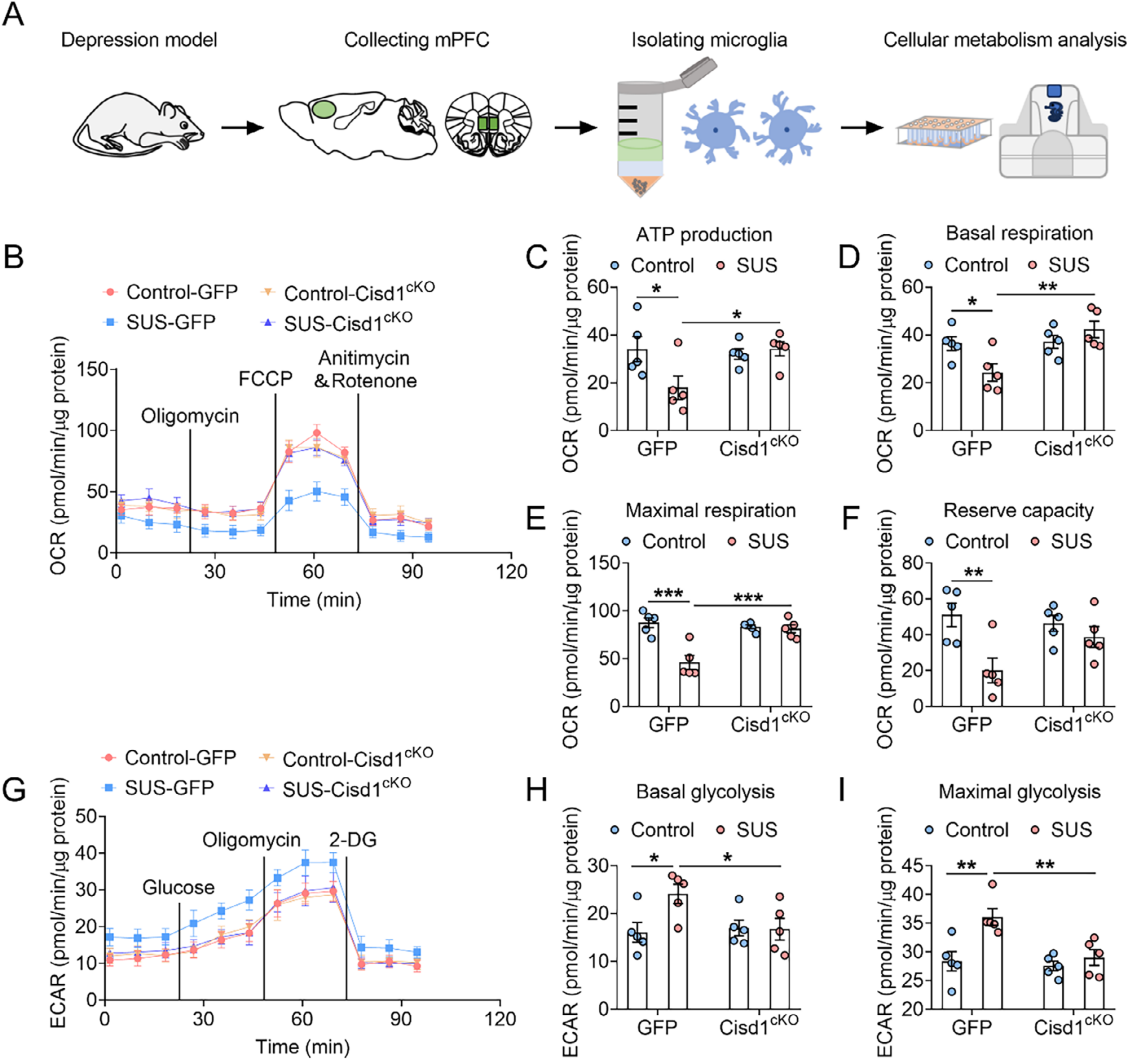
Microglia‐specific CISD1 knockout restores the microglial metabolic phenotype. A) Schematic diagram of the experimental process shows that microglia were isolated from the mPFC of mice and then analyzed for metabolic activity. B) The mitochondrial stress test kit showing the change of oxygen consumption rate (OCR) in microglia from control‐GFP, SUS‐GFP, control‐Cisd1^cKO^ and SUS‐Cisd1^cKO^ mice (n = 5). C–F) ATP production (C), Basal respiration (D), maximal respiration (E) and reserve capacity (F) in control‐GFP, SUS‐GFP, control‐Cisd1^cKO^ and SUS‐Cisd1^cKO^ groups (n = 5). G) The glycolysis stress test kit showing the change of extracellular acidification rate (ECAR) in microglia from control‐GFP, SUS‐GFP, control‐Cisd1^cKO^ and SUS‐Cisd1^cKO^ mice (n = 5). H, I) Basal glycolysis (H) and maximal glycolysis (I) in control‐GFP, SUS‐GFP, control‐Cisd1^cKO^ and SUS‐Cisd1^cKO^ groups (n = 5). Data are presented as the mean ± SEM. Statistical analysis by two‐way ANOVA with Bonferroni's post hoc test (C–F, H, I). *p < 0.05, **p < 0.01, ***p < 0.001.

To further investigate these findings, we characterized the metabolic phenotype in microglia using cellular energy metabolism analyzer (**Figure** **4**A). Compared with the control‐GFP group, the oxidative phosphorylation function of microglia in the SUS‐GFP group was decreased, manifested as decreased ATP production, basal respiration, maximal respiration and reserve capacity. However, in the SUS‐Cisd1^cKO^ group, ATP production, basal respiration and maximal respiration were restored to normal levels (Figure 4B–F). Additionally, compared with the control group, microglial glycolysis levels were significantly increased in the SUS‐GFP group, manifested as increased basal and maximal glycolysis capacity. However, the glycolysis levels were restored in the SUS‐Cisd1^cKO^ group (Figure 4G–I). These results suggest that microglia‐specific CISD1 knockout improves the microglial metabolic phenotype.

Immunofluorescence with dihydroethidium (DHE), a marker of ROS, and IBA1 was performed (Figure S5G, Supporting Information). ROS levels in the mPFC were elevated in the SUS‐GFP group compared with control‐GFP group and were reduced after CISD1 knockout (Figure S5H, Supporting Information). Furthermore, compared with the SUS‐GFP group, the NAD^+^/NADH ratio, the activities of mitochondrial complexes I and II, and the expression of antioxidant genes (*Hmox1*, *Sod1* and *Sod2*) were restored in the SUS‐Cisd1^cKO^ group (Figure S5I–L, Supporting Information). These results suggest that microglia‐specific CISD1 knockout ameliorates oxidative stress in the mPFC of mice.

### 2.6 Inhibition of CISD1 Restores the Microglial Metabolic Phenotype Induced by LPS+ATP Treatment

**Figure 5:**
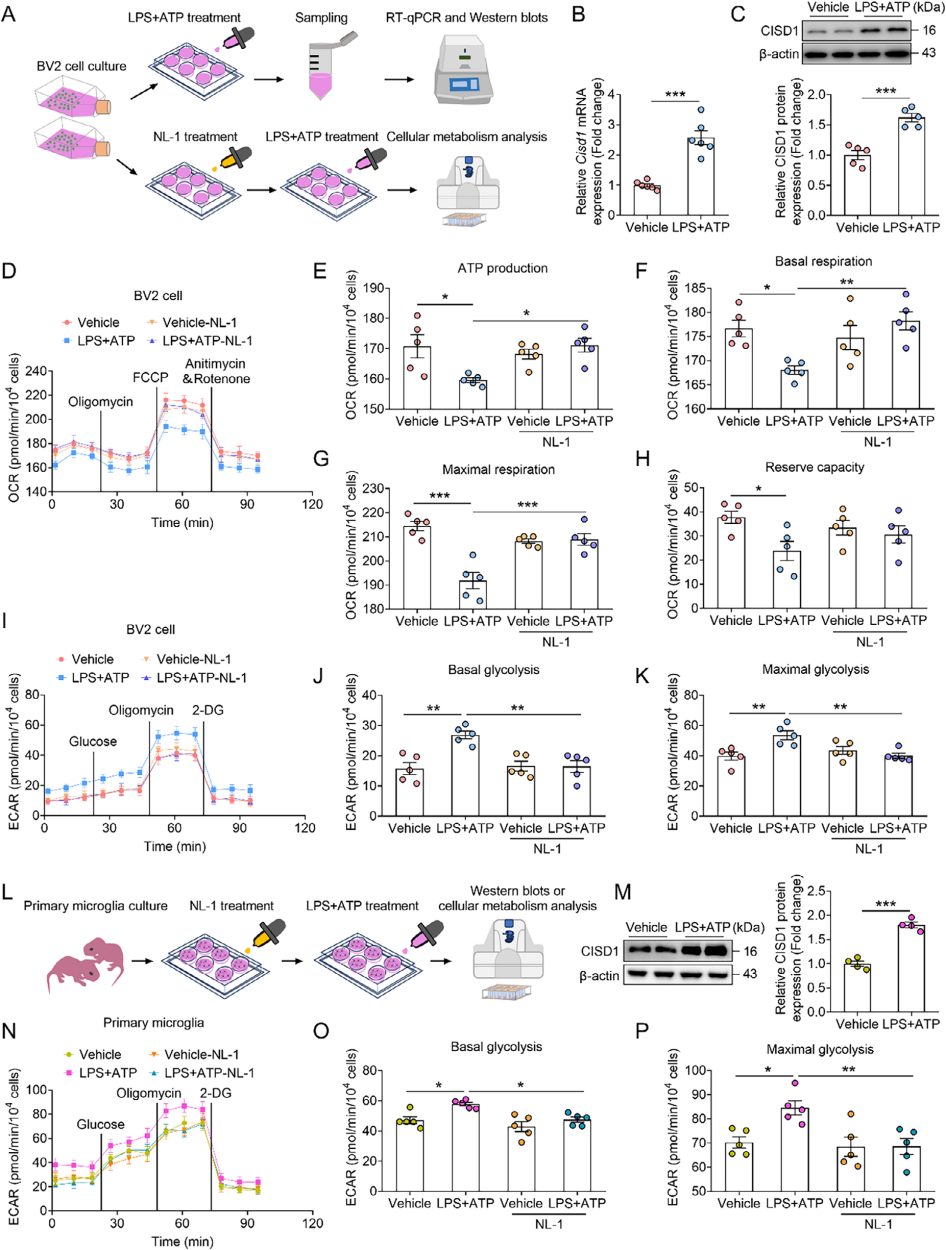
Pharmacological inhibition of CISD1 restores the microglial metabolic phenotype. A) Schematic diagram illustrates the treatment procedure for cultured BV2 cells. B,C) The mRNA (B) and protein (C) expression of CISD1 in vehicle and LPS+ATP group (n = 5–6). D) The mitochondrial stress test kit showing the change of oxygen consumption rate (OCR) in BV2 cells from vehicle, LPS+ATP, vehicle‐NL‐1 and LPS+ATP‐NL‐1 groups (n = 5). E–H) ATP production (E), Basal respiration (F), maximal respiration (G) and reserve capacity (H) in vehicle, LPS+ATP, vehicle‐NL‐1 and LPS+ATP‐NL‐1 groups (n = 5). I) The glycolysis stress test kit showing the change of extracellular acidification rate (ECAR) in BV2 cells from vehicle, LPS+ATP, vehicle‐NL‐1 and LPS+ATP‐NL‐1 groups (n = 5). J,K) Basal glycolysis (J) and maximal glycolysis (K) in vehicle, LPS+ATP, vehicle‐NL‐1 and LPS+ATP‐NL‐1 groups (n = 5). L) Schematic diagram illustrates the treatment procedure for cultured primary microglia cells. M) Representative western blots and analyses of CISD1 in the vehicle and LPS+ATP group (n = 4). N) The glycolysis stress test kit showing the change of extracellular acidification rate (ECAR) in primary microglia cells from vehicle and LPS+ATP, vehicle‐NL‐1 and LPS+ATP‐NL‐1 groups (n = 5). O,P) Basal glycolysis (O) and maximal glycolysis (P) in vehicle, LPS+ATP, vehicle‐NL‐1 and LPS+ATP‐NL‐1 groups (n = 5). Data are presented as the mean ± SEM. Statistical analysis by unpaired Student's t test in (B, C, M) or one‐way ANOVA with Bonferroni's post hoc test (E–H, J, K, O, P). *p < 0.05, **p < 0.01, ***p < 0.001.

LPS+ATP treatment is commonly used to mimic the activation state of microglia under stress.^[^ ^32^, ^33^ ^]^ BV2 cells were treated with LPS+ATP, and samples were collected for biochemical analysis. Alternatively, cells were pretreated with the CISD1 inhibitor NL‐1 prior to LPS+ATP stimulation, followed by cellular metabolism measurement (**Figure** **5**A). Compared with the vehicle group, LPS+ATP treatment significantly increased the mRNA and protein expression of CISD1 (Figure 5B,C). These results suggest that inflammatory stress promotes CISD1 expression. Furthermore, compared with the vehicle group, LPS+ATP treatment significantly inhibited mitochondrial oxidative phosphorylation, while these inhibitory effects were reversed in the LPS+ATP‐NL‐1 group, especially ATP production, basal respiration and maximal respiration (Figure 5D–H). We also found that LPS+ATP‐induced increase in glycolytic levels of BV2 cells was abolished by NL‐1 administration (Figure 5I–K). These findings indicate that LPS+ATP enhances both mitochondrial oxidative phosphorylation and glycolytic activity in BV2 cells, whereas NL‐1 treatment reverses these effects.

To further investigate the role of CISD1 in inflammatory activation, we cultured primary microglia and treated them with NL‐1 and LPS+ATP, then microglia were collected for western blots or cellular metabolism measurement (Figure 5L). Compared with the vehicle group, LPS+ATP treatment significantly increased CISD1 protein expression in primary microglia (Figure 5M). It also enhanced both basal and maximal glycolysis, and these effects were reversed by NL‐1 (Figure 5N–P). In conclusion, these results suggest that CISD1 mediates inflammatory activation by altering the microglial metabolic phenotype.

### 2.7 Pioglitazone Ameliorates Chronic Stress‐Induced Depressive‐Like Behavior in Mice

**Figure 6:**
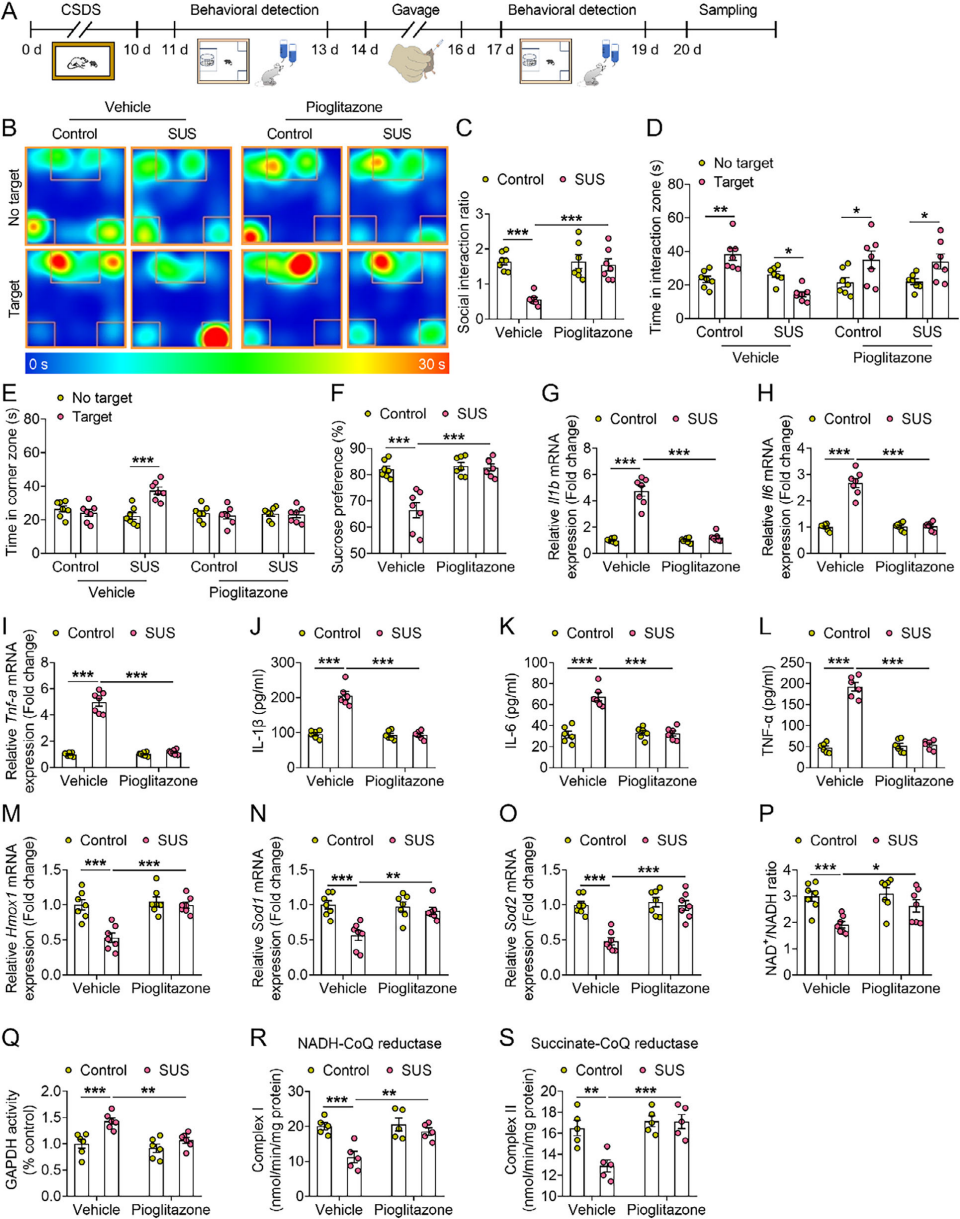
Pioglitazone treatment alleviates depressive‐like behavior and neuroinflammation in mice. A) Experimental timelines for behavioral testing following pioglitazone treatment. B–F) Typical heat plot (B), social interaction ratio (C), time in interaction zone (D), time in corner zone (E), percentage of sucrose preference (F) of mice from control‐vehicle, susceptible (SUS)‐vehicle, control‐pioglitazone and SUS‐pioglitazone groups (n = 7). G–I) The mRNA expression of inflammatory cytokines *Il1b* (G), *Il6* (H) and *Tnf‐a* (I) in the mPFC from control‐GFP, SUS‐GFP, control‐pioglitazone and SUS‐pioglitazone mice (n = 7). J‐L) The inflammatory cytokines IL‐1β (J), IL‐6 (K) and TNF‐α (L) levels in the mPFC from control‐GFP, SUS‐GFP, control‐pioglitazone and SUS‐pioglitazone mice (n = 6). M–O) The mRNA expression of antioxidant genes heme oxygenase‐1 (*Hmox1*, M), superoxide dismutase 1 (*Sod1*, N) and *Sod2* (O) in the mPFC from control‐GFP, SUS‐GFP, control‐pioglitazone and SUS‐pioglitazone mice (n = 7). P) NAD^+^/NADH ratio in the mPFC from control‐GFP, SUS‐GFP, control‐pioglitazone and SUS‐pioglitazone mice (n = 7). Q) The activity of glyceraldehyde‐3‐phosphate dehydrogenase (GAPDH) in the mPFC from control‐GFP, SUS‐GFP, control‐pioglitazone and SUS‐pioglitazone mice (n = 6). R) The activity of complex I in the mPFC from control‐GFP, SUS‐GFP, control‐pioglitazone and SUS‐pioglitazone mice (n = 5). S) The activity of complex II in the mPFC from control‐GFP, SUS‐GFP, control‐pioglitazone and SUS‐pioglitazone mice (n = 5). Data are presented as the mean ± SEM. Statistical analysis by two‐way ANOVA with Bonferroni's post hoc test (C–S). *p < 0.05, **p < 0.01, ***p < 0.001.

CISD1 is a binding site of pioglitazone in vivo.^[^ ^34^ ^]^ Moreover, clinical studies suggested that oral pioglitazone (15–30 mg kg^−1^ per day) improves depressive symptoms in patients with depression.^[^ ^35^ ^]^ According to the human‐mouse dose conversion, a mouse dose of 3.1–6.2 mg kg^−1^ was determined.^[^ ^36^ ^]^ FST and TST are rapid, black‐box assays developed decades ago to screen compounds for antidepressant activity.^[^ ^37^ ^]^ Different doses of pioglitazone were administered to mice via gavage for 3 consecutive days, and 1 h after the final administration, FST and TST were performed (Figure S6A, Supporting Information). Compared with the vehicle group, the immobility time was significantly reduced at doses of 4 and 6 mg kg^−1^ in mice (Figure S6B,C, Supporting Information). Therefore, we selected the 4 mg kg^−1^ dose for subsequent studies. After 3 consecutive days of intragastric pioglitazone administration to SUS mice, the social interaction and sucrose preference tests were performed (**Figure** **6**A). Compared with the SUS‐vehicle group, the SUS‐pioglitazone group showed improved social interaction behavior and sucrose preference (Figure 6B–F). In addition, compared with the SSDS‐*Cisd1*‐vehicle group, the social interaction ratio, interaction time and sucrose preference rate were significantly increased in SSDS‐*Cisd1*‐pioglitazone group (Figure S6D‐H, Supporting Information). These results indicate that pioglitazone treatment significantly reduced stress susceptibility in mice.

Furthermore, compared with the SUS‐vehicle group, the SUS‐pioglitazone group showed decreased expression of inflammatory factors IL‐1β, IL‐6, and TNF‐α, and increased expression of antioxidant genes *Hmox1*, *Sod1*, and *Sod2* in the mPFC of mice (Figure 6G–O). In addition, pioglitazone treatment restored the NAD⁺/NADH ratio and normalized GAPDH activity disrupted by chronic stress (Figure 6P,Q). Moreover, pioglitazone increased the activities of mitochondrial complexes I and II (Figure 6R,S). These results suggest that pioglitazone alleviates depressive‐like behavior in mice by inhibiting CISD1‐mediated neuroinflammation and oxidative stress.

### 2.8 Pioglitazone Restores the Microglial Metabolic Phenotype Induced by LPS+ATP Treatment

Some studies have reported that pioglitazone exerts antidepressant effects by activating peroxisome proliferator‐activated receptor γ (PPAR‐γ).^[^ ^38^ ^]^ To determine whether the antidepressant effects of pioglitazone involves CISD1, cannulas were implanted into the mPFC of SUS mice, and the PPAR‐γ inhibitor T0070907 (10 µM, 1 µL per side) was infused daily for 3 consecutive days. Pioglitazone was administered by gavage 6 h after each infusion, and behavioral testing was performed after 3 days of treatment (Figure S7A, Supporting Information). Our results showed that PPAR‐γ inhibition did not alter the antidepressant effects of pioglitazone (Figure S7B–E, Supporting Information). These results suggest that pioglitazone exerts antidepressant effects in the mPFC of mice via a PPAR‐γ‐independent pathway.

**Figure 7:**
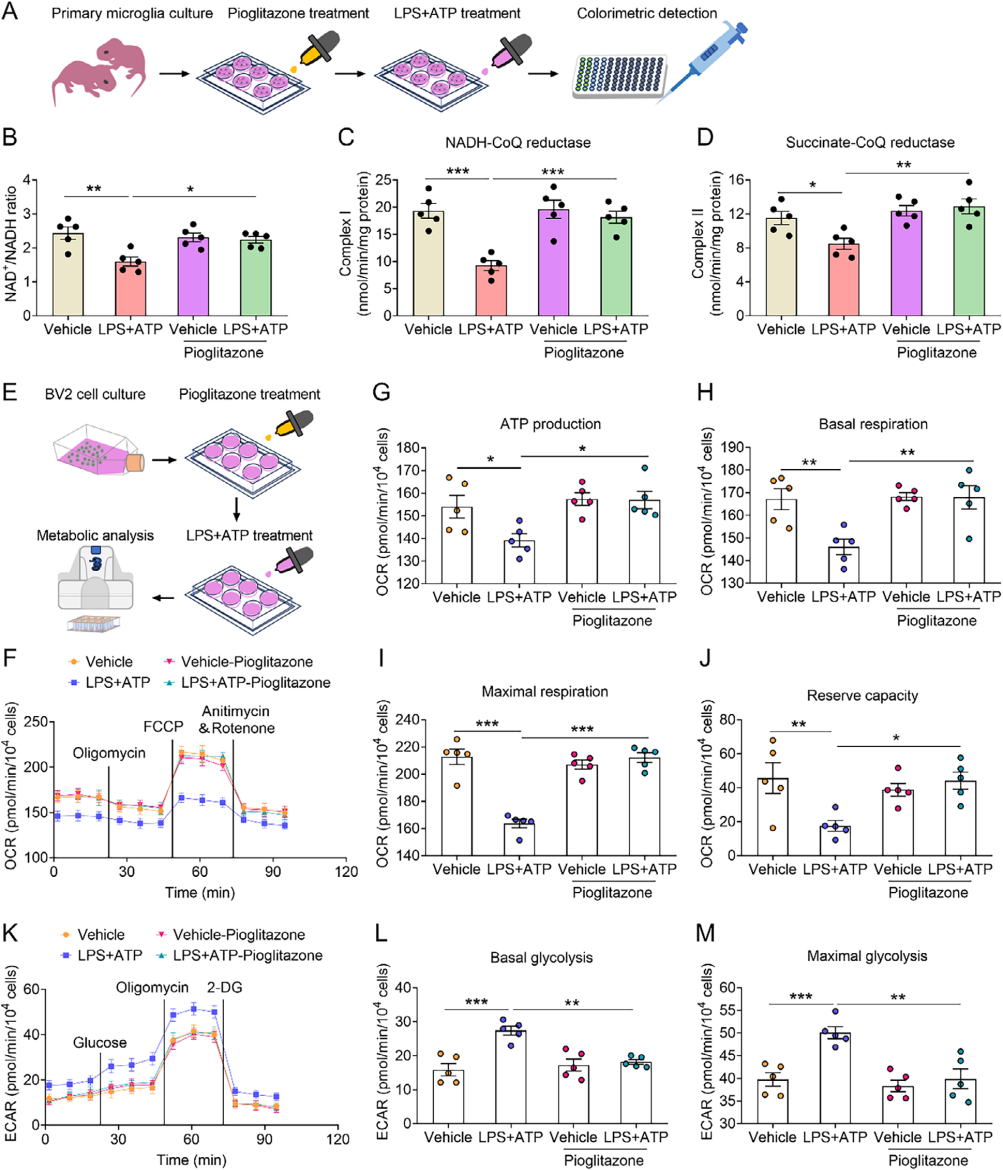
Pioglitazone treatment restores the microglial metabolic phenotype. A) Schematic diagram of the experimental procedure for primary microglia. B) NAD^+^/NADH ratio in vehicle, LPS+ATP, vehicle‐pioglitazone and LPS+ATP‐pioglitazone groups (n = 5). C) The activity of complex I in vehicle, LPS+ATP, vehicle‐pioglitazone and LPS+ATP‐pioglitazone groups (n = 5). D) The activity of complex II in vehicle, LPS+ATP, vehicle‐pioglitazone and LPS+ATP‐pioglitazone groups (n = 5). E) Schematic diagram of the experimental procedure for BV2 cell line. F) The mitochondrial stress test kit showing the change of oxygen consumption rate (OCR) in BV2 cells from vehicle, LPS+ATP, vehicle‐pioglitazone and LPS+ATP‐pioglitazone groups (n = 5). G–J) ATP production (G), basal respiration (H), maximal respiration (I) and reserve capacity (J) in vehicle, LPS+ATP, vehicle‐pioglitazone and LPS+ATP‐pioglitazone groups (n = 5). K) The glycolysis stress test kit showing the change of extracellular acidification rate (ECAR) in BV2 cells from vehicle, LPS+ATP, vehicle‐pioglitazone and LPS+ATP‐pioglitazone groups (n = 5). L, M) Basal glycolysis (L) and maximal glycolysis (M) in vehicle, LPS+ATP, vehicle‐pioglitazone and LPS+ATP‐pioglitazone groups (n = 5). Data are presented as the mean ± SEM. Statistical analysis by one‐way ANOVA with Bonferroni's post hoc test (B–D, G–J, L, M). *p < 0.05, **p < 0.01, ***p < 0.001.

To further investigate the effects of pioglitazone on microglial metabolism, we assessed metabolic alterations in primary microglia and BV2 cells treated with pioglitazone before LPS+ATP stimulation (**Figure** **7**A,E). LPS+ATP significantly reduced the NAD^+^/NADH ratio and the activities of mitochondrial complexes I and II in primary microglia, whereas pioglitazone reversed these effects (Figure 7B–D). Pioglitazone also restored LPS+ATP‐induced impairments in mitochondrial oxidative phosphorylation in BV2 cells, particularly in ATP production, basal respiration, maximal respiration, and reserve capacity (Figure 7F–J). In addition, pioglitazone attenuated the LPS+ATP‐induced increases in basal and maximal glycolysis (Figure 7K–M). These findings indicate that pioglitazone restores the metabolic phenotype of microglia under inflammatory stress.

## 3 Discussion

**Figure 8:**
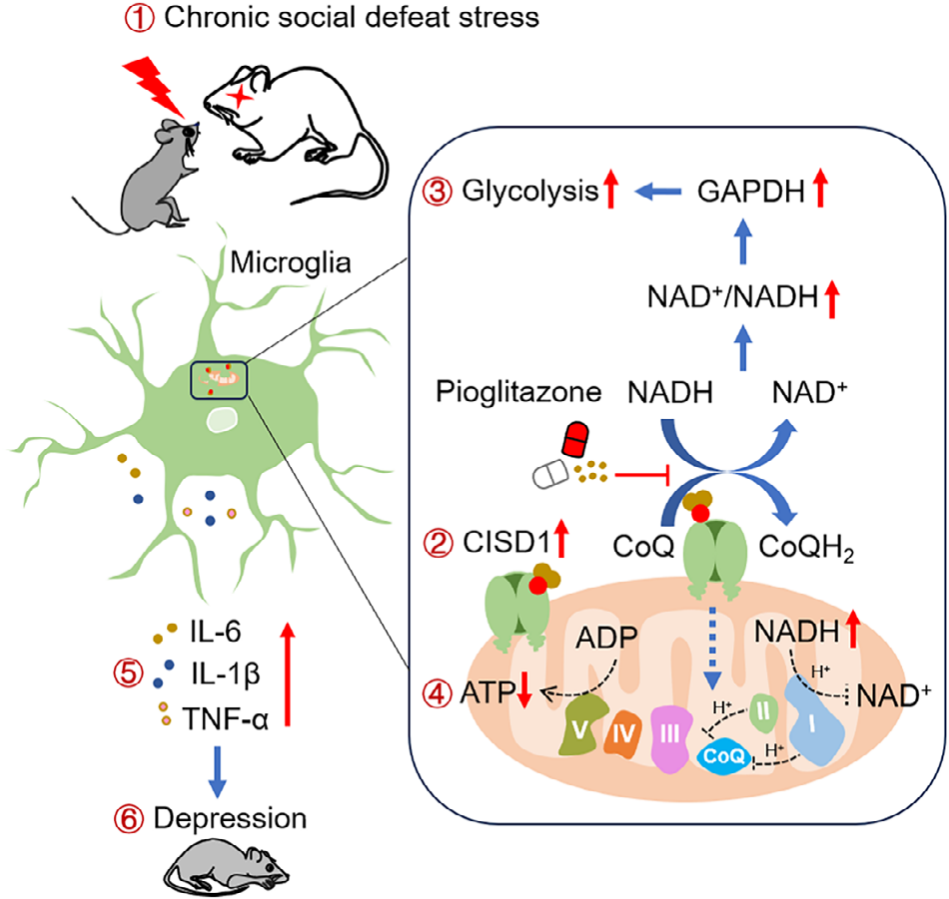
Schematic illustration of the proposed mechanism underlying CISD1‐mediated depressive‐like behavior in mice. Chronic stress increases CISD1 expression in microglia, promoting NADH oxidation and CoQ reduction. Increased NADH oxidation elevates the cytoplasmic NAD⁺/NADH ratio, thereby enhancing GAPDH activity and glycolytic flux. The increased reduction of CoQ may indirectly inhibit proton transfer through mitochondrial complexes I and II, leading to decreased ATP synthesis. This metabolic shift contributes to microglial inflammatory activation and depressive‐like behavior in mice. Pioglitazone acts on CISD1 to suppress NADH oxidation and CoQ reduction, thereby exerting antidepressant effects.

Our study demonstrates that chronic stress alters the microglial metabolic phenotype and reveals the critical role of CISD1 in neuroinflammatory processes (**Figure** **8**). Conditional knockout of microglial CISD1 in the mPFC ameliorates chronic stress‐induced depressive‐like behavior of mice, restores a normal metabolic phenotype, and reduces oxidative stress in microglia. Collectively, our study uncovers a previously unrecognized role of CISD1 in regulating microglial metabolism.

Accumulating evidence indicates that psychological stress triggers inflammatory responses within the central nervous system.^[^ ^25^, ^39^ ^]^ CSDS, a well‐established model of psychological stress, induces neuroimmune activation accompanied by progressive microglial activation and increased release of proinflammatory mediators.^[^ ^40^, ^41^ ^]^ To further examine stress‐related inflammation, we applied an LPS+ATP–induced inflammatory model and found that this stimulation also elevated CISD1 expression. These results suggest that both psychological and inflammatory stress converge on CISD1 to drive neuroinflammatory activation. Notably, CISD1 silencing mitigated microglial activation, indicating that CISD1 functions as a shared downstream effector mediating stress‐induced neuroinflammation.

CISD1 is characterized by a unique Fe‐S cluster‐coordinating CDGSH domain.^[^ ^21^ ^]^ This positions CISD1 protein as a key regulator of redox, electron and Fe‐S cluster transfer reactions, which are essential for the regulation of iron metabolism, mitochondrial respiration and ROS production.^[^ ^29^, ^42^ ^]^ Mitochondrial CISD1 accumulation blocks mitophagy, while genetic ablation or pharmacological inhibition of CISD1 rescues neurodegenerative phenotypes in Drosophila.^[^ ^21^ ^]^ CISD1 deletion (CISD1^−/−^) has been associated with cognitive impairments in mice, accompanied by elevated neuroinflammatory responses in the hippocampus.^[^ ^43^ ^]^ In contrast, inhibition of CISD1 in the primary somatosensory cortex has been shown to ameliorate traumatic brain injury–induced ferroptosis and cognitive dysfunction.^[^ ^44^ ^]^ These findings suggest that CISD1 may play distinct roles in different brain regions. In the present study, we demonstrated that chronic stress increased CISD1 expression in microglia of the mPFC, thereby promoting microglial inflammatory activation and depressive‐like behavior in mice. Collectively, these results indicate that CISD1 represents a potential intervention target for metabolism‐related neurological disorders.

The microglial metabolic phenotype influences their polarization state, microglial metabolic shift from oxidative phosphorylation to glycolysis leads to the pro‐inflammatory phenotype.^[^ ^45^, ^46^ ^]^ However, little is known about the specific molecules that regulate the metabolic shift between glycolysis and oxidative phosphorylation in microglia. CISD1 catalyzes the transfer of electrons from NADH to CoQ in the cytoplasm, generating NAD⁺ and CoQH_2_.^[^ ^17^ ^]^ During the sixth step of glycolysis, the key enzyme GAPDH converts glyceraldehyde‐3‐phosphate into 1,3‐bisphosphoglycerate while reducing NAD⁺ to NADH.^[^ ^47^, ^48^ ^]^ Thus, sufficient cytosolic NAD⁺ availability is essential for maintaining glycolytic flux. By oxidizing NADH, CISD1 helps replenish NAD⁺ in the cytoplasm, thereby sustaining glycolysis.

In addition, CISD1‐mediated CoQ reduction partially influences mitochondrial function.^[^ ^49^ ^]^ CoQ possesses antioxidant properties, and its activity depends on both its concentration and the ratio of reduced to oxidized forms (CoQH_2_/CoQ).^[^ ^50^ ^]^ Under oxidative stress, CoQ translocates from mitochondria to the plasma membrane, leading to mitochondrial dysfunction through reversal of the electron transport chain.^[^ ^49^ ^]^ Accordingly, CISD1 may indirectly impair the mitochondrial electron transport chain by facilitating CoQ transfer under stress conditions. In our study, chronic stress decreased the NAD⁺/NADH ratio and complex I activity. Impaired complex I leads to inefficient NADH oxidation, thereby reducing the mitochondrial NAD⁺/NADH ratio.^[^ ^26^ ^]^ Moreover, CoQ imbalance in mitochondria can drive reverse electron transport, further lowering the NAD⁺/NADH ratio.^[^ ^51^ ^]^ Although elevated CISD1 expression promotes NADH oxidation, which theoretically increases the NAD⁺/NADH ratio, mitochondria contain ≈70% of the total cellular NAD⁺,^[^ ^52^, ^53^ ^]^ thereby resulting in an overall decrease in the NAD⁺/NADH ratio.

The biological properties of CoQ suggest that its reduced form can function as an endogenous inhibitor of ferroptosis on cell membranes through its lipophilic free radical scavenging activity.^[^ ^54^ ^]^ Ferroptosis suppressor protein 1 (FSP1), a CoQ oxidoreductase, contributes to the antioxidant function of CoQ by regenerating its reduced form from the oxidized form on the cell membrane.^[^ ^55^, ^56^ ^]^ Under oxidative stress, the expression of FSP1 in mouse cortical tissue is significantly reduced, which leads to an increase in ferroptosis.^[^ ^57^ ^]^ Additionally, CoQ can be transferred from mitochondria to the plasma membrane under oxidative stress.^[^ ^49^ ^]^ Based on these findings, we speculate that elevated CISD1 expression may substitute for the function of FSP1 by reducing CoQ through NADH oxidation, thereby enhancing the cellular capacity to resist oxidative stress.

Previous studies have demonstrated that glycolysis provides a rapid ATP supply for activated microglia, supporting cell proliferation, cytokine synthesis, and ROS generation.^[^ ^58^ ^]^ In addition, glycolysis promotes NF‐κB signaling through the AMPK/mTOR/IKK axis, thereby enhancing the transcription of proinflammatory cytokines such as TNF‐α, IL‐1β, and IL‐6.^[^ ^59^ ^]^ Inhibition of glycolysis with 2‐DG significantly attenuates surgical trauma‐induced increases in IL‐1β and IL‐6 expression in the hippocampus.^[^ ^60^ ^]^ These findings suggest that CISD1 may enhance inflammatory cytokine production by facilitating glycolysis‐dependent activation of the AMPK/mTOR/IKK/NF‐κB pathway. Moreover, reversal of the mitochondrial electron transport chain promotes ROS generation and subsequent neuroinflammatory responses.^[^ ^61^ ^]^ We therefore propose that CISD1 indirectly contributes to electron transport chain reversal through its regulation of CoQ reduction, leading to ROS accumulation and neuroinflammation. However, the precise mechanisms by which CISD1 coordinates mitochondrial and cytosolic CoQ distribution to regulate cellular metabolism and redox homeostasis remain to be elucidated.

Several studies have demonstrated that oxidative stress is involved in the onset and progression of depression.^[^ ^62^, ^63^ ^]^ Microglia play a vital role in maintaining redox balance and regulating inflammatory activation through metabolic processes.^[^ ^64^, ^65^ ^]^ Moreover, alterations in microglial metabolism can lead to neurotoxic damage.^[^ ^66^ ^]^ In our study, chronic stress induced neuroinflammatory activation and oxidative stress in the mPFC of mice, whereas CISD1 knockdown reversed these phenotypes. Therefore, these alterations may represent secondary effects driven by changes in the microglial metabolic phenotype mediated by CISD1.

Pioglitazone, a thiazolidinedione‐class hypoglycemic agent, acts as an agonist of peroxisome proliferator‐activated receptor γ (PPAR‐γ).^[^ ^67^, ^68^ ^]^ However, several beneficial effects of thiazolidinediones cannot be fully attributed to PPAR‐γ activation.^[^ ^34^ ^]^ Previous studies have shown that pioglitazone binds to and stabilizes CISD1, which contains a labile [2Fe‐2S] cluster coordinated by His87 and three cysteines.^[^ ^34^, ^69^, ^70^ ^]^ Previous docking analyses identified two potential binding sites of pioglitazone with CISD1: site 1 (Lys89, Lys42, Arg76, Met44) and site 2 (Arg76, Glu93).^[^ ^71^ ^]^ Protonation of His87 promotes Fe–S cluster release,^[^ ^72^ ^]^ while pioglitazone binding enhances cluster stability—by reinforcing the Fe–N(His87) bond—up to tenfold.^[^ ^70^ ^]^ The above study indicates that after pioglitazone binds to CISD1, it increases the stability of the Fe‐S cluster by acting on the Fe‐N(His87) bond.

Clinical studies have shown that pioglitazone may be a promising drug candidate for the treatment of depression.^[^ ^35^, ^73^, ^74^, ^75^ ^]^ Animal studies also showed that pioglitazone administration improves cognitive and depressive phenotypes in mice, and alleviates brain atrophy.^[^ ^38^, ^76^, ^77^, ^78^, ^79^ ^]^ These studies suggest that pioglitazone has a potential antidepressant effect, but its antidepressant mechanism is unclear. Here, we demonstrated that the pioglitazone alters the metabolic phenotype and polarization state of microglia, reduced the production of inflammatory factors by binding to CISD1, and thereby exerted antidepressant effects.

In addition, our study suggests that CISD1 is an important target for pioglitazone to exert antidepressant effects, but several studies suggested that peroxisome proliferator‐activated receptor gamma (PPAR‐γ) also play an important role.^[^ ^35^, ^80^, ^81^, ^82^, ^83^ ^]^ For example, pioglitazone prevents sevoflurane‐induced severe neuroinflammation and cognitive decline under chronic intermittent hypoxia conditions by upregulating hippocampal PPAR‑γ.^[^ ^84^ ^]^ Pioglitazone can reduce mechanical allodynia pain through activating PPAR‑γ.^[^ ^85^ ^]^ Although the present study does not focus on the role of PPAR‐γ, some studies have found that pioglitazone alleviates mitochondrial dysfunction and provides neuroprotection in wild‐type mice and these ameliorating effects are absent in CISD1 null mice.^[^ ^86^ ^]^ Our data suggest that pioglitazone alleviate stress susceptibility caused by CISD1 overexpression in mice. Furthermore, pretreatment with a PPAR‐γ antagonist also failed to block the antidepressant effect of pioglitazone. Therefore, it is reasonable to assume that pioglitazone exerts an antidepressant effect by modulating CISD1.

## 4 Conclusion

In conclusion, this study demonstrated that CISD1‐mediated alterations in the microglial metabolic phenotype contribute to depressive‐like behavior in mice. CISD1 promotes glycolysis by shifting the microglial NAD⁺/NADH ratio, thereby triggering microglial inflammatory activation. In addition, pioglitazone ameliorates depressive‐like behavior in mice by blocking CISD1‐dependent redox reactions. This study not only identifies a reliable target for the treatment of stress‐related depression, but also offers valuable guidance for screening potential therapeutic drugs.

## 5 Experimental Section

### Animals

7‐week‐old male C57BL/6J mice (20–22 g) were obtained from Hunan SJA Laboratory Animal. CD‐1(ICR) mice were purchased from GemPharmatech and Charles River Laboratories, and Cx3cr1‐Cre mice were obtained from The Jackson Laboratory. Mice were randomly assigned to experimental groups and housed under standard conditions: 12‐h light/dark cycle (lights on at 7:00 a.m.); Ambient temperature maintained at 23–25 °C; Ad libitum access to food and water. Unless otherwise stated, male mice were used in all experiments of this study. All animal procedures were approved by the Institutional Animal Care and Use Committee of Hunan University (HNU‐IACUC‐2024‐118) and conducted in compliance with institutional and national guidelines for animal welfare.

### Chronic Social Defeat Stress Model

Male mice were used for chronic social defeat stress (CSDS).^[^ ^87^ ^]^ Animals were randomly assigned to groups based on cage numbers. Each C57BL/6J male mouse was exposed daily to a different aggressive CD‐1 male for 5–10 min over 10 consecutive days. Following each encounter, the C57BL/6J mouse was housed with the CD‐1 aggressor in a divided cage separated by a perforated plexiglass barrier, allowing continuous visual, olfactory, and auditory contact while preventing physical interaction. On day 11, social interaction testing was performed to assess social avoidance. Control C57BL/6J mice were pair‐housed in identical divided cages without exposure to defeat.

### Subthreshold Social Defeat Stress

To induce subthreshold social defeat stress, a modified protocol was applied.^[^ ^24^ ^]^ Mice were randomly assigned to groups by cage number and exposed to three 5‐min defeat sessions, each separated by a 15‐min interval with a different CD‐1 aggressor. After the sessions, mice were singly housed in their home cages. The social interaction test was performed 24 h later.

### Cell Culture and Treatment

Murine BV2 microglial cells (Procell Life Science&Technology, CL‐0493A) were authenticated by the supplier. Cells were maintained in Dulbecco's Modified Eagle Medium (DMEM, Dakewe, 6016121) supplemented with 10% heat‐inactivated fetal bovine serum (FBS, Absin, abs972) and 1% penicillin and streptomycin (NEST Biotechnology, 211092). Cells were grown in an incubator at 37 °C in 5% CO_2_. For stimulation, BV2 cells were primed with LPS (200 ng mL^−1^, 3 h; MCE, HY‑D1056) followed by ATP (3 mM, 30 min; MCE, HY‑B2176). In some experiments, cells were pretreated with pioglitazone (8 µM, 24 h; MCE, HY‑13956) or NL‐1 (10 µM, 12 h; MCE, HY‐135231) before subsequent analyses.

### Social Interaction Test

The social interaction test consisted of two 2.5‐min sessions.^[^ ^24^ ^]^ In the first session, a C57BL/6J mouse was placed in a 45 × 45 cm arena containing an empty 10 × 6 cm wire‐mesh cage. In the second session, an unfamiliar CD‐1 mouse was introduced into the cage, and the C57BL/6J mouse was re‐exposed to the arena. The interaction ratio was defined as the time spent in the interaction zone (14 × 26 cm) during the target phase divided by that in the no‐target phase. Zone boundaries were set as the interaction zone (14 × 26 cm) and corner zone (10 × 10 cm). An interaction ratio < 1.0 indicated susceptibility, whereas a ratio ≥ 1.0 indicated resilience.

### Sucrose Preference Test

The sucrose preference test was conducted as previously described with minor modifications.^[^ ^88^ ^]^ Mice were habituated to two identical 50‐ml bottles containing 1% sucrose solution or water for 48 h, with bottle positions switched after 24 h to avoid side bias. After 12 h of fluid deprivation, sucrose and water intake were measured during a 2‐h test. Sucrose preference (%) was calculated as 100 × (sucrose intake / total fluid intake).

### Selection Criteria for Mice with Depressive‐Like Behavior

Based on previous studies, depressive‐like phenotypes after 10 days of CSDS were assessed by social avoidance and anhedonia. Mice with a social interaction ratio < 1.0 and sucrose preference < 75% were classified as susceptible, whereas those with a ratio ≥ 1.0 and preference ≥ 75% were classified as resilient. Animals not meeting both criteria were excluded. Control mice were defined as those with a social interaction ratio ≥ 1.0 and sucrose preference ≥ 75%.

### Forced Swim Test

The forced swim test was used to evaluate depressive‐like behavior by measuring immobility time.^[^ ^89^ ^]^ Mice were placed individually in a transparent glass cylinder (35 cm high, 15 cm in diameter) filled with 10 cm of water maintained at 25 ± 1 °C. Water was replaced after each trial. Each session lasted 6 min, and immobility was recorded during the final 5 min. Immobility was defined as the absence of active escape behaviors, with only minimal movements required to keep the head above water.

### Tail Suspension Test

The tail suspension test was regarded as a reflection of despair or depressive‐like behavior based on the immobility time.^[^ ^90^ ^]^ Mice were suspended 20 cm above the ground with adhesive tape placed 1 cm from the tail tip. Immobility was defined as the absence of body movement and passive hanging for at least 10 s. The total immobility duration was recorded over a 6‐min test.

### Open‐Field Test

The open‐field test was conducted in a rectangular opaque white plastic chamber (50 × 50 × 40 cm) to evaluate locomotor activity and anxiety‐like behavior.^[^ ^91^ ^]^ The floor was divided into a central zone (25 × 25 cm) and a surrounding border zone. Mice were placed in the center and allowed to explore freely for 10 min. Behavioral activity was recorded using a video camera positioned 180 cm above the apparatus and analyzed with VisuTrack software (XR‐VT, Shanghai Xinruan, China).

### Fluorescence‐Activated Cell Sorting

On the second day after behavioral testing, both CSDS and control mice were euthanized, and the mPFC was dissected. The tissues were digested with collagenase I (MCE, HY‐O0004) to obtain a single‐cell suspension, which was then incubated with fluorescent antibodies against CD11b, CD45, MAP2, and ACSA‐2. Cells were subsequently sorted by flow cytometry into three populations: CD11b⁺CD45^−^ microglia, MAP2⁺ neurons, and ACSA‐2⁺ astrocytes. All procedures were performed at 4 °C.

### Microglia Isolation from Mice Brain

Microglia were isolated from adult mouse brains following established protocols with minor modifications.^[^ ^92^, ^93^ ^]^ Briefly, 3‐month‐old C57BL/6J mice were anesthetized by intraperitoneal injection of sodium pentobarbital (40 mg kg^−1^) and transcardially perfused with ice‐cold saline containing actinomycin D (5 mg mL^−1^; MCE, HY‐17559) and triptolide (10 mM; MCE, HY‐32735) to minimize microglial activation. The medial prefrontal cortex (mPFC) was rapidly dissected, minced, and suspended in Hanks’ Balanced Salt Solution (YEASEN, 60147ES76) supplemented with HEPES (1.5 mM), glucose (0.5%), actinomycin D (5 mg mL^−1^), triptolide (10 mM), and anisomycin (27.1 mg mL^−1^; MCE, HY‐18982). Tissue suspensions were homogenized using a Dounce homogenizer, passed through a 70 µm strainer, and centrifuged at 600 g for 6 min. Cell pellets were resuspended in 37% Percoll and overlaid between 30% and 70% Percoll gradients prepared from a 100% stock (Cytiva, 17 089102). After density‐gradient centrifugation, microglia were collected from the 37%–70% interphase, washed with a tenfold volume of PBS (Procell, PB180327), and centrifuged again at 600 g for 6 min. All steps were performed at 4 °C under light‐protected conditions.

### Immunofluorescent Staining

Mice were intracardially perfused with 0.9% saline, followed by 4% paraformaldehyde in PBS. Brains were post‐fixed in 4% paraformaldehyde for 16 h and subsequently placed in a 10–30% sucrose solution at 4 °C for 2 days. Fixed brain tissues were cut into 30‐µm‐thick sections using a freezing microtome (CM1900, Leica, Germany). Sections were incubated overnight at 4 °C with primary antibodies: anti‐Iba1 (1:300, Abcam, ab5076) and anti‐CD68 (1:200, TargetMol, TMAB‐00383), followed by Alexa dye–conjugated secondary antibodies. After three washes, nuclei were counterstained with DAPI (Coolaber, SL7101) for 10 min at room temperature. Fluorescence images were acquired using a Zeiss LSM 980 confocal microscope (Carl Zeiss, Germany) and analyzed with ImageJ software.

### Cytokine Analysis

After isolating fresh the mPFC tissues or extracting microglia by gradient separation, the levels of inflammatory factors IL‐1β (R&D Systems, DY401), IL‐6 (R&D Systems, DY406) and TNF‐α (R&D Systems, DY410) was measured using a commercial kit.

### Quantification of Lactate

The lactate concentration was determined by an enzymatic reaction which results in a colorimetric (570 nm) product when lactate is converted to pyruvate using the lactate assay kit (Solarbio, BC2235). Briefly, the mPFC tissue (≈5 mg) was acutely micro‐dissected after mice were anesthetized and incubated in the 1.5 mL EP tubes with 80 µL of ice‐cold oxygenized ACSF for 12 min. The ACSF was then collected and subjected to lactate measurement according to the manufacturer's instructions. All experiments were carried out in duplicate. Absorbance was measured at 570 nm using a microplate reader (BioTek, USA).

### Quantification of PDH and LDH

The PDH (Solarbio, BC0385) and LDH (Abbkine, KTB1110) activity was determined using a coupled enzyme reaction, which results in a colorimetric product proportional to the enzymatic activity. Briefly, the microglia from the mPFC add to wells, then PDH and LDH detection working liquid was added to each well, The 96‐well plate was placed on a shaker at room temperature away from light for 30 min. Finally, the absorbance at 605 nm or 450 nm was determined by a microplate reader.

### Reactive Oxygen Species

The ROS in the mPFC neurons was evaluated using the DHE (MCE, HY‐D0079). Brain slices were incubated with a 5 µM working solution of DHE for 10 min at 37 °C, followed by two careful washes with PBS. Fluorescence images were acquired using a Zeiss LSM 980 confocal microscope and analyzed with ImageJ software.

### Quantification of NAD^+^/NADH

The NAD⁺/NADH ratio was measured in lysates of mPFC tissue or primary microglia using a commercial assay kit (NAD^+^/NADH Assay Kit, Absin, abs90116) according to the manufacturer's instructions.

### Quantification of GAPDH

The enzymatic activity of GAPDH in mPFC tissue lysates was determined using a commercially available Glyceraldehyde‐3‐Phosphate Dehydrogenase Activity Assay Kit (Abcam, ab204732), following the manufacturer's protocol.

### Quantification of Complex I and Complex II

The activities of mitochondrial complexes I and II were measured in lysates of mPFC tissue or primary microglia using a Complex I Activity Assay Kit (Solarbio, BC0515) and a Complex II Activity Assay Kit (Solarbio, BC3230), respectively, following the manufacturer's instructions.

### Stereotactic Surgeries

For local knockdown of CISD1, GFP‐tagged lentiviral vectors (LV‐U6‐shRNA‐*Cisd1*‐CMV‐GFP, 7 × 10^8^ TU mL^−1^, LV‐sh*Cisd1*) expressing shRNA against CISD1 (5′‐GGCGTAGGACCTCTGATCATCAACTCGAGTTGATGATCAGAGGTCCTACGTTTTTTG‐3′) were stereotaxically injected into the mPFC of adult mice (1.0 µL per side; AP = + 1.9 mm, ML = ± 0.4 mm, DV = − 2.18 mm). Microglia‐specific CISD1 knockout was achieved by injecting AAV‐CMV‐DIO‐GFP‐miRNA30shRNA(*Cisd1*)‐WPRE (MG1.2, 2.1 × 10^13^ vg mL^−1^, AAV‐sh*Cisd1*) into the mPFC of Cx3cr1‐Cre mice (0.3 µL per side). For local overexpression of CISD1, a lentiviral vector construct containing GV358 (Ubi‐MCS‐CMV‐EGFP) encoding CISD1 (6.5 × 10^8^ TU mL^−1^, LV‐*Cisd1*) was used. Mice were housed individually and allowed to recover for at least 7 days after surgery. AAV‐sh*Cisd1* was obtained from Obio Technology (Shanghai, China), whereas LV‐sh*Cisd1* and LV‐*Cisd1* were purchased from Genechem (Shanghai, China).

### Animal Drug Administration

Pioglitazone treatment, pioglitazone was dissolved in saline and administered to mice by intragastric gavage at 1, 2, 4, or 6 mg/kg^−1^ once daily for three consecutive days. NL‑1 treatment, mice were anesthetized with sodium pentobarbital (40 mg kg^−1^, i.p.) and secured in a stereotaxic frame for bilateral guide‐cannula implantation into the mPFC. After a 7‐day recovery period, NL‐1 (50 µM, 2 µL per side) was microinjected into the mPFC, followed by behavioral testing. T0070907 treatment, mice were implanted with bilateral guide cannulas into the mPFC, then T0070907 (MCE, HY‐13202; 10 µM, 1 µL per side) was microinjected into the mPFC, followed by intragastric administration of pioglitazone 6 h later. Behavioral tests were performed 3 days later.

### Western Blotting

The mPFC tissue and microglia were homogenized on ice for 5 min using a no‐touch ultrasonic homogenizer (SCIENTZ08 IIIC, SCIENTZ, China) in RIPA buffer containing a protease inhibitor cocktail (Abbkine, BMP1001). Following sonication, samples were centrifuged at 12 000 g for 20 min at 4 °C, and the supernatant was collected and quantified using a BCA assay (YEASEN, 20201ES). Protein samples were heated at 95 °C for 10 min in loading buffer, separated by SDS–PAGE, and transferred to nitrocellulose membranes. Membranes were blocked with 5% non‐fat milk in Tris‐buffered saline containing 0.1% Tween‐20 for 2 h at room temperature, then incubated overnight at 4 °C with primary antibodies: anti‐CISD1 (1:1000, Proteintech, 68030‐1‐lg), anti‐Iba1 (1:1000, Dakewe, 8611903; Bioss, bs‐1363R), and anti‐beta Actin (1:3000, New Cell & Molecular Biotech, AB1100). Membranes were subsequently incubated with peroxidase‐conjugated secondary antibody (1:5000, Proteintech, SA00001‐1) at room temperature for 90 min. Protein signals were detected using chemiluminescence (Sparkjade ECL Plus, Shandong Sparkjade Biotechnology Co., Ltd., ED0016‐C) and quantified with ImageJ software.

### Quantitative Real‐Time PCR

Total RNA was extracted from tissues using TRIzol reagent (Invitrogen, 15596018CN) following the manufacturer's instructions. Reverse transcription was performed with ABScript Neo RT Master Mix for qPCR with gDNA Remover (ABclonal, RK20433). Quantitative real‐time PCR was carried out using 2× Universal SYBR Green Fast qPCR Mix (ABclonal, RK21203). GAPDH mRNA quantification was used as a loading control for normalization. Relative mRNA expression was calculated using the 2^‐ΔΔCT^ method. Each reaction was performed in triplicate. Primer sequences (Tsingke, China) are listed in Table S1, Supporting Information.

### Measurement of Mitochondrial Respiration and Glycolysis

The XF Cell Mito Stress Test kit and XF Glycolysis Stress Test Kit (Agilent, 103015‐100 and 103020‐100) were used to measure mitochondrial oxygen consumption rate (OCR) and glycolytic activity following the manufacturer's instructions.^[^ ^94^, ^95^ ^]^ BV2 or primary microglial cells were seeded at 2 × 10⁴ cells per well in XFe24 microplates. Prior to the assay, cells were incubated in assay medium supplemented with 10 mM pyruvate and malate, and the plates were placed in a CO_2_‐free incubator at 37 °C for 1 h. Plates were then transferred to the XFe24 Analyzer. For the mitochondrial stress test, oligomycin (1.0 µM), carbonyl cyanide‐4‐(trifluoromethoxy) phenylhydrazone (FCCP, 1.0 µM), and a mixture of rotenone and antimycin A (0.5 µM each) were sequentially injected to assess basal respiration, ATP production, maximal respiration, and spare respiratory capacity. For the glycolysis test, glucose (1 µM), oligomycin (0.1 µM), and 2‐deoxy‐D‐glucose (2‐DG, 1 µM) were added sequentially to evaluate glycolytic function.

### Statistical Analysis

All data collection and statistical analyses were performed blind, and no data were excluded. Sample size estimation was conducted using G*Power software (version 3.1). Data are presented as the mean ± SEM. P < 0.05 was considered statistically significant. The statistical details can be found in Table S2, Supporting Information. All experiments were conducted in triplicate in independent runs. The number of biological replicates for each experiment was determined based on prior experience in our laboratory and information reported in related literature. For comparisons between two groups, data were first tested for normality using the Shapiro–Wilk test. For multiple group comparisons, one‑way or two‑way ANOVA followed by Bonferroni's post hoc test was used as appropriate. Statistical analyses were carried out using GraphPad Prism v.8 software (GraphPad, USA).

## Conflict of Interest

The authors declare no conflict of interest.

## Author Contributions

W.D. designed experiments, performed data analyses and wrote the manuscript. W.D. and S.H. performed the animal experiments, W.D., D.L., and S.F. performed the cell experiments. J.Z. and X.C. provided some reagents. S.H. conceived the study and revised the manuscript with the help of author W.D.

## Supporting information



Supporting Information

## Data Availability

The data that support the findings of this study are available from the corresponding author upon reasonable request.
