# Supplementary material for: Mitochondrial CISD1 Modulates Microglial Metabolic Reprogramming to Drive Stress Susceptibility in Mice
Source: Adv Sci (Weinh). 2025 Nov 11;13(6):e08957. doi: 10.1002/advs.202508957 (PMC12866790; doi:10.1002/advs.202508957)
Supplement: Supplementary file 1 — Supporting Information [file ADVS-13-e08957-s001.docx]

Supporting Information

Mitochondrial CISD1 Modulates Microglial Metabolic Reprogramming to Drive Stress Susceptibility in Mice

*Wanting Dong*, Duo Liu, Songsen Fu, Jiaming Zhang, Xi Chen, and Songqiang Huang**

W. Dong, D. Liu, S. Fu, S. Huang

Affiliated Hospital of Hunan University, School of Biomedical Sciences

Hunan University

Changsha 410082, China

E-mail: [dongwanting2024@hnu.edu.cn](mailto:Dongwanting2024@hnu.edu.cn); [huangsq133@hnu.edu.cn](mailto:Huangsq133@hnu.edu.cn)

J. Zhang

Clinical Innovation & Research Center (CIRC), Shenzhen Hospital

Southern Medical University

Shenzhen 518100, China

X. Chen

Department of Pharmacy, Traditional Chinese and Western Medicine Hospital of Wuhan

Tongji Medical College

Huazhong University of Science and Technology

Wuhan 430033, China

**Supplementary figures**


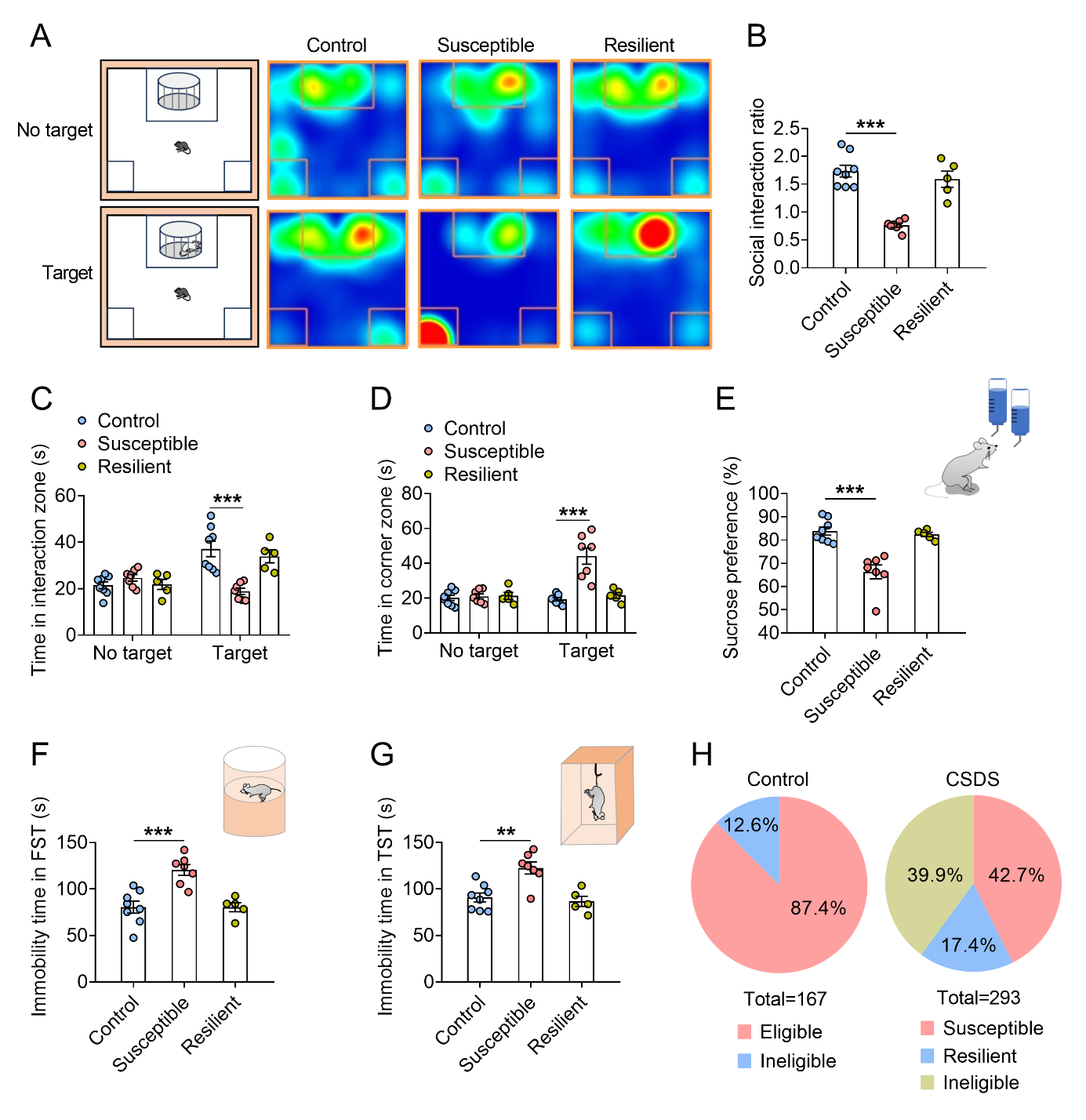


**Figure S1.** Chronic social defeat stress induces the depressive-like behavior in mice. A-E) Schematic and typical heat plot of mice in social interaction test (A), social interaction ratio (B), time in interaction zone (C), time in corner zone (D), percentage of sucrose preference (E) of mice from control, susceptible and resilient groups (n = 5-8). F, G) Forced swim test (FST, F) and tail suspension test (TST, G) show that immobility time of control, susceptible and resilient groups (n = 5-8). H) The pie chart displays the eligible rate in control mice and the proportions of susceptible versus resilient mice in the chronic social defeat stress (CSDS) group. Data are presented as the mean ± SEM. Statistical analysis by one-way ANOVA with Bonferroni’s post hoc test in (B, E-G) or two-way ANOVA with Bonferroni’s post hoc test in (C, D). **p < 0.01, ***p < 0.001.


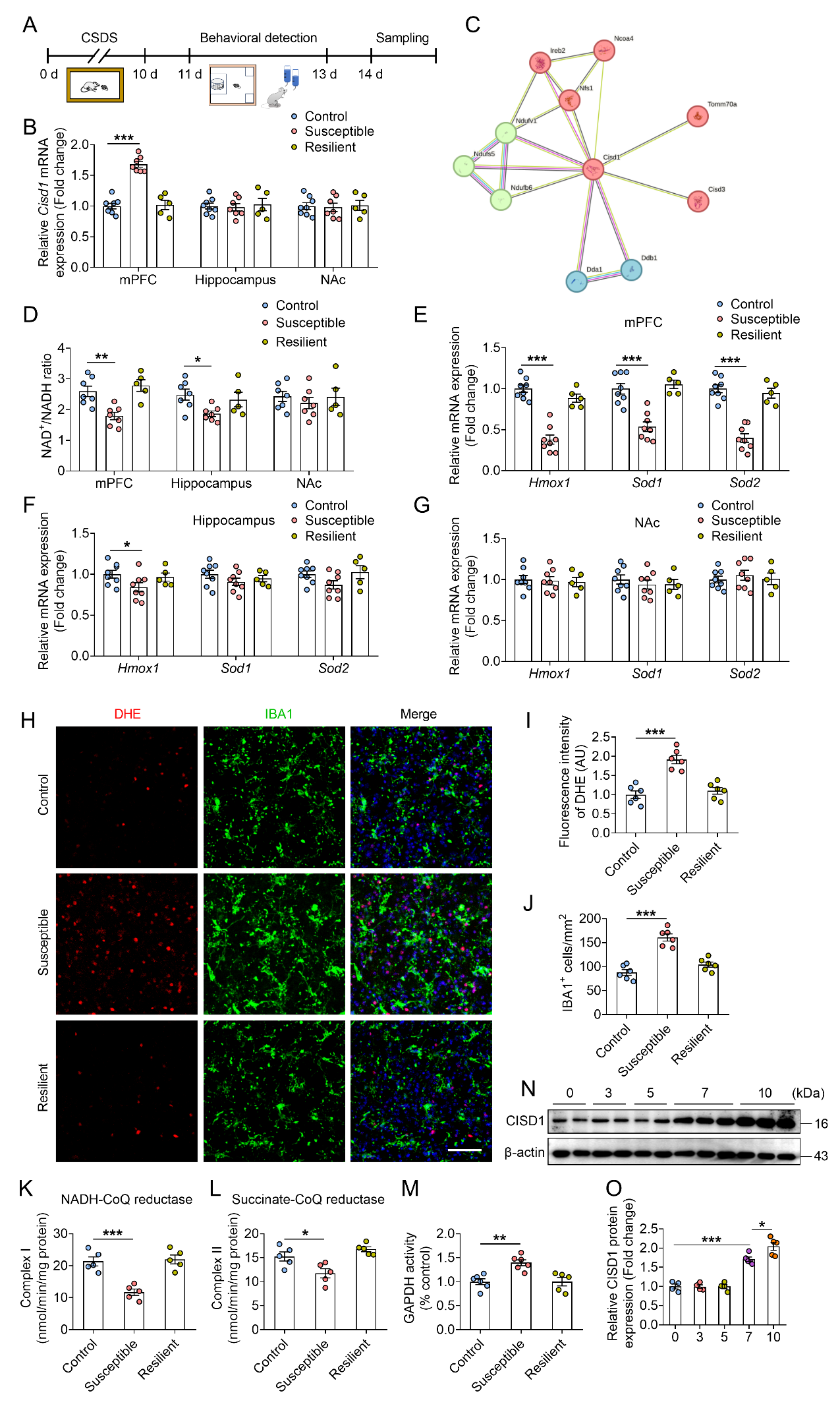


**Figure S2.** Chronic social defeat stress increases the mRNA expression of *Cisd1* in the medial prefrontal cortex of susceptible mice. A) Experimental timeline of animal treatment. B) The mRNA expression of *Cisd1* in the medial prefrontal cortex (mPFC), hippocampus and nucleus accumbens (NAc) from control, susceptible and resilient mice (n = 5-8). C) The String analysis predicted the interaction network between CISD1 and other proteins (pink ball, redox related protein; cyan ball, mitochondrial complex I functional protein). D) NAD^+^/NADH ratio in the mPFC, hippocampus and NAc from control, susceptible and resilient mice (n = 5-7). E-G) The mRNA expression of antioxidant genes heme oxygenase-1 (*Hmox1*), superoxide dismutase 1 (*Sod1*) and *Sod2* in the mPFC (E), hippocampus (F) and NAc (G) from control, susceptible and resilient mice (n = 5-8). H-J) Representative immunofluorescence images (H) and group data (I, J) showing DHE (ROS marker, red) co-staining with IBA1 (microglia marker, green; n = 6). Scale bars indicate 100 µm. K) The activity of complex I in the mPFC from control, susceptible and resilient mice (n = 5). L) The activity of complex II in the mPFC from control, susceptible and resilient mice (n = 5). M) The activity of glyceraldehyde-3-phosphate dehydrogenase (GAPDH) in the mPFC from control, susceptible and resilient mice (n = 5-6). N, O) Representative western blots (N) and analyses (O) of CISD1 in the mPFC of mice at different time points (n = 4-5). Data are presented as the mean ± SEM. Statistical analysis by one-way ANOVA with Bonferroni’s post hoc test in (I-M, O) or two-way ANOVA with Bonferroni’s post hoc test in (B, D-G). *p < 0.05, **p < 0.01, ***p < 0.001.


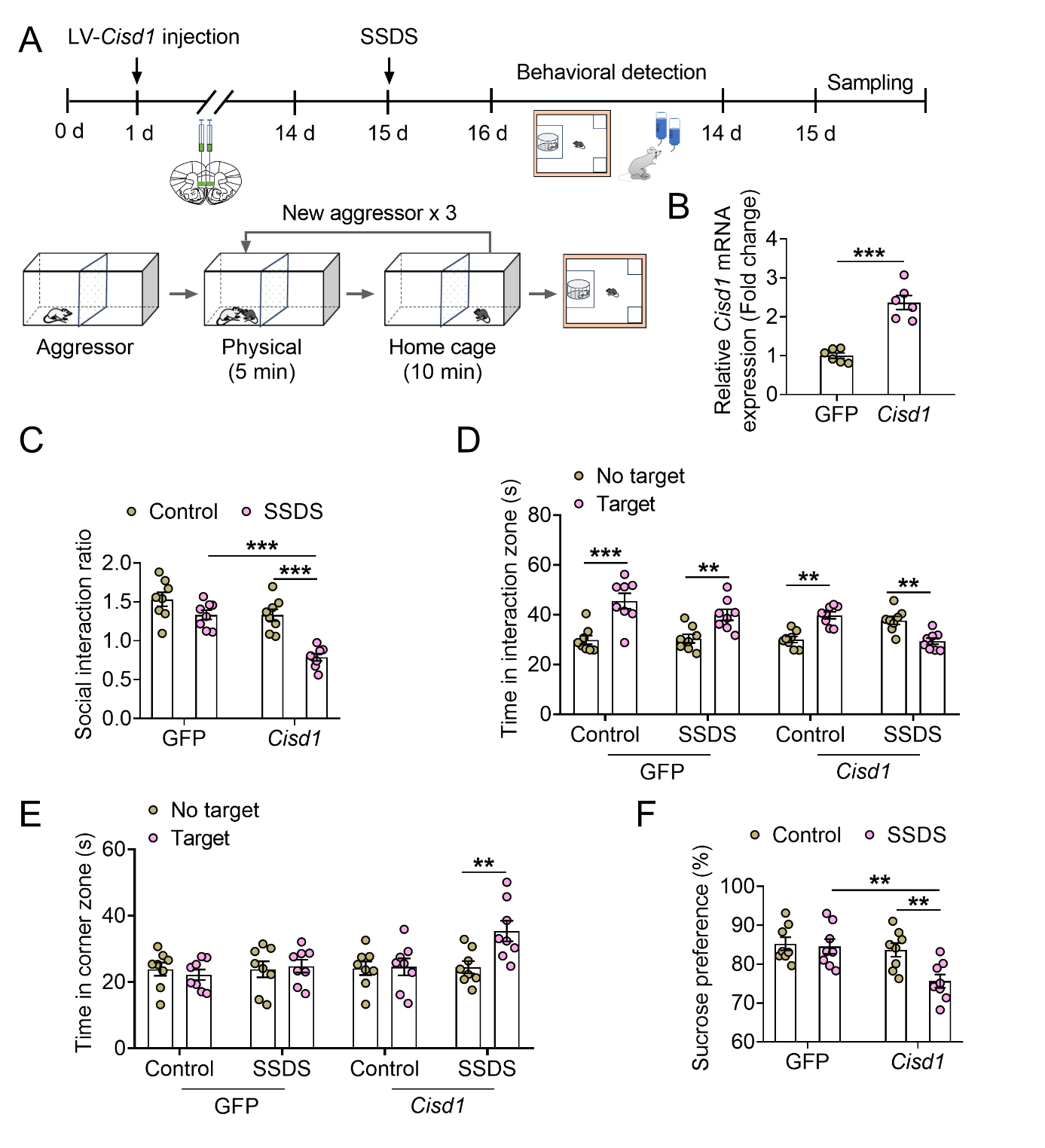


**Figure S3.** Overexpression of CISD1 increases stress susceptibility in mice. A) The experimental timeline shows that, after injection of the CISD1 overexpression virus (LV-*Cisd1*) into the medial prefrontal cortex (mPFC) of mice, subthreshold social defeat stress (SSDS) was performed and behavioral testing was subsequently conducted. B) The mRNA expression of *Cisd1* from GFP and *Cisd1* groups (n = 6). C-F) The social interaction ratio (C), time in interaction zone (D), time in corner zone (E) and percentage of sucrose preference (F) of mice from control-GFP, SSDS-GFP, control-*Cisd1* and SSDS-*Cisd1* groups (n = 8). Data are presented as the mean ± SEM. Statistical analysis by unpaired Student’s t test in (B) or two-way ANOVA with Bonferroni’s post hoc test in (C-F). **p < 0.01, ***p < 0.001.


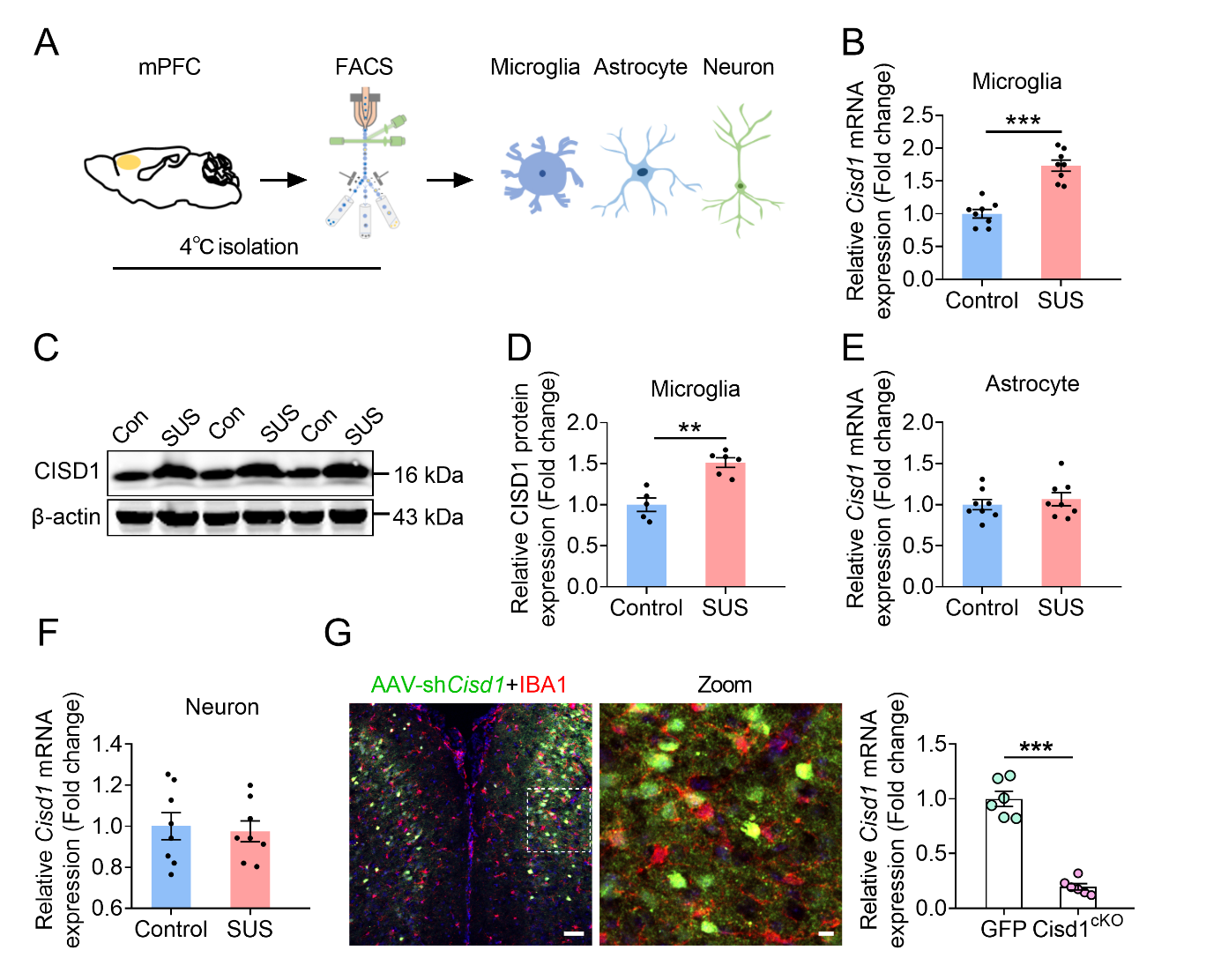


**Figure S4**. Chronic stress increases microglial CISD1 expression in the medial prefrontal cortex of mice. A) Schematic diagram shows the fluorescence-activated cell sorting (FACS) used to isolate distinct cell populations from the medial prefrontal cortex (mPFC). B) The mRNA expression of *Cisd1* in microglia from control and susceptible (SUS) mice (n = 8). C, D) Representative western blots (C) and group data (D) shows the protein expression of CISD1 in microglia from control and SUS mice (n = 5-6). E, F) The mRNA expression of *Cisd1* in astrocytes (E) and neurons (F) from control and SUS mice (n = 8). G) Representative immunofluorescence images show that the microglia-targeting virus AAV-CMV-DIO-GFP-miRNA30shRNA(*Cisd1*)-WPRE (AAV-sh*Cisd1*) was microinjected into the mPFC of Cx3cr1-Cre mice to generate microglia-specific CISD1 knockout (Cisd1^cKO^) mice, and knockout efficiency was comfirmed by measuring *Cisd1* mRNA expression in GFP and Cisd1^cKO^ mice (n = 6). Scale bars indicate 50 µm (left) and 10 µm (right). Data are presented as the mean ± SEM. Statistical analysis by unpaired Student’s t test in (B, D-G). **p < 0.01, ***p < 0.001.


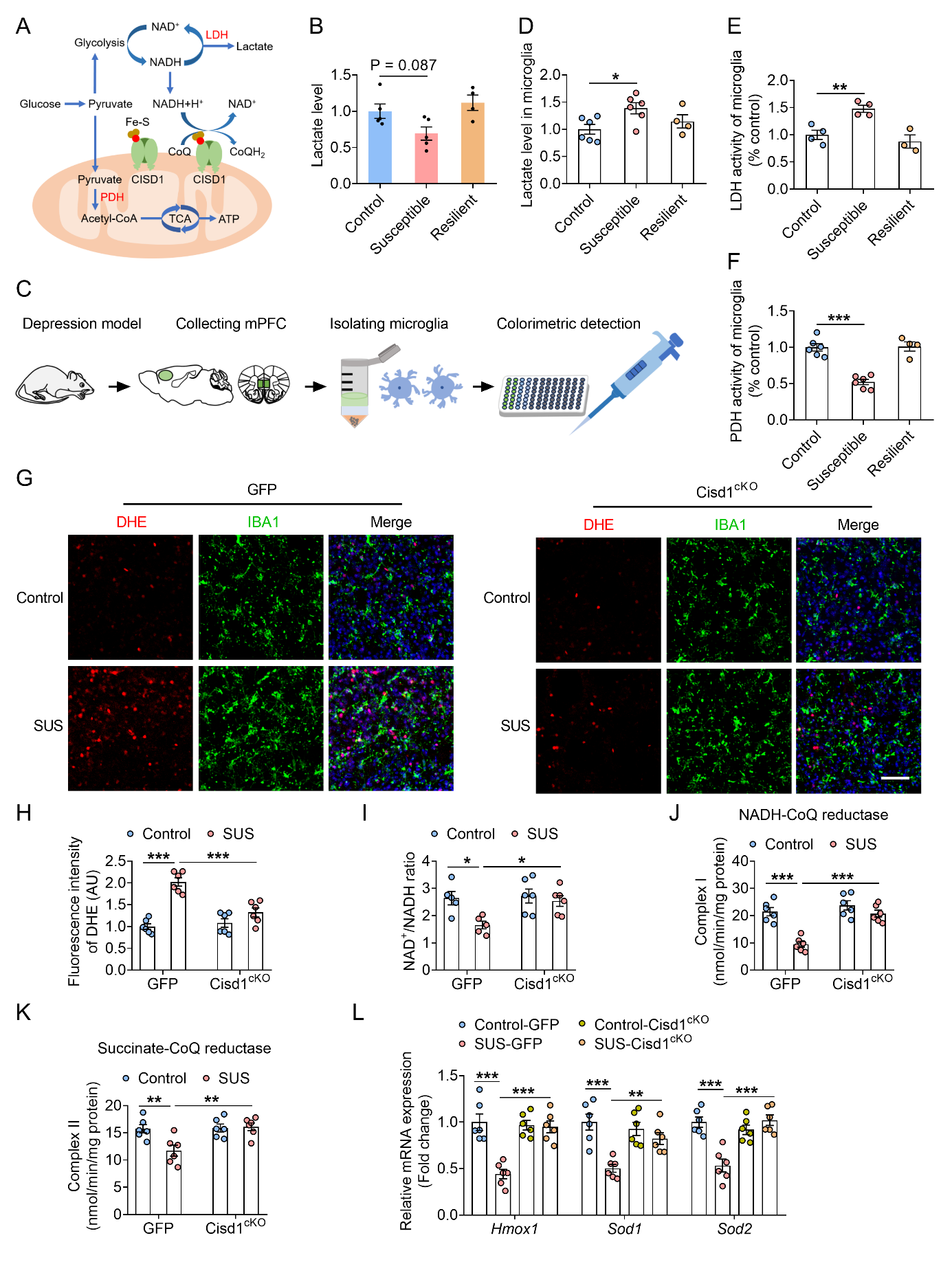


**Figure S5**. Chronic stress increases microglial lactate levels in the medial prefrontal cortex of mice. A) Schematic diagram illustrates how the Fe–S cluster of CISD1 enhances the conversion of glucose to lactate by promoting NADH depletion and concurrently reduces pyruvate entry into the tricarboxylic acid cycle. B) Lactate levels in the medial prefrontal cortex (mPFC) from control, susceptible and resilient mice (n = 4-5). C) Schematic diagram of the experimental process shows that microglia were isolated from the mPFC of mice and subsequently analyzed by colorimetric assay. D) Lactate levels in microglia from control, susceptible and resilient mice (n = 4-6). E, F) Activities of lactate dehydrogenase (LDH, E) and pyruvate dehydrogenase (PDH, F) in microglia from control, susceptible and resilient mice (n = 3-6). G, H) Representative immunofluorescence images (G) and group data (H) showing DHE (ROS marker, red) co-staining with IBA1 (microglia marker, green; n = 6). Scale bars indicate 100 µm. I) NAD^+^/NADH ratio in the mPFC from control-GFP, susceptible (SUS)-GFP, control-microglia-specific CISD1 knockout (Cisd1^cKO^) and SUS-Cisd1^cKO^ mice (n = 5-6). J) The activity of complex I in the mPFC from control-GFP, SUS-GFP, control-Cisd1^cKO^ and SUS-Cisd1^cKO^ mice (n = 6). K) The activity of complex II in the mPFC from control-GFP, SUS-GFP, control-Cisd1^cKO^ and SUS-Cisd1^cKO^ mice (n = 6). L) The mRNA expression of antioxidant genes heme oxygenase-1 (*Hmox1*), superoxide dismutase 1 (*Sod1*) and *Sod2* in the mPFC from control-GFP, SUS-GFP, control-Cisd1^cKO^ and SUS-Cisd1^cKO^ mice (n = 6). Data are presented as the mean ± SEM. Statistical analysis by one-way ANOVA with Bonferroni’s post hoc test in (B, D-F) or two-way ANOVA with Bonferroni’s post hoc test in (H-L). *p < 0.05, **p < 0.01, ***p < 0.001.


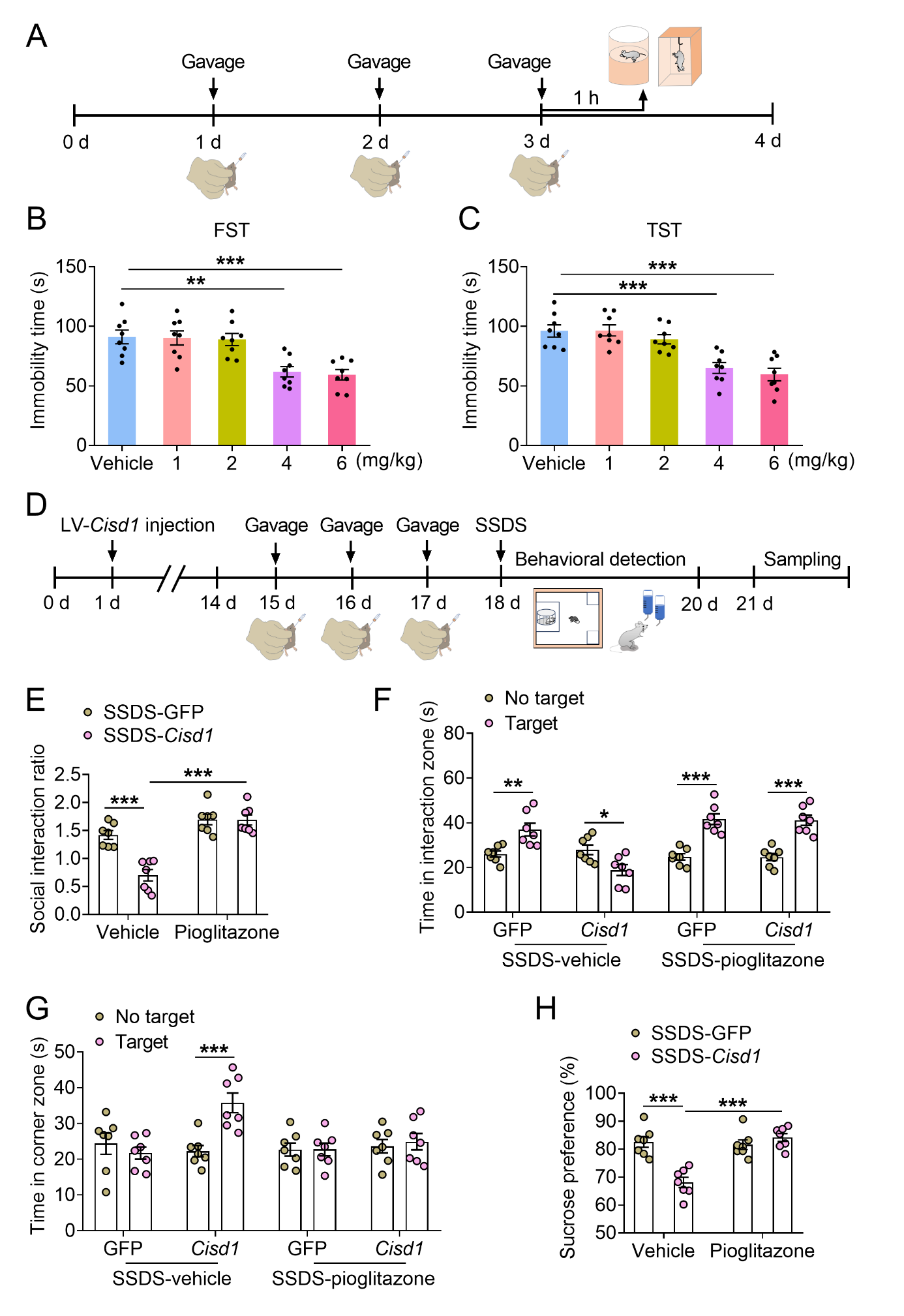


**Figure S6**. Effects of pioglitazone on depressive-like behavior in mice. A) Schematic diagram showing the timing of pioglitazone administration and behavioral testing. B, C) Immobility time in the forced swim test (FST) and tail suspension test (TST) for vehicle‑treated mice and mice receiving different doses of pioglitazone (n = 8). D) The experimental timeline shows that, after injection of the CISD1 overexpression virus (LV-*Cisd1*) into the medial prefrontal cortex of mice, pioglitazone was administered by gavage for 3 consecutive days, followed by subthreshold social defeat stress (SSDS) and then behavioral testing. E-H) The social interaction ratio (E), time in interaction zone (F), time in corner zone (G) and percentage of sucrose preference (H) of mice from SSDS-GFP-vehicle, SSDS*-Cisd1*-vehicle, SSDS-GFP-pioglitazone and SSDS*-Cisd1*-pioglitazone groups (n = 7). Data are presented as the mean ± SEM. Statistical analysis by one-way ANOVA with Bonferroni’s post hoc test in (B, C) or two-way ANOVA with Bonferroni’s post hoc test in (E-H). *p < 0.05, **p < 0.01, ***p < 0.001.


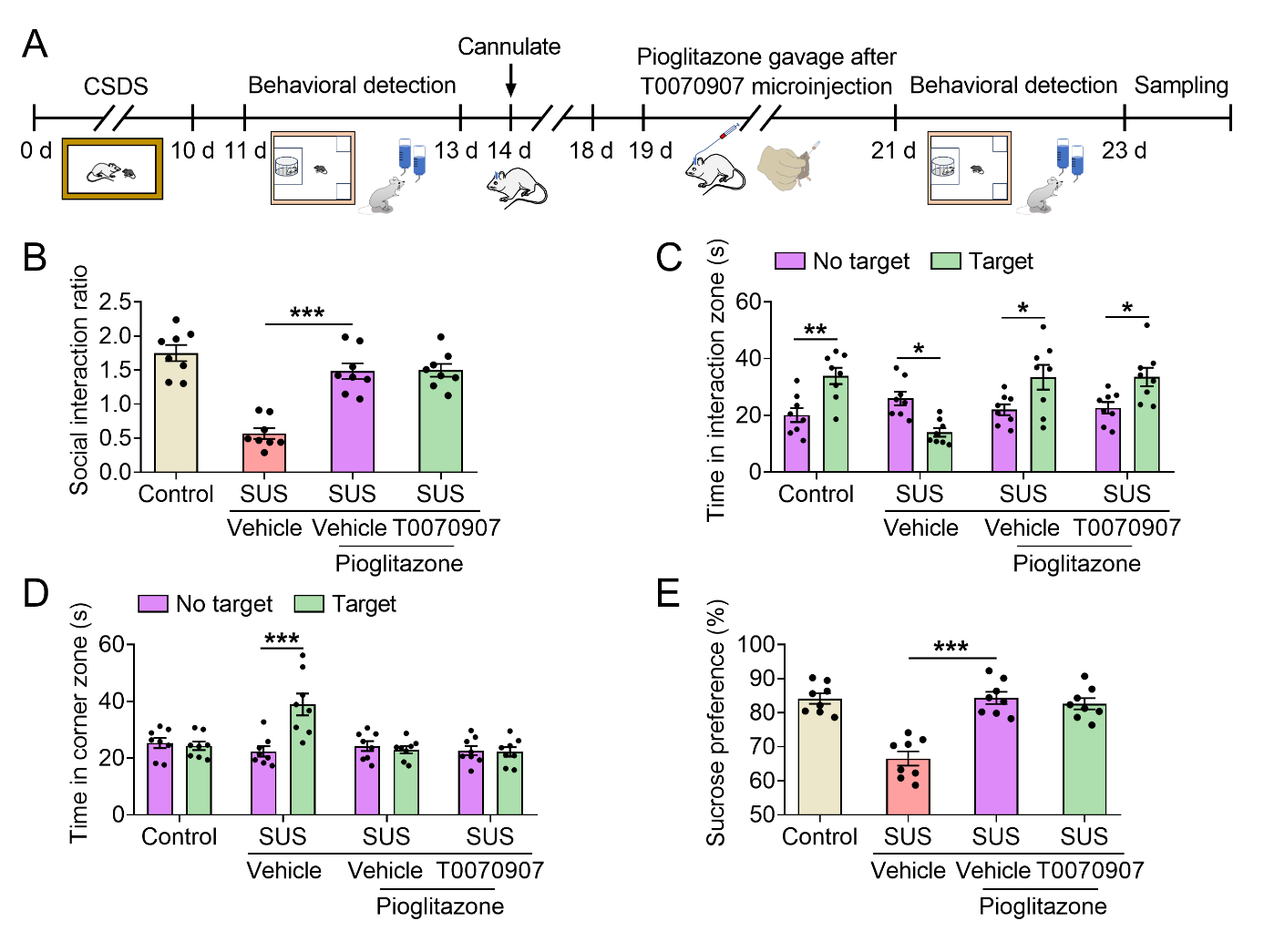


**Figure S7**. PPAR-γ antagonist treatment did not alter the antidepressant effects of pioglitazone in mice. A) Experimental timeline: susceptible (SUS) mice were identified using the CSDS protocol and divided into three groups. Cannulas were bilaterally implanted into the medial prefrontal cortex and allowed to recover for 5 days. From days 19–21, the PPAR-γ inhibitor T0070907 (10 µM, 1 µL/side) was infused daily, followed 6 h later by pioglitazone gavage. Behavioral tests were performed 3 days after treatment. B-E) The social interaction ratio (B), time in interaction zone (C), time in corner zone (D) and percentage of sucrose preference (E) of mice from control, SUS-vehicle, SUS-vehicle-pioglitazone, SUS-T0070907-pioglitazone groups (n = 8). Data are presented as the mean ± SEM. Statistical analysis by one-way ANOVA with Bonferroni’s post hoc test in (B, E) or two-way ANOVA with Bonferroni’s post hoc test in (C, D). *p < 0.05, **p < 0.01, ***p < 0.001.


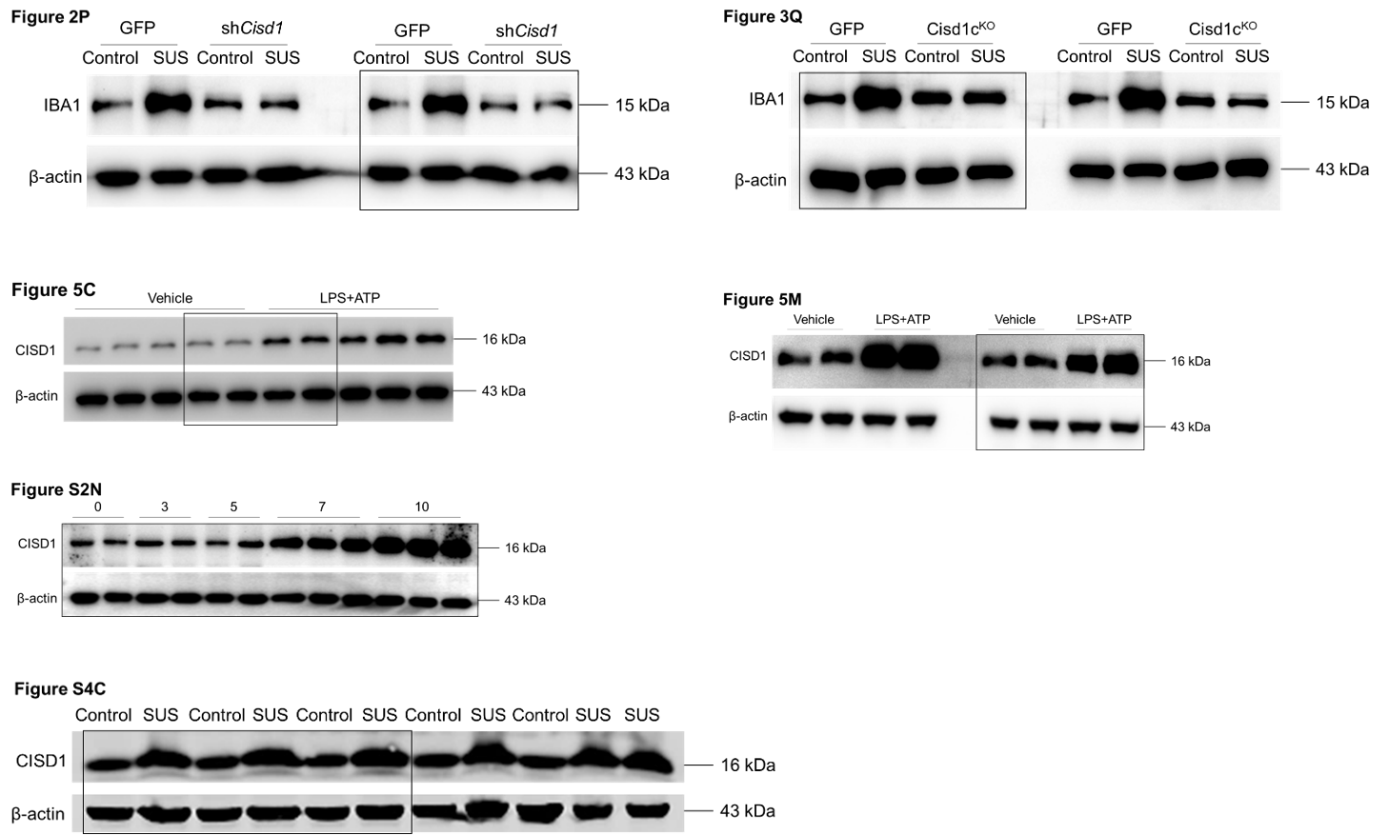


**Figure S8.** Full original images of Western blotting assays for Figure 2P, Figure 3Q, Figure 5C, Figure 5M, Figure S2N and Figure S4C.

**Supplementary Tables**

**Table S1.** Oligonucleotide primers for RT-qPCR

| Names | Sequences (5’ to 3’) |
| --- | --- |
| ***Quantitative RT-PCR*** |  |
| *Cisd1*-F | CAAGGCTATGGTGAATCTTCAG |
| *Cisd1*-R | GTGCCATTCTACGTAAATCAG |
| *Hmox1*-F | CGCCTTCCTGCTCAACATT |
| *Hmox1*-R | TGTGTTCCTCTGTCAGCATCAC |
| *Il1b*-F | TCAGGCAGGCAGTATCACTC |
| *Il1b*-R | CATGAGTCACAGAGGATGGG |
| *Il6*-F | ATCCAGTTGCCTTCTTGGGACTGA |
| *Il6*-R | TAAGCCTCCGACTTGTGAAGTGGT |
| *Sod1*-F | TCTCGTCTTGCTCTCTCTGGT |
| *Sod1*-R | CTGCACTGGTACAGCCTTGTG |
| *Sod2*-F | ATGGTGGGGGACATATT |
| *Sod2*-R | GAACCTTGGACTCCCACAGA |
| *Tnf-a*-F | TTGCTCTGTGAAGGGAATGG |
| *Tnf-a*-R | GGCTCTGAGGAGTAGACAATAAAG |
| *GAPDH*-F | AACGACCCCTTCATTGAC |
| *GAPDH*-R | TCCACGACATACTCAGCAC |

**Table S2.** Statistical analysis for Figures 1-7 and Figures S1-7.

| **Figures and numbers of animals or cells** | **Statistical analysis** | ***Post hoc tests*** | **Mean ± SEM** |
| --- | --- | --- | --- |
| 1D: Social interaction ratio  Control-vehicle (n = 8)  SUS-vehicle (n = 8)  Control-NL-1 (n = 8)  SUS-NL-1 (n = 7) | Two-way ANOVA  NL-1: F (1, 27) = 8.818  Stress: F (1, 27) = 16.650 | Bonferroni's post hoc test  Control-vehicle vs.  SUS-vehicle, *p* < 0.001  SUS-vehicle vs. SUS-NL-1, *p* = 0.003 | Control-vehicle  1.815 ± 0.211  SUS-vehicle  0.626 ± 0.079  Control-NL-1  1.968 ± 0.233  SUS-NL-1  1.604 ± 0.200 |
| 1E: Time in interaction zone (s)  Control-vehicle (n = 8)  SUS-vehicle (n = 8)  Control-NL-1 (n = 8)  SUS-NL-1 (n = 7) | Two-way ANOVA  Stress: F (3, 54) = 8.103  Target: F (1, 54) = 29.770 | Bonferroni's post hoc test  No target vs. Target  Control-vehicle, *p* < 0.001  SUS-vehicle, *p* = 0.025  Control-NL-1, *p* < 0.001  SUS-NL-1, *p* = 0.006 | No target:  Control-vehicle  22.675 ± 1.715  SUS-vehicle  25.438 ± 1.775  Control-NL-1  21.213 ± 1.494  SUS-NL-1  21.829 ± 1.382  Target  Control-vehicle  39.775 ± 3.184  SUS-vehicle  15.525 ± 1.964  Control-NL-1  40.088 ± 3.393  SUS-NL-1  34.343 ± 3.877 |
| 1F: Time in corner zone (s)  Control-vehicle (n = 8)  SUS-vehicle (n = 8)  Control-NL-1 (n = 8)  SUS-NL-1 (n = 7) | Two-way ANOVA  Stress: F (3, 54) = 10.190  Target: F (1, 54) = 9.606 | Bonferroni's post hoc test  No target vs. Target  Control-vehicle, *p*＞0.999  SUS-vehicle, *p* < 0.001  Control-NL-1, *p*＞0.999  SUS-NL-1, *p*＞0.999 | No target:  Control-vehicle  21.900 ± 1.421  SUS-vehicle  22.675 ± 1.173  Control-NL-1  21.663 ± 1.290  SUS-NL-1  22.229 ± 1.104  Target  Control-vehicle  22.263 ± 2.089  SUS-vehicle  37.600 ± 3.384  Control-NL-1  22.013 ± 1.244  SUS-NL-1  22.457 ± 1.192 |
| 1G: Sucrose preference  Control-vehicle (n = 8)  SUS-vehicle (n = 8)  Control-NL-1 (n = 8)  SUS-NL-1 (n = 7) | Two-way ANOVA  NL-1: F (1, 27) = 32.320  Stress: F (1, 27) = 36.840 | Bonferroni's post hoc test  Control-vehicle vs. SUS-vehicle, *p* < 0.001  SUS-vehicle vs. SUS-NL-1, *p* < 0.001 | Control-vehicle  84.784 ± 1.285  SUS-vehicle  68.265 ± 1.779  Control-NL-1  84.721 ± 1.183  SUS-NL-1  84.246 ± 1.203 |
| 1I: Relative *Cisd1* mRNA expression  GFP (n = 8)  sh*Cisd1* (n = 8) | Unpaired *t* test  t = 11.120, df = 14, *p* < 0.001 |  | GFP  1.000 ± 0.055  sh*Cisd1*  0.264 ± 0.037 |
| 1K: Social interaction ratio  Control-GFP (n = 8)  SUS-GFP (n = 8)  Control-sh*Cisd1* (n = 8)  SUS-sh*Cisd1* (n = 8) | Two-way ANOVA  Stress: F (1, 28) = 19.880  Virus: F (1, 28) = 18.800 | Bonferroni's post hoc test  Control-GFP vs. SUS-GFP, *p* < 0.001  SUS-GFP vs. SUS-sh*Cisd1*, *p* < 0.001 | Control-GFP  1.372 ± 0.079  SUS-GFP  0.658 ± 0.080  Control-sh*Cisd1*  1.403 ± 0.112  SUS-sh*Cisd1*  1.361 ± 0.059 |
| 1L: Time in interaction zone (s)  Control-GFP (n = 8)  SUS-GFP (n = 8)  Control-sh*Cisd1* (n = 8)  SUS-sh*Cisd1* (n = 8) | Two-way ANOVA  Stress: F (3, 56) = 6.982  Target: F (1, 56) = 7.694 | Bonferroni's post hoc test  No target vs. Target  Control-GFP, *p* = 0.019  SUS-GFP, *p* = 0.017  Control-sh*Cisd1*, *p* = 0.021  SUS-sh*Cisd1*, *p* = 0.040 | No target  Control-GFP  26.750 ± 2.110  SUS-GFP  26.350 ± 2.128  Control-sh*Cisd1*  24.538 ± 1.722  SUS-sh*Cisd1*  23.650 ± 1.713  Target  Control-GFP  36.100 ± 2.825  SUS-GFP  16.888 ± 2.009  Control-sh*Cisd1*  33.800 ± 2.560  SUS-sh*Cisd1*  32.138 ± 2.642 |
| 1M: Time in corner zone (s)  Control-GFP (n = 8)  SUS-GFP (n = 8)  Control-sh*Cisd1* (n = 8)  SUS-sh*Cisd1* (n = 8) | Two-way ANOVA  Stress: F (3, 56) = 6.919  Target: F (1, 56) = 7.956 | Bonferroni's post hoc test  No target vs. Target  Control-GFP, *p*＞0.999  SUS-GFP, *p* < 0.001  Control-sh*Cisd1*, *p*＞0.999  SUS-sh*Cisd1*, *p*＞0.999 | No target  Control-GFP  23.288 ± 1.477  SUS-GFP  25.625 ± 1.869  Control-sh*Cisd1*  22.763 ± 1.940  SUS-sh*Cisd1*  26.688 ± 1.418  Target  Control-GFP  26.125 ± 2.638  SUS-GFP  37.238 ± 2.216  Control-sh*Cisd1*  24.688 ± 1.496  SUS-sh*Cisd1*  25.088 ± 1.371 |
| 1N: Sucrose preference  Control-GFP (n = 8)  SUS-GFP (n = 8)  Control-sh*Cisd1* (n = 8)  SUS-sh*Cisd1* (n = 8) | Two-way ANOVA  Stress: F (1, 28) = 17.44  Virus: F (1, 28) = 25.52 | Bonferroni's post hoc test  Control-GFP vs. SUS-GFP, *p* < 0.001  SUS-GFP vs. SUS-sh*Cisd1*, *p* < 0.001 | Control-GFP  83.394 ± 1.174  SUS-GFP  72.434 ± 2.078  Control-sh*Cisd1*  85.339 ± 0.969  SUS-sh*Cisd1*  84.619 ± 1.090 |
| 2A: IL-1β (pg/ml)  Control-GFP (n = 6)  SUS-GFP (n = 6)  Control-sh*Cisd1* (n = 6)  SUS-sh*Cisd1* (n = 6) | Two-way ANOVA  Stress: F (1, 20) = 52.520  Virus: F (1, 20) = 37.160 | Bonferroni's post hoc test  Control-GFP vs. SUS-GFP, *p* < 0.001  SUS-GFP vs. SUS-sh*Cisd1*, *p* < 0.001 | Control-GFP  116.168 ± 8.414  SUS-GFP  217.488 ± 10.814  Control-sh*Cisd1*  108.604 ± 6.656  SUS-sh*Cisd1*  125.559 ± 5.859 |
| 2B: IL-6 (pg/ml)  Control-GFP (n = 6)  SUS-GFP (n = 6)  Control-sh*Cisd1* (n = 6)  SUS-sh*Cisd1* (n = 6) | Two-way ANOVA  Stress: F (1, 20) = 106.200  Virus: F (1, 20) = 73.730 | Bonferroni's post hoc test  Control-GFP vs. SUS-GFP, *p* < 0.001  SUS-GFP vs. SUS-sh*Cisd1*, *p* < 0.001 | Control-GFP  24.696 ± 2.194  SUS-GFP  90.041 ± 5.077  Control-sh*Cisd1*  24.493 ± 2.760  SUS-sh*Cisd1*  30.663 ± 3.153 |
| 2C: TNF-α (pg/ml)  Control-GFP (n = 6)  SUS-GFP (n = 6)  Control-sh*Cisd1* (n = 6)  SUS-sh*Cisd1* (n = 6) | Two-way ANOVA  Stress: F (1, 20) = 344.700  Virus: F (1, 20) = 336.200 | Bonferroni's post hoc test  Control-GFP vs. SUS-GFP, *p* < 0.001  SUS-GFP vs. SUS-sh*Cisd1*, *p* < 0.001 | Control-GFP  42.615 ± 5.534  SUS-GFP  211.277 ± 6.575  Control-sh*Cisd1*  32.422 ± 3.485  SUS-sh*Cisd1*  43.735 ± 2.822 |
| 2D: Relative *Il1b* mRNA expression  Control-GFP (n = 8)  SUS-GFP (n = 8)  Control-sh*Cisd1* (n = 8)  SUS-sh*Cisd1* (n = 8) | Two-way ANOVA  Stress: F (1, 28) = 212.000  Virus: F (1, 28) = 203.000 | Bonferroni's post hoc test  Control-GFP vs. SUS-GFP, *p* < 0.001  SUS-GFP vs. SUS-sh*Cisd1*, *p* < 0.001 | Control-GFP  1.000 ± 0.089  SUS-GFP  5.520 ± 0.256  Control-sh*Cisd1*  1.135 ± 0.097  SUS-sh*Cisd1*  1.048 ± 0.099 |
| 2E: Relative *Il6* mRNA expression  Control-GFP (n = 8)  SUS-GFP (n = 8)  Control-sh*Cisd1* (n = 8)  SUS-sh*Cisd1* (n = 8) | Two-way ANOVA  Stress: F (1, 28) = 59.580  Virus: F (1, 28) = 57.370 | Bonferroni's post hoc test  Control-GFP vs. SUS-GFP, *p* < 0.001  SUS-GFP vs. SUS-sh*Cisd1*, *p* < 0.001 | Control-GFP  1.000 ± 0.100  SUS-GFP  3.077 ± 0.183  Control-sh*Cisd1*  1.234 ± 0.094  SUS-sh*Cisd1*  1.017 ± 0.077 |
| 2F: Relative *Tnf-a* mRNA expression  Control-GFP (n = 8)  SUS-GFP (n = 8)  Control-sh*Cisd1* (n = 8)  SUS-sh*Cisd1* (n = 8) | Two-way ANOVA  Stress: F (1, 28) = 162.400  Virus: F (1, 28) = 150.300 | Bonferroni's post hoc test  Control-GFP vs. SUS-GFP, *p* < 0.001  SUS-GFP vs. SUS-sh*Cisd1*, *p* < 0.001 | Control-GFP  1.000 ± 0.090  SUS-GFP  4.907 ± 0.237  Control-sh*Cisd1*  1.211 ± 0.099  SUS-sh*Cisd1*  1.071 ± 0.116 |
| 2H: IBA1^+^ cells/mm^2^  Control-GFP (n = 6)  SUS-GFP (n = 6)  Control-sh*Cisd1* (n = 6)  SUS-sh*Cisd1* (n = 6) | Two-way ANOVA  Stress: F (1, 20) = 33.680  Virus: F (1, 20) = 29.890 | Bonferroni's post hoc test  Control-GFP vs. SUS-GFP, *p* < 0.001  SUS-GFP vs. SUS-sh*Cisd1*, *p* < 0.001 | Control-GFP  93.797 ± 5.026  SUS-GFP  187.687 ± 13.862  Control-sh*Cisd1*  93.622 ± 6.335  SUS-sh*Cisd1*  96.598 ± 4.593 |
| 2I: IBA1 intensity (AU)  Control-GFP (n = 6)  SUS-GFP (n = 6)  Control-sh*Cisd1* (n = 6)  SUS-sh*Cisd1* (n = 6) | Two-way ANOVA  Stress: F (1, 20) = 5.695  Virus: F (1, 20) = 4.718 | Bonferroni's post hoc test  Control-GFP vs. SUS-GFP, *p* = 0.001  SUS-GFP vs. SUS-sh*Cisd1*, *p* = 0.002 | Control-GFP  1.000 ± 0.094  SUS-GFP  1.503 ± 0.093  Control-sh*Cisd1*  1.103 ± 0.086  SUS-sh*Cisd1*  1.019 ± 0.077 |
| 2K: Total process length (µm)  Control-GFP (n = 6)  SUS-GFP (n = 6)  Control-sh*Cisd1* (n = 6)  SUS-sh*Cisd1* (n = 6) | Two-way ANOVA  Stress: F (1, 20) = 16.830  Virus: F (1, 20) = 7.176 | Bonferroni's post hoc test  Control-GFP vs. SUS-GFP, *p* < 0.001  SUS-GFP vs. SUS-sh*Cisd1*, *p* = 0.003 | Control-GFP  690.788 ± 43.954  SUS-GFP  441.185 ± 24.770  Control-sh*Cisd1*  699.478 ± 42.368  SUS-sh*Cisd1*  636.550 ± 38.239 |
| 2L: Total branch number  Control-GFP (n = 6)  SUS-GFP (n = 6)  Control-sh*Cisd1* (n = 6)  SUS-sh*Cisd1* (n = 6) | Two-way ANOVA  Stress: F (1, 20) = 14.540  Virus: F (1, 20) = 19.030 | Bonferroni's post hoc test  Control-GFP vs. SUS-GFP, *p* < 0.001  SUS-GFP vs. SUS-sh*Cisd1*, *p* = 0.001 | Control-GFP  155.833 ± 10.242  SUS-GFP  79.500 ± 6.495  Control-sh*Cisd1*  151.500 ± 8.625  SUS-sh*Cisd1*  160.667 ± 9.422 |
| 2M: Normalized soma size  Control-GFP (n = 6)  SUS-GFP (n = 6)  Control-sh*Cisd1* (n = 6)  SUS-sh*Cisd1* (n = 6) | Two-way ANOVA  Stress: F (1, 20) = 5.956  Virus: F (1, 20) = 4.072 | Bonferroni's post hoc test  Control-GFP vs. SUS-GFP, *p* = 0.005  SUS-GFP vs. SUS-sh*Cisd1*, *p* = 0.010 | Control-GFP  1.000 ± 0.103  SUS-GFP  1.455 ± 0.118  Control-sh*Cisd1*  1.042 ± 0.077  SUS-sh*Cisd1*  1.039 ± 0.063 |
| 2O: Relative CD68^+^ area/IBA1 (%)  Control-GFP (n = 6)  SUS-GFP (n = 6)  Control-sh*Cisd1* (n = 6)  SUS-sh*Cisd1* (n = 6) | Two-way ANOVA  Stress: F (1, 20) = 26.100  Virus: F (1, 20) = 46.620 | Bonferroni's post hoc test  Control-GFP vs. SUS-GFP, *p* < 0.001  SUS-GFP vs. SUS-sh*Cisd1*, *p* < 0.001 | Control-GFP  3.892 ± 0.468  SUS-GFP  11.031 ± 0.749  Control-sh*Cisd1*  3.481 ± 0.602  SUS-sh*Cisd1*  4.979 ± 0.677 |
| 2Q: Relative IBA1 protein expression  Control-GFP (n = 3)  SUS-GFP (n = 3)  Control-sh*Cisd1* (n = 3)  SUS-sh*Cisd1* (n = 3) | Two-way ANOVA  Stress: F (1, 8) = 7.856  Virus: F (1, 8) = 42.820 | Bonferroni's post hoc test  Control-GFP vs. SUS-GFP, *p* < 0.001  SUS-GFP vs. SUS-sh*Cisd1*, *p* = 0.002 | Control-GFP  1.000 ± 0.103  SUS-GFP  2.006 ± 0.126  Control-sh*Cisd1*  1.132 ± 0.058  SUS-sh*Cisd1*  1.350 ± 0.071 |
| 3C: Social interaction ratio  Control-GFP (n = 6)  SUS-GFP (n = 6)  Control-Cisd1^cKO^ (n = 6)  SUS-Cisd1^cKO^ (n = 6) | Two-way ANOVA  Stress: F (1, 20) = 13.990  Virus: F (1, 20) = 11.750 | Bonferroni's post hoc test  Control-GFP vs. SUS-GFP, *p* < 0.001  SUS-GFP vs. SUS-Cisd1^cKO^, *p* < 0.001 | Control-GFP  1.761 ± 0.168  SUS-GFP  0.660 ± 0.056  Control-Cisd1^cKO^  1.767 ± 0.174  SUS-Cisd1^cKO^  1.712 ± 0.184 |
| 3D: Time in interaction zone (s)  Control-GFP (n = 6)  SUS-GFP (n = 6)  Control-Cisd1^cKO^ (n = 6)  SUS-Cisd1^cKO^ (n = 6) | Two-way ANOVA  Stress: F (3, 40) = 9.792  Target: F (1, 40) = 40.550 | Bonferroni's post hoc test  No target vs. Target  Control-GFP, *p* < 0.001  SUS-GFP, *p* = 0.031  Control-Cisd1^cKO^, *p* < 0.001  SUS-Cisd1^cKO^, *p* < 0.001 | No target  Control-GFP  21.083 ± 1.572  SUS-GFP  24.133 ± 1.500  Control-Cisd1^cKO^  21.217 ± 2.019  SUS-Cisd1^cKO^  22.433 ± 1.547  Target  Control-GFP  36.533 ± 3.128  SUS-GFP  16.000 ± 1.791  Control-Cisd1^cKO^  36.167 ± 2.426  SUS-Cisd1^cKO^  37.050 ± 1.865 |
| 3E: Time in corner zone (s)  Control-GFP (n = 6)  SUS-GFP (n = 6)  Control-Cisd1^cKO^ (n = 6)  SUS-Cisd1^cKO^ (n = 6) | Two-way ANOVA  Stress: F (3, 40) = 10.230  Target: F (1, 40) = 2.410 | Bonferroni's post hoc test  No target vs. Target  Control-GFP, *p*＞0.999  SUS-GFP, *p* < 0.001  Control-Cisd1^cKO^, *p*＞0.999  SUS-Cisd1^cKO^, *p*＞0.999 | No target  Control-GFP  22.917 ± 2.091  SUS-GFP  23.300 ± 2.117  Control-Cisd1^cKO^  22.900 ± 1.404  SUS-Cisd1^cKO^  23.467 ± 1.527  Target  Control-GFP  20.983 ± 1.265  SUS-GFP  38.617 ± 3.402  Control-Cisd1^cKO^  20.183 ± 1.991  SUS-Cisd1^cKO^  21.533 ± 1.165 |
| 3F: Sucrose preference  Control-GFP (n = 6)  SUS-GFP (n = 6)  Control-Cisd1^cKO^ (n = 6)  SUS-Cisd1^cKO^ (n = 6) | Two-way ANOVA  Stress: F (1, 20) = 19.090  Virus: F (1, 20) = 16.850 | Bonferroni's post hoc test  Control-GFP vs. SUS-GFP, *p* < 0.001  SUS-GFP vs. SUS-Cisd1^cKO^, *p* < 0.001 | Control-GFP  83.752 ± 1.449  SUS-GFP  67.292 ± 3.330  Control-Cisd1^cKO^  84.117 ± 1.255  SUS-Cisd1^cKO^  84.278 ± 1.002 |
| 3G: Total distance (m)  Control-GFP (n = 6)  SUS-GFP (n = 6)  Control-Cisd1^cKO^ (n = 6)  SUS-Cisd1^cKO^ (n = 6) | Two-way ANOVA  Stress: F (1, 20) = 0.052  Virus: F (1, 20) = 0.502 | Bonferroni's post hoc test  Control-GFP vs. SUS-GFP, *p* = 0.661  SUS-GFP vs. SUS-Cisd1^cKO^, *p* ＞0.999 | Control-GFP  43.486 ± 2.236  SUS-GFP  40.577 ± 1.270  Control-Cisd1^cKO^  42.506 ± 2.344  SUS-Cisd1^cKO^  42.494 ± 2.215 |
| 3I: IBA1^+^ cells/mm^2^  Control-GFP (n = 6)  SUS-GFP (n = 6)  Control-Cisd1^cKO^ (n = 6)  SUS-Cisd1^cKO^ (n = 6) | Two-way ANOVA  Stress: F (1, 20) = 27.850  Virus: F (1, 20) = 22.970 | Bonferroni's post hoc test  Control-GFP vs. SUS-GFP, *p* < 0.001  SUS-GFP vs. SUS-Cisd1^cKO^, *p* < 0.001 | Control-GFP  93.485 ± 4.293  SUS-GFP  174.752 ± 12.565  Control-Cisd1^cKO^  95.230 ± 6.349  SUS-Cisd1^cKO^  89.660 ± 5.726 |
| 3J: IBA1 intensity (AU)  Control-GFP (n = 6)  SUS-GFP (n = 6)  Control-Cisd1^cKO^ (n = 6)  SUS-Cisd1^cKO^ (n = 6) | Two-way ANOVA  Stress: F (1, 20) = 17.430  Virus: F (1, 20) = 13.550 | Bonferroni's post hoc test  Control-GFP vs. SUS-GFP, *p* < 0.001  SUS-GFP vs. SUS-Cisd1^cKO^, *p* < 0.001 | Control-GFP  1.000 ± 0.064  SUS-GFP  1.481 ± 0.068  Control-Cisd1^cKO^  0.964 ± 0.069  SUS-Cisd1^cKO^  0.968 ± 0.061 |
| 3L: Total process length (µm)  Control-GFP (n = 6)  SUS-GFP (n = 6)  Control-Cisd1^cKO^ (n = 6)  SUS-Cisd1^cKO^ (n = 6) | Two-way ANOVA  Stress: F (1, 20) = 8.691  Virus: F (1, 20) = 7.489 | Bonferroni's post hoc test  Control-GFP vs. SUS-GFP, *p* = 0.005  SUS-GFP vs. SUS-Cisd1^cKO^, *p* = 0.004 | Control-GFP  637.557 ± 35.740  SUS-GFP  466.745 ± 40.177  Control-Cisd1^cKO^  667.157 ± 31.269  SUS-Cisd1^cKO^  645.008 ± 33.200 |
| 3M: Total branch number  Control-GFP (n = 6)  SUS-GFP (n = 6)  Control-Cisd1^cKO^ (n = 6)  SUS-Cisd1^cKO^ (n = 6) | Two-way ANOVA  Stress: F (1, 20) = 23.830  Virus: F (1, 20) = 25.540 | Bonferroni's post hoc test  Control-GFP vs. SUS-GFP, *p* < 0.001  SUS-GFP vs. SUS-Cisd1^cKO^, *p* < 0.001 | Control-GFP  155.500 ± 7.894  SUS-GFP  96.167 ± 7.213  Control-Cisd1^cKO^  163.500 ± 6.756  SUS-Cisd1^cKO^  154.333 ± 4.869 |
| 3N: Normalized soma size  Control-GFP (n = 6)  SUS-GFP (n = 6)  Control-Cisd1^cKO^ (n = 6)  SUS-Cisd1^cKO^ (n = 6) | Two-way ANOVA  Stress: F (1, 20) = 21.010  Virus: F (1, 20) = 22.950 | Bonferroni's post hoc test  Control-GFP vs. SUS-GFP, *p* < 0.001  SUS-GFP vs. SUS-Cisd1^cKO^, *p* < 0.001 | Control-GFP  1.000 ± 0.077  SUS-GFP  1.607 ± 0.049  Control-Cisd1^cKO^  1.005 ± 0.071  SUS-Cisd1^cKO^  1.013 ± 0.055 |
| 3P: Relative CD68^+^ area/IBA1 (%)  Control-GFP (n = 6)  SUS-GFP (n = 6)  Control-Cisd1^cKO^ (n = 6)  SUS-Cisd1^cKO^ (n = 6) | Two-way ANOVA  Stress: F (1, 20) = 20.030  Virus: F (1, 20) = 27.410 | Bonferroni's post hoc test  Control-GFP vs. SUS-GFP, *p* < 0.001  SUS-GFP vs. SUS-Cisd1^cKO^, *p* < 0.001 | Control-GFP  5.034 ± 0.560  SUS-GFP  11.953 ± 0.764  Control-Cisd1^cKO^  4.586 ± 0.790  SUS-Cisd1^cKO^  5.610 ± 0.884 |
| 3R: Relative IBA1 protein expression  Control-GFP (n = 3)  SUS-GFP (n = 3)  Control-Cisd1^cKO^ (n = 3)  SUS-Cisd1^cKO^ (n = 3) | Two-way ANOVA  Stress: F (1, 8) = 18.470  Virus: F (1, 8) = 80.840 | Bonferroni's post hoc test  Control-GFP vs. SUS-GFP, *p* < 0.001  SUS-GFP vs. SUS-Cisd1^cKO^, *p* < 0.001 | Control-GFP  1.000 ± 0.065  SUS-GFP  2.328 ± 0.106  Control-Cisd1^cKO^  1.171 ± 0.100  SUS-Cisd1^cKO^  1.409 ± 0.070 |
| 4C: ATP production  Control-GFP (n = 5)  SUS-GFP (n = 5)  Control-Cisd1^cKO^ (n = 5)  SUS-Cisd1^cKO^ (n = 5) | Two-way ANOVA  Stress: F (1, 16) = 3.075  Virus: F (1, 16) = 3.006 | Bonferroni's post hoc test  Control-GFP vs. SUS-GFP, *p* = 0.024  SUS-GFP vs. SUS-Cisd1^cKO^, *p* = 0.023 | Control-GFP  34.157 ± 5.258  SUS-GFP  17.925 ± 4.943  Control-Cisd1^cKO^  32.064 ± 2.202  SUS-Cisd1^cKO^  34.238 ± 2.971 |
| 4D: Basal respiration  Control-GFP (n = 5)  SUS-GFP (n = 5)  Control-Cisd1^cKO^ (n = 5)  SUS-Cisd1^cKO^ (n = 5) | Two-way ANOVA  Stress: F (1, 16) = 8.469  Virus: F (1, 16) = 1.133 | Bonferroni's post hoc test  Control-GFP vs. SUS-GFP, *p* = 0.034  SUS-GFP vs. SUS-Cisd1^cKO^, *p* = 0.002 | Control-GFP  36.405 ± 2.866  SUS-GFP  24.330 ± 3.679  Control-Cisd1^cKO^  37.062 ± 2.692  SUS-Cisd1^cKO^  42.317 ± 3.471 |
| 4E: Maximal respiration  Control-GFP (n = 5)  SUS-GFP (n = 5)  Control-Cisd1^cKO^ (n = 5)  SUS-Cisd1^cKO^ (n = 5) | Two-way ANOVA  Stress: F (1, 16) = 9.173  Virus: F (1, 16) = 18.380 | Bonferroni's post hoc test  Control-GFP vs. SUS-GFP, *p* < 0.001  SUS-GFP vs. SUS-Cisd1^cKO^, *p* < 0.001 | Control-GFP  87.551 ± 5.183  SUS-GFP  46.209 ± 7.296  Control-Cisd1^cKO^  83.337 ± 2.076  SUS-Cisd1^cKO^  81.164 ± 4.315 |
| 4F: Reserve capacity  Control-GFP (n = 5)  SUS-GFP (n = 5)  Control-Cisd1^cKO^ (n = 5)  SUS-Cisd1^cKO^ (n = 5) | Two-way ANOVA  Stress: F (1, 16) = 1.340  Virus: F (1, 16) = 10.280 | Bonferroni's post hoc test  Control-GFP vs. SUS-GFP, *p* = 0.004  SUS-GFP vs. SUS-Cisd1^cKO^, *p* = 0.083 | Control-GFP  51.145 ± 6.570  SUS-GFP  20.079 ± 6.887  Control-Cisd1^cKO^  46.274 ± 4.462  SUS-Cisd1^cKO^  38.847 ± 5.797 |
| 4H: Basal glycolysis  Control-GFP (n = 5)  SUS-GFP (n = 5)  Control-Cisd1^cKO^ (n = 5)  SUS-Cisd1^cKO^ (n = 5) | Two-way ANOVA  Stress: F (1, 16) = 2.548  Virus: F (1, 16) = 3.738 | Bonferroni's post hoc test  Control-GFP vs. SUS-GFP, *p* = 0.025  SUS-GFP vs. SUS-Cisd1^cKO^, *p* = 0.041 | Control-GFP  16.090 ± 2.070  SUS-GFP  24.125 ± 2.038  Control-Cisd1^cKO^  16.997 ± 1.645  SUS-Cisd1^cKO^  16.771 ± 2.273 |
| 4I: Maximal glycolysis  Control-GFP (n = 5)  SUS-GFP (n = 5)  Control-Cisd1^cKO^ (n = 5)  SUS-Cisd1^cKO^ (n = 5) | Two-way ANOVA  Stress: F (1, 16) = 8.125  Virus: F (1, 16) = 11.180 | Bonferroni's post hoc test  Control-GFP vs. SUS-GFP, *p* = 0.002  SUS-GFP vs. SUS-Cisd1^cKO^, *p* = 0.005 | Control-GFP  28.351 ± 1.678  SUS-GFP  36.076 ± 1.475  Control-Cisd1^cKO^  27.537 ± 0.822  SUS-Cisd1^cKO^  29.031 ± 1.391 |
| 5B: Relative *Cisd1* mRNA expression  Vehicle (n = 6)  LPS+ATP (n = 6) | Unpaired *t* test  t = 6.639, df = 10, *p* < 0.001 |  | Vehicle  1.000 ± 0.055  LPS+ATP  2.573 ± 0.230 |
| 5C: Relative CISD1 protein expression  Vehicle (n = 5)  LPS+ATP (n = 5) | Unpaired *t* test  t = 6.078, df = 8, *p* < 0.001 |  | Vehicle  1.000 ± 0.075  LPS+ATP  1.621 ± 0.069 |
| 5E: ATP production  Vehicle (n = 5)  LPS+ATP (n = 5)  Vehicle-NL-1 (n = 5)  LPS+ATP-NL-1 (n = 5) | One-way ANOVA  LPS+ATP x NL-1:  F (3, 16) = 5.047 | Bonferroni's post hoc test  Vehicle vs. LPS+ATP, *p* = 0.014  LPS+ATP vs. LPS+ATP-NL-1, *p* = 0.011 | Vehicle  170.757 ± 3.795  LPS+ATP  159.592 ± 0.794  Vehicle-NL-1  168.197 ± 1.640  LPS+ATP-NL-1  171.104 ± 2.271 |
| 5F: Basal respiration  Vehicle (n = 5)  LPS+ATP (n = 5)  Vehicle-NL-1 (n = 5)  LPS+ATP-NL-1 (n = 5) | One-way ANOVA  LPS+ATP x NL-1:  F (3, 16) = 6.017 | Bonferroni's post hoc test  Vehicle vs. LPS+ATP, *p* = 0.013  LPS+ATP vs. LPS+ATP-NL-1, *p* = 0.004 | Vehicle  176.680 ± 1.713  LPS+ATP  168.063 ± 0.871  Vehicle-NL-1  174.796 ± 2.486  LPS+ATP-NL-1  178.250 ± 1.869 |
| 5G: Maximal respiration  Vehicle (n = 5)  LPS+ATP (n = 5)  Vehicle-NL-1 (n = 5)  LPS+ATP-NL-1 (n = 5) | One-way ANOVA  LPS+ATP x NL-1:  F (3, 16) = 17.320 | Bonferroni's post hoc test  Vehicle vs. LPS+ATP, *p* < 0.001  LPS+ATP vs. LPS+ATP-NL-1, *p* < 0.001 | Vehicle  214.484 ± 1.944  LPS+ATP  191.876 ± 3.393  Vehicle-NL-1  208.270 ± 0.953  LPS+ATP-NL-1  208.898 ± 2.395 |
| 5H: Reserve capacity  Vehicle (n = 5)  LPS+ATP (n = 5)  Vehicle-NL-1 (n = 5)  LPS+ATP-NL-1 (n = 5) | One-way ANOVA  LPS+ATP x NL-1:  F (3, 16) = 3.127 | Bonferroni's post hoc test  Vehicle vs. LPS+ATP, *p* = 0.027  LPS+ATP vs. LPS+ATP-NL-1, *p* = 0.495 | Vehicle  37.804 ± 2.501  LPS+ATP  23.813 ± 3.992  Vehicle-NL-1  33.474 ± 2.996  LPS+ATP-NL-1  30.647 ± 3.597 |
| 5J: Basal glycolysis  Vehicle (n = 5)  LPS+ATP (n = 5)  Vehicle-NL-1 (n = 5)  LPS+ATP-NL-1 (n = 5) | One-way ANOVA  LPS+ATP x NL-1:  F (3, 16) = 9.420 | Bonferroni's post hoc test  Vehicle vs. LPS+ATP, *p* = 0.001  LPS+ATP vs. LPS+ATP-NL-1, *p* = 0.002 | Vehicle  15.757 ± 2.003  LPS+ATP  26.925 ± 1.308  Vehicle-NL-1  16.597 ± 1.618  LPS+ATP-NL-1  16.438 ± 1.945 |
| 5K: Maximal glycolysis  Vehicle (n = 5)  LPS+ATP (n = 5)  Vehicle-NL-1 (n = 5)  LPS+ATP-NL-1 (n = 5) | One-way ANOVA  LPS+ATP x NL-1:  F (3, 16) = 6.523 | Bonferroni's post hoc test  Vehicle vs. LPS+ATP, *p* = 0.004  LPS+ATP vs. LPS+ATP-NL-1, *p* = 0.005 | Vehicle  39.817 ± 2.684  LPS+ATP  53.609 ± 2.971  Vehicle-NL-1  43.470 ± 2.627  LPS+ATP-NL-1  39.964 ± 1.692 |
| 5M: Relative CISD1 protein expression  Vehicle (n = 4)  LPS+ATP (n = 4) | Unpaired *t* test  t = 9.684, df = 6, *p* < 0.001 |  | Vehicle  1.000 ± 0.057  LPS+ATP  1.803 ± 0.061 |
| 5O: Basal glycolysis  Vehicle (n = 5)  LPS+ATP (n = 5)  Vehicle-NL-1 (n = 5)  LPS+ATP-NL-1 (n = 5) | One-way ANOVA  LPS+ATP x NL-1:  F (3, 16) = 7.641 | Bonferroni's post hoc test  Vehicle vs. LPS+ATP, *p* = 0.014  LPS+ATP vs. LPS+ATP-NL-1, *p* = 0.018 | Vehicle  47.157 ± 2.308  LPS+ATP  57.858 ± 1.206  Vehicle-NL-1  42.931 ± 3.346  LPS+ATP-NL-1  47.571 ± 1.756 |
| 5P: Maximal glycolysis  Vehicle (n = 5)  LPS+ATP (n = 5)  Vehicle-NL-1 (n = 5)  LPS+ATP-NL-1 (n = 5) | One-way ANOVA  LPS+ATP x NL-1:  F (3, 16) = 5.964 | Bonferroni's post hoc test  Vehicle vs. LPS+ATP, *p* = 0.018  LPS+ATP vs. LPS+ATP-NL-1, *p* = 0.008 | Vehicle  70.284 ± 2.297  LPS+ATP  84.543 ± 2.889  Vehicle-NL-1  68.470 ± 3.970  LPS+ATP-NL-1  68.631 ± 3.302 |
| 6C: Social interaction ratio  Control-vehicle (n = 7)  SUS-vehicle (n = 7)  Control-pioglitazone  (n = 7)  SUS-pioglitazone  (n = 7) | Two-way ANOVA  Stress: F (1, 24) = 12.220  Pioglitazone: F (1, 24) = 17.350 | Bonferroni's post hoc test  Control-vehicle vs. SUS-vehicle, *p* < 0.001  SUS-vehicle vs. SUS-pioglitazone, *p* < 0.001 | Control-vehicle  1.646 ± 0.096  SUS-vehicle  0.560 ± 0.062  Control-pioglitazone  1.644 ± 0.196  SUS-pioglitazone 1.551 ± 0.169 |
| 6D: Time in interaction zone (s)  Control-vehicle (n = 7)  SUS-vehicle (n = 7)  Control-pioglitazone  (n = 7)  SUS-pioglitazone  (n = 7) | Two-way ANOVA  Stress: F (3, 48) = 3.940  Target: F (1, 48) = 9.932 | Bonferroni's post hoc test  No target vs. Target  Control-vehicle, *p* = 0.008  SUS-vehicle, *p* = 0.049  Control-pioglitazone,  *p* = 0.019  SUS-pioglitazone,  *p* = 0.042 | No target  Control-vehicle  23.371 ± 1.931  SUS-vehicle  26.286 ± 1.780  Control-pioglitazone  21.600 ± 2.848  SUS-pioglitazone 21.929 ± 1.774  Target  Control-vehicle  38.157 ± 3.570  SUS-vehicle  14.514 ± 1.586  Control-pioglitazone  35.000 ± 5.260  SUS-pioglitazone 33.971 ± 4.522 |
| 6E: Time in corner zone (s)  Control-vehicle (n = 7)  SUS-vehicle (n = 7)  Control-pioglitazone  (n = 7)  SUS-pioglitazone  (n = 7) | Two-way ANOVA  Stress: F (3, 48) = 5.289  Target: F (1, 48) = 4.094 | Bonferroni's post hoc test  No target vs. Target  Control-vehicle, *p*＞0.999  SUS-vehicle, *p* < 0.001  Control-pioglitazone,  *P*＞0.999  SUS-pioglitazone,  *P*＞0.999 | No target  Control-vehicle  26.386 ± 1.782  SUS-vehicle  22.257 ± 2.025  Control-pioglitazone  23.914 ± 1.723  SUS-pioglitazone 23.643 ± 1.459  Target  Control-vehicle  24.086 ± 2.074  SUS-vehicle  37.343 ± 2.132  Control-pioglitazone  22.600 ± 2.057  SUS-pioglitazone 22.986 ± 1.766 |
| 6F: Sucrose preference  Control-vehicle (n = 7)  SUS-vehicle (n = 7)  Control-pioglitazone  (n = 7)  SUS-pioglitazone  (n = 7) | Two-way ANOVA  Stress: F (1, 24) = 23.040  Pioglitazone: F (1, 24) = 19.340 | Bonferroni's post hoc test  Control-vehicle vs. SUS-vehicle, *p* < 0.001  SUS-vehicle vs. SUS-pioglitazone, *p* < 0.001 | Control-vehicle  82.044 ± 1.220  SUS-vehicle  66.486 ± 2.886  Control-pioglitazone  83.279 ± 1.357  SUS-pioglitazone 82.780 ± 1.294 |
| 6G: Relative *Il1b* mRNA expression  Control-vehicle (n = 7)  SUS-vehicle (n = 7)  Control-pioglitazone  (n = 7)  SUS-pioglitazone  (n = 7) | Two-way ANOVA  Stress: F (1, 24) = 72.560  Pioglitazone: F (1, 24) = 91.680 | Bonferroni's post hoc test  Control-vehicle vs. SUS-vehicle, *p* < 0.001  SUS-vehicle vs. SUS-pioglitazone, *p* < 0.001 | Control-vehicle  1.000 ± 0.055  SUS-vehicle  4.743 ± 0.389  Control-pioglitazone  0.987 ± 0.064  SUS-pioglitazone 1.219 ± 0.118 |
| 6H: Relative *Il6* mRNA expression  Control-vehicle (n = 7)  SUS-vehicle (n = 7)  Control-pioglitazone  (n = 7)  SUS-pioglitazone  (n = 7) | Two-way ANOVA  Stress: F (1, 24) = 66.580  Pioglitazone: F (1, 24) = 72.340 | Bonferroni's post hoc test  Control-vehicle vs. SUS-vehicle, *p* < 0.001  SUS-vehicle vs. SUS-pioglitazone, *p* < 0.001 | Control-vehicle  1.000 ± 0.046  SUS-vehicle  2.680 ± 0.175  Control-pioglitazone  1.025 ± 0.057  SUS-pioglitazone 1.034 ± 0.060 |
| 6I: Relative *Tnf-a* mRNA expression  Control-vehicle (n = 7)  SUS-vehicle (n = 7)  Control-pioglitazone  (n = 7)  SUS-pioglitazone  (n = 7) | Two-way ANOVA  Stress: F (1, 24) = 140.700  Pioglitazone: F (1, 24) = 166.000 | Bonferroni's post hoc test  Control-vehicle vs. SUS-vehicle, *p* < 0.001  SUS-vehicle vs. SUS-pioglitazone, *p* < 0.001 | Control-vehicle  1.000 ± 0.054  SUS-vehicle  4.975 ± 0.302  Control-pioglitazone  1.028 ± 0.053  SUS-pioglitazone 1.163 ± 0.069 |
| 6J: IL-1β (pg/ml)  Control-vehicle (n = 6)  SUS-vehicle (n = 6)  Control-pioglitazone  (n = 6)  SUS-pioglitazone  (n = 6) | Two-way ANOVA  Stress: F (1, 20) = 61.370  Pioglitazone: F (1, 20) = 56.140 | Bonferroni's post hoc test  Control-vehicle vs. SUS-vehicle, *p* < 0.001  SUS-vehicle vs. SUS-pioglitazone, *p* < 0.001 | Control-vehicle  95.870 ± 4.611  SUS-vehicle  205.696 ± 12.056  Control-pioglitazone  92.843 ± 5.216  SUS-pioglitazone 93.360 ± 4.801 |
| 6K: IL-6 (pg/ml)  Control-vehicle (n = 6)  SUS-vehicle (n = 6)  Control-pioglitazone  (n = 6)  SUS-pioglitazone  (n = 6) | Two-way ANOVA  Stress: F (1, 20) = 29.860  Pioglitazone: F (1, 20) = 33.810 | Bonferroni's post hoc test  Control-vehicle vs. SUS-vehicle, *p* < 0.001  SUS-vehicle vs. SUS-pioglitazone, *p* < 0.001 | Control-vehicle  31.567 ± 3.194  SUS-vehicle  67.526 ± 3.954  Control-pioglitazone  33.071 ± 2.305  SUS-pioglitazone 32.636 ± 2.486 |
| 6L: TNF-α (pg/ml)  Control-vehicle (n = 6)  SUS-vehicle (n = 6)  Control-pioglitazone  (n = 6)  SUS-pioglitazone  (n = 6) | Two-way ANOVA  Stress: F (1, 20) = 96.560  Pioglitazone: F (1, 20) = 117.900 | Bonferroni's post hoc test  Control-vehicle vs. SUS-vehicle, *p* < 0.001  SUS-vehicle vs. SUS-pioglitazone, *p* < 0.001 | Control-vehicle  47.357 ± 4.563  SUS-vehicle  192.625 ± 10.188  Control-pioglitazone  51.762 ± 6.354  SUS-pioglitazone 54.390 ± 4.527 |
| 6M: Relative *Hmox1* mRNA expression  Control-vehicle (n = 7)  SUS-vehicle (n = 7)  Control-pioglitazone  (n = 7)  SUS-pioglitazone  (n = 7) | Two-way ANOVA  Stress: F (1, 24) = 16.050  Pioglitazone: F (1, 24) = 16.540 | Bonferroni's post hoc test  Control-vehicle vs. SUS-vehicle, *p* < 0.001  SUS-vehicle vs. SUS-pioglitazone, *p* < 0.001 | Control-vehicle  1.000 ± 0.073  SUS-vehicle  0.531 ± 0.066  Control-pioglitazone  1.045 ± 0.067  SUS-pioglitazone 0.996 ± 0.045 |
| 6N: Relative *Sod1* mRNA expression  Control-vehicle (n = 7)  SUS-vehicle (n = 7)  Control-pioglitazone  (n = 7)  SUS-pioglitazone  (n = 7) | Two-way ANOVA  Stress: F (1, 24) = 6.835  Pioglitazone: F (1, 24) = 15.870 | Bonferroni's post hoc test  Control-vehicle vs. SUS-vehicle, *p* < 0.001  SUS-vehicle vs. SUS-pioglitazone, *p* = 0.001 | Control-vehicle  1.000 ± 0.061  SUS-vehicle  0.563 ± 0.071  Control-pioglitazone  0.977 ± 0.065  SUS-pioglitazone 0.914 ± 0.052 |
| 6O: Relative *Sod2* mRNA expression  Control-vehicle (n = 7)  SUS-vehicle (n = 7)  Control-pioglitazone  (n = 7)  SUS-pioglitazone  (n = 7) | Two-way ANOVA  Stress: F (1, 24) = 20.410  Pioglitazone: F (1, 24) = 21.710 | Bonferroni's post hoc test  Control-vehicle vs. SUS-vehicle, *p* < 0.001  SUS-vehicle vs. SUS-pioglitazone, *p* < 0.001 | Control-vehicle  1.000 ± 0.051  SUS-vehicle  0.479 ± 0.053  Control-pioglitazone  1.038 ± 0.067  SUS-pioglitazone 0.991 ± 0.071 |
| 6P: NAD^+^/NADH ratio  Control-vehicle (n = 7)  SUS-vehicle (n = 7)  Control-pioglitazone  (n = 7)  SUS-pioglitazone  (n = 7) | Two-way ANOVA  Stress: F (1, 24) = 4.028  Pioglitazone: F (1, 24) = 14.770 | Bonferroni's post hoc test  Control-vehicle vs. SUS-vehicle, *p* < 0.001  SUS-vehicle vs. SUS-pioglitazone, *p* = 0.039 | Control-vehicle  3.000 ± 0.194  SUS-vehicle  1.914 ± 0.123  Control-pioglitazone  3.095 ± 0.231  SUS-pioglitazone 2.629 ± 0.239 |
| 6Q: GAPDH activity  Control-vehicle (n = 6)  SUS-vehicle (n = 6)  Control-pioglitazone  (n = 6)  SUS-pioglitazone  (n = 6) | Two-way ANOVA  Stress: F (1, 20) = 9.642  Pioglitazone: F (1, 20) = 16.240 | Bonferroni's post hoc test  Control-vehicle vs. SUS-vehicle, *p* < 0.001  SUS-vehicle vs. SUS-pioglitazone, *p* = 0.004 | Control-vehicle  1.000 ± 0.085  SUS-vehicle  1.432 ± 0.062  Control-pioglitazone  0.916 ± 0.079  SUS-pioglitazone 1.067 ± 0.060 |
| 6R: Complex I (nmol/min/mg protein)  Control-vehicle (n = 5)  SUS-vehicle (n = 5)  Control-pioglitazone  (n = 5)  SUS-pioglitazone  (n = 5) | Two-way ANOVA  Stress: F (1, 16) = 7.324  Pioglitazone: F (1, 16) = 14.430 | Bonferroni's post hoc test  Control-vehicle vs. SUS-vehicle, *p* < 0.001  SUS-vehicle vs. SUS-pioglitazone, *p* = 0.005 | Control-vehicle  20.064 ± 1.046  SUS-vehicle  11.249 ± 1.614  Control-pioglitazone  20.571 ± 1.846  SUS-pioglitazone 18.498 ± 1.052 |
| 6S: Complex II (nmol/min/mg protein)  Control-vehicle (n = 5)  SUS-vehicle (n = 5)  Control-pioglitazone  (n = 5)  SUS-pioglitazone  (n = 5) | Two-way ANOVA  Stress: F (1, 16) = 15.170  Pioglitazone: F (1, 16) = 8.336 | Bonferroni's post hoc test  Control-vehicle vs. SUS-vehicle, *p* = 0.002  SUS-vehicle vs. SUS-pioglitazone, *p* < 0.001 | Control-vehicle  16.496 ± 0.733  SUS-vehicle  12.893 ± 0.571  Control-pioglitazone  17.143 ± 0.519  SUS-pioglitazone 17.127 ± 0.662 |
| 7B: NAD^+^/NADH ratio  Vehicle (n = 5)  LPS+ATP (n = 5)  Vehicle-pioglitazone  (n = 5)  LPS+ATP-pioglitazone  (n = 5) | One-way ANOVA  LPS+ATP x pioglitazone: F (3, 16) = 7.202 | Bonferroni's post hoc test  Vehicle vs. LPS+ATP,  *p* = 0.002  LPS+ATP vs. LPS+ATP-pioglitazone, *p* = 0.015 | Vehicle  2.436 ± 0.183  LPS+ATP  1.597 ± 0.134  Vehicle-pioglitazone  2.309 ± 0.130  LPS+ATP-pioglitazone  2.240 ± 0.097 |
| 7C: Complex I (nmol/min/mg protein)  Vehicle (n = 5)  LPS+ATP (n = 5)  Vehicle-pioglitazone  (n = 5)  LPS+ATP-pioglitazone  (n = 5) | One-way ANOVA  LPS+ATP x pioglitazone: F (3, 16) = 14.490 | Bonferroni's post hoc test  Vehicle vs. LPS+ATP,  *p* < 0.001  LPS+ATP vs. LPS+ATP-pioglitazone, *p* < 0.001 | Vehicle  19.321 ± 1.337  LPS+ATP  9.253 ± 0.904  Vehicle-pioglitazone  19.620 ± 1.688  LPS+ATP-pioglitazone  18.159 ± 1.122 |
| 7D: Complex II (nmol/min/mg protein)  Vehicle (n = 5)  LPS+ATP (n = 5)  Vehicle-pioglitazone  (n = 5)  LPS+ATP-pioglitazone  (n = 5) | One-way ANOVA  LPS+ATP x pioglitazone: F (3, 16) = 7.125 | Bonferroni's post hoc test  Vehicle vs. LPS+ATP,  *p* = 0.031  LPS+ATP vs. LPS+ATP-pioglitazone, *p* = 0.002 | Vehicle  11.517 ± 0.785  LPS+ATP  8.485 ± 0.654  Vehicle-pioglitazone  12.377 ± 0.611  LPS+ATP-pioglitazone  12.894 ± 0.876 |
| 7G: ATP production  Vehicle (n = 5)  LPS+ATP (n = 5)  Vehicle-pioglitazone  (n = 5)  LPS+ATP-pioglitazone  (n = 5) | One-way ANOVA  LPS+ATP x pioglitazone: F (3, 16) = 5.303 | Bonferroni's post hoc test  Vehicle vs. LPS+ATP, *p* = 0.038  LPS+ATP vs. LPS+ATP-pioglitazone, *p* = 0.012 | Vehicle  154.024 ± 5.003  LPS+ATP  139.192 ± 2.925  Vehicle-pioglitazone  157.397 ± 2.783  LPS+ATP-pioglitazone  156.971 ± 3.804 |
| 7H: Basal respiration  Vehicle (n = 5)  LPS+ATP (n = 5)  Vehicle-pioglitazone  (n = 5)  LPS+ATP-pioglitazone  (n = 5) | One-way ANOVA  LPS+ATP x pioglitazone: F (3, 16) = 7.534 | Bonferroni's post hoc test  Vehicle vs. LPS+ATP, *p* = 0.005  LPS+ATP vs. LPS+ATP-pioglitazone, *p* = 0.004 | Vehicle  167.139 ± 4.644  LPS+ATP  146.063 ± 3.412  Vehicle-pioglitazone  168.262 ± 1.742  LPS+ATP-pioglitazone  167.984 ± 5.154 |
| 7I: Maximal respiration  Vehicle (n = 5)  LPS+ATP (n = 5)  Vehicle-pioglitazone  (n = 5)  LPS+ATP-pioglitazone  (n = 5) | One-way ANOVA  LPS+ATP x pioglitazone: F (3, 16) = 34.010 | Bonferroni's post hoc test  Vehicle vs. LPS+ATP, *p* < 0.001  LPS+ATP vs. LPS+ATP-pioglitazone, *p* < 0.001 | Vehicle  212.817 ± 5.674  LPS+ATP  163.676 ± 3.160  Vehicle-pioglitazone  207.070 ± 3.356  LPS+ATP-pioglitazone  212.231 ± 3.518 |
| 7J: Reserve capacity  Vehicle (n = 5)  LPS+ATP (n = 5)  Vehicle-pioglitazone  (n = 5)  LPS+ATP-pioglitazone  (n = 5) | One-way ANOVA  LPS+ATP x pioglitazone: F (3, 16) = 5.169 | Bonferroni's post hoc test  Vehicle vs. LPS+ATP, *p* = 0.009  LPS+ATP vs. LPS+ATP-pioglitazone, *p* = 0.014 | Vehicle  45.678 ± 8.999  LPS+ATP  17.613 ± 3.149  Vehicle-pioglitazone  38.808 ± 3.795  LPS+ATP-pioglitazone  44.247 ± 5.028 |
| 7L: Basal glycolysis  Vehicle (n = 5)  LPS+ATP (n = 5)  Vehicle-pioglitazone  (n = 5)  LPS+ATP-pioglitazone  (n = 5) | One-way ANOVA  LPS+ATP x pioglitazone: F (3, 16) = 12.810 | Bonferroni's post hoc test  Vehicle vs. LPS+ATP, *p* < 0.001  LPS+ATP vs. LPS+ATP-pioglitazone, *p* = 0.001 | Vehicle  15.890 ± 1.815  LPS+ATP  27.392 ± 1.299  Vehicle-pioglitazone  17.264 ± 1.752  LPS+ATP-pioglitazone  18.171 ± 0.692 |
| 7M: Maximal glycolysis  Vehicle (n = 5)  LPS+ATP (n = 5)  Vehicle-pioglitazone  (n = 5)  LPS+ATP-pioglitazone  (n = 5) | One-way ANOVA  LPS+ATP x pioglitazone: F (3, 16) = 11.400 | Bonferroni's post hoc test  Vehicle vs. LPS+ATP, *p* < 0.001  LPS+ATP vs. LPS+ATP-pioglitazone, *p* = 0.001 | Vehicle  39.751 ± 1.499  LPS+ATP  50.076 ± 1.322  Vehicle-pioglitazone  38.337 ± 1.278  LPS+ATP-pioglitazone  39.897 ± 2.162 |
| Figure S1B: Social interaction ratio  Control (n = 8)  Susceptible (n = 7)  Resilient (n = 5) | One-way ANOVA  Stress: F (2, 17) = 29.600 | Bonferroni's post hoc test  Control vs. susceptible, *p* < 0.001  Control vs. resilient, *p* = 0.669 | Control  1.735 ± 0.106  Susceptible  0.764 ± 0.037  Resilient  1.590 ± 0.145 |
| Figure S1C: Time in interaction zone (s)  Control (n = 8)  Susceptible (n = 7)  Resilient (n = 5) | Two-way ANOVA  Stress: F (1, 34) = 14.880  Target: F (2, 34) = 6.643 | Bonferroni's post hoc test  Target  Control vs. susceptible, *p* < 0.001  Control vs. resilient, *p* = 0.690 | No target  Control  21.463 ± 1.493  Susceptible  24.714 ± 1.416  Resilient  21.900 ± 2.182  Target  Control  37.113 ± 3.337  Susceptible  18.857 ± 1.386  Resilient  33.920 ± 2.770 |
| Figure S1D: Time in corner zone (s)  Control (n = 8)  Susceptible (n = 7)  Resilient (n = 5) | Two-way ANOVA  Stress: F (1, 34) = 13.840  Target: F (2, 34) = 17.420 | Bonferroni's post hoc test  Target  Control vs. susceptible, *p* < 0.001  Control vs. resilient, *p*＞0.999 | No target  Control  20.175 ± 1.525  Susceptible  21.029 ± 1.431  Resilient  21.280 ± 2.018  Target  Control  19.338 ± 0.969  Susceptible  44.143 ± 4.713  Resilient  21.560 ± 1.628 |
| Figure S1E: Sucrose preference  Control (n = 8)  Susceptible (n = 7)  Resilient (n = 5) | One-way ANOVA  Stress: F (2, 17) = 19.740 | Bonferroni's post hoc test  Control vs. susceptible, *p* < 0.001  Control vs. resilient, *p*＞0.999 | Control  83.893 ± 1.751  Susceptible  66.304 ± 3.010  Resilient  82.408 ± 0.946 |
| Figure S1F: Immobility time in FST (s)  Control (n = 8)  Susceptible (n = 7)  Resilient (n = 5) | One-way ANOVA  Stress: F (2, 17) = 14.560 | Bonferroni's post hoc test  Control vs. susceptible, *p* < 0.001  Control vs. resilient, *p*＞0.999 | Control  80.513 ± 6.467  Susceptible  120.514 ± 5.846  Resilient  80.480 ± 4.870 |
| Figure S1G: Immobility time in TST (s)  Control (n = 8)  Susceptible (n = 7)  Resilient (n = 5) | One-way ANOVA  Stress: F (2, 17) = 11.820 | Bonferroni's post hoc test  Control vs. susceptible, *p* = 0.001  Control vs. resilient, *p*＞0.999 | Control  90.800 ± 4.868  Susceptible  122.571 ± 6.455  Resilient  86.680 ± 5.481 |
| Figure S2B: Relative *Cisd1* mRNA expression  Control (n = 8)  Susceptible (n = 7)  Resilient (n = 5) | Two-way ANOVA  Stress: F (2, 51) = 13.810 | Bonferroni's post hoc test  mPFC  Control vs. susceptible, *p* < 0.001  Control vs. resilient, *p*＞0.999  Hippocampus  Control vs. susceptible, *p* ＞0.999  Control vs. resilient, *p*＞0.999  NAc  Control vs. susceptible, *p* ＞0.999  Control vs. resilient, *p*＞0.999 | mPFC  Control  1.000 ± 0.044  Susceptible  1.681 ± 0.048  Resilient  1.019 ± 0.077  Hippocampus  Control  1.000 ± 0.047  Susceptible  0.981 ± 0.059  Resilient  1.026 ± 0.101  NAc  Control  1.000 ± 0.054  Susceptible  0.979 ± 0.068  Resilient  1.012 ± 0.082 |
| Figure S2D: NAD^+^/NADH ratio  Control (n = 7)  Susceptible (n = 7)  Resilient (n = 5) | Two-way ANOVA  Stress: F (2, 48) = 0.696 | Bonferroni's post hoc test  mPFC  Control vs. susceptible, *p* = 0.003  Control vs. resilient, *p* = 0.948  Hippocampus  Control vs. susceptible, *p* = 0.023  Control vs. resilient, *p*＞0.999  NAc  Control vs. susceptible, *p* = 0.720  Control vs. resilient, *p*＞0.999 | mPFC  Control  2.595 ± 0.171  Susceptible  1.792 ± 0.121  Resilient  2.782 ± 0.189  Hippocampus  Control  2.489 ± 0.175  Susceptible  1.864 ± 0.089  Resilient  2.324 ± 0.236  NAc  Control  2.431 ± 0.163  Susceptible  2.212 ± 0.184  Resilient  2.414 ± 0.285 |
| Figure S2E: Relative mRNA expression  Control (n = 8)  Susceptible (n = 8)  Resilient (n = 5) | Two-way ANOVA  Stress: F (2, 54) = 2.928 | Bonferroni's post hoc test  *Hmox1*  Control vs. susceptible, *p* < 0.001  Control vs. resilient, *p* = 0.353  *Sod1*  Control vs. susceptible, *p* < 0.001  Control vs. resilient, *p*＞0.999  *Sod2*  Control vs. susceptible, *p* < 0.001  Control vs. resilient, *p*＞0.999 | *Hmox1*  Control  1.000 ± 0.045  Susceptible  0.379 ± 0.056  Resilient  0.885 ± 0.051  *Sod1*  Control  1.000 ± 0.065  Susceptible  0.536 ± 0.058  Resilient  1.054 ± 0.051  *Sod2*  Control  1.000 ± 0.048  Susceptible  0.398 ± 0.053  Resilient  0.946 ± 0.060 |
| Figure S2F: Relative mRNA expression  Control (n = 8)  Susceptible (n = 8)  Resilient (n = 5) | Two-way ANOVA  Stress: F (2, 54) = 0.239 | Bonferroni's post hoc test  *Hmox1*  Control vs. susceptible, *p* = 0.042  Control vs. resilient, *p*＞0.999  *Sod1*  Control vs. susceptible, *p* = 0.344  Control vs. resilient, *p* = 0.977  *Sod2*  Control vs. susceptible, *p* = 0.111  Control vs. resilient, *p*＞0.999 | *Hmox1*  Control  1.000 ± 0.047  Susceptible  0.842 ± 0.056  Resilient  0.965 ± 0.049  *Sod1*  Control  1.000 ± 0.049  Susceptible  0.908 ± 0.044  Resilient  0.947 ± 0.039  *Sod2*  Control  1.000 ± 0.038  Susceptible  0.869 ± 0.049  Resilient  1.025 ± 0.079 |
| Figure S2G: Relative mRNA expression  Control (n = 8)  Susceptible (n = 8)  Resilient (n = 5) | Two-way ANOVA  Stress: F (2, 54) = 0.863 | Bonferroni's post hoc test  *Hmox1*  Control vs. susceptible, *p*＞0.999  Control vs. resilient,  *p*＞0.999  *Sod1*  Control vs. susceptible, *p* = 0.825  Control vs. resilient, *p* = 0.964  *Sod2*  Control vs. susceptible, *p* = 0.955  Control vs. resilient, *p*＞0.999 | *Hmox1*  Control  1.000 ± 0.049  Susceptible  0.984 ± 0.050  Resilient  0.969 ± 0.057  *Sod1*  Control  1.000 ± 0.054  Susceptible  0.940 ± 0.055  Resilient  0.941 ± 0.060  *Sod2*  Control  1.000 ± 0.041  Susceptible  1.052 ± 0.060  Resilient  1.009 ± 0.070 |
| Figure S2I: Fluorescence intensity of DHE  Control (n = 6)  Susceptible (n = 6)  Resilient (n = 6) | One-way ANOVA  Stress: F (2, 15) = 25.850 | Bonferroni's post hoc test  Control vs. susceptible,  *p* < 0.001  Control vs. resilient,  *p* = 0.969 | Control  1.000 ± 0.098  Susceptible  1.916 ± 0.109  Resilient  1.100 ± 0.088 |
| Figure S2J: IBA1^+^ cells/mm^2^  Control (n = 6)  Susceptible (n = 6)  Resilient (n = 6) | One-way ANOVA  Stress: F (2, 15) = 36.410 | Bonferroni's post hoc test  Control vs. susceptible,  *p* < 0.001  Control vs. resilient,  *p* = 0.176 | Control  87.823 ± 6.053  Susceptible  160.816 ± 7.479  Resilient  104.286 ± 5.287 |
| Figure S2K: Complex I (nmol/min/mg protein)  Control (n = 5)  Susceptible (n = 5)  Resilient (n = 5) | One-way ANOVA  Stress: F (2, 12) = 20.280 | Bonferroni's post hoc test  Control vs. susceptible,  *p* < 0.001  Control vs. resilient,  *p*＞0.999 | Control  21.359 ± 1.412  Susceptible  11.709 ± 1.002  Resilient  21.962 ± 1.378 |
| Figure S2L: Complex II (nmol/min/mg protein)  Control (n = 5)  Susceptible (n = 5)  Resilient (n = 5) | One-way ANOVA  Stress: F (2, 12) = 9.480 | Bonferroni's post hoc test  Control vs. susceptible,  *p* = 0.024  Control vs. resilient,  *p* = 0.453 | Control  15.266 ± 0.944  Susceptible  11.764 ± 0.968  Resilient  16.772 ± 0.511 |
| Figure S2M: GAPDH activity (% control)  Control (n = 6)  Susceptible (n = 6)  Resilient (n = 5) | One-way ANOVA  Stress: F (2, 14) = 11.120 | Bonferroni's post hoc test  Control vs. susceptible,  *p* = 0.002  Control vs. resilient,  *p*＞0.999 | Control  1.000 ± 0.061  Susceptible  1.395 ± 0.062  Resilient  1.000 ± 0.088 |
| Figure S2O:  Relative CISD1 protein expression  0 (n = 4)  3 (n = 4)  5 (n = 4)  7 (n = 5)  10 (n = 5) | One-way ANOVA  Stress: F (4, 17) = 49.600 | Bonferroni's post hoc test  0 vs. 3, *p*＞0.999  0 vs. 5, *p* = 0.854  0 vs. 7, *p* < 0.001  0 vs. 10, *p* < 0.001 | 0  1.000 ± 0.064  3  0.997 ± 0.047  5  0.998 ± 0.055  7  1.709 ± 0.058  10  2.045 ± 0.101 |
| Figure S3B: Relative *Cisd1* mRNA expression  GFP (n = 6)  *Cisd1* (n = 6) | Unpaired *t* test  t = 7.067, df = 10, *p* < 0.001 |  | GFP  1.000 ± 0.070  *Cisd1*  2.364 ± 0.180 |
| Figure S3C: Social interaction ratio  Control-GFP (n = 8)  SSDS-GFP (n = 8)  Control-*Cisd1* (n = 8)  SSDS-*Cisd1* (n = 8) | Two-way ANOVA  Stress: F (1, 28) = 28.980  Virus: F (1, 28) = 28.510 | Bonferroni's post hoc test  Control-*Cisd1* vs. SSDS-*Cisd1*, *p* < 0.001  SSDS-GFP vs. SSDS-*Cisd1*, *p* < 0.001 | Control-GFP  1.533 ± 0.089  SSDS-GFP  1.333 ± 0.060  Control-*Cisd1*  1.330 ± 0.075  SSDS-*Cisd1*  0.788 ± 0.046 |
| Figure S3D: Time in interaction zone (s)  Control-GFP (n = 8)  SSDS-GFP (n = 8)  Control-*Cisd1* (n = 8)  SSDS-*Cisd1* (n = 8) | Two-way ANOVA  Stress: F (1, 56) = 25.090  Target: F (3, 56) = 1.692 | Bonferroni's post hoc test  *No target vs. Target*  Control-GFP, *p* < 0.001  SSDS-GFP, *p* = 0.003  Control-*Cisd1*, *p* = 0.002  SSDS-*Cisd1*, *p* = 0.009 | *No target*  Control-GFP  29.825 ± 1.765  SSDS-GFP  30.388 ± 1.682  Control-*Cisd1*  30.038 ± 1.200  SSDS-*Cisd1*  37.725 ± 1.600  *Target*  Control-GFP  45.500 ± 3.121  SSDS-GFP  39.963 ± 2.197  Control-*Cisd1*  39.713 ± 1.389  SSDS-*Cisd1*  29.338 ± 1.275 |
| Figure S3E: Time in corner zone (s)  Control-GFP (n = 8)  SSDS-GFP (n = 8)  Control-*Cisd1* (n = 8)  SSDS-*Cisd1* (n = 8) | Two-way ANOVA  Stress: F (1, 56) = 2.835  Target: F (3, 56) = 3.809 | Bonferroni's post hoc test  *No target vs. Target*  Control-GFP, *p* ＞0.999  SSDS-GFP, *p* ＞0.999  Control-*Cisd1*, *p* ＞0.999  SSDS-*Cisd1*, *p* = 0.004 | *No target*  Control-GFP  23.863 ± 1.985  SSDS-GFP  23.850 ± 2.428  Control-*Cisd1*  24.150 ± 2.048  SSDS-*Cisd1*  24.488 ± 1.839  *Target*  Control-GFP  22.200 ± 1.600  SSDS-GFP  24.800 ± 1.916  Control-*Cisd1*  24.588 ± 2.515  SSDS-*Cisd1*  35.363 ± 3.109 |
| Figure S3F: Sucrose preference  Control-GFP (n = 8)  SSDS-GFP (n = 8)  Control-*Cisd1* (n = 8)  SSDS-*Cisd1* (n = 8) | Two-way ANOVA  Stress: F (1, 28) = 8.706  Virus: F (1, 28) = 6.045 | Bonferroni's post hoc test  Control-*Cisd1* vs. SSDS-*Cisd1*, *p* = 0.007  SSDS-GFP vs. SSDS-*Cisd1*, *p* = 0.003 | Control-GFP  85.236 ± 1.673  SSDS-GFP  84.545 ± 1.905  Control-*Cisd1*  83.670 ± 1.792  SSDS-*Cisd1*  75.616 ± 1.734 |
| Figure S4B: Relative *Cisd1* mRNA expression  Control (n = 8)  SUS (n = 8) | Unpaired *t* test  t = 6.891, df = 14, *p* < 0.001 |  | Control  1.000 ± 0.065  SUS  1.733 ± 0.084 |
| Figure S4D: Relative CISD1 protein expression  Control (n = 5)  SUS (n = 6) | Unpaired *t* test  t = 5.254, df = 9, *p* = 0.001 |  | Control  1.000 ± 0.082  SUS  1.514 ± 0.058 |
| Figure S4E: Relative *Cisd1* mRNA expression  Control (n = 8)  SUS (n = 8) | Unpaired *t* test  t = 0.642, df = 14, *p* = 0.531 |  | Control  1.000 ± 0.063  SUS  1.065 ± 0.080 |
| Figure S4F: Relative *Cisd1* mRNA expression  Control (n = 8)  SUS (n = 8) | Unpaired *t* test  t = 0.300, df = 14, *p* = 0.769 |  | Control  1.000 ± 0.066  SUS  0.975 ± 0.051 |
| Figure S4G: Relative *Cisd1* mRNA expression  GFP (n = 6)  Cisd1^cKO^ (n = 6) | Unpaired *t* test  t = 10.710, df = 10, *p* < 0.001 |  | GFP  1.000 ± 0.069  Cisd1^cKO^  0.197 ± 0.029 |
| Figure S5B: Lactate level  Control (n = 5)  Susceptible (n = 5)  Resilient (n = 4) | One-way ANOVA  Stress: F (2, 11) = 4.896 | Bonferroni's post hoc test  Control vs. susceptible, *p* = 0.087  Control vs. resilient, *p* = 0.861 | Control  1.000 ± 0.099  Susceptible  0.693 ± 0.092  Resilient  1.117 ± 0.106 |
| Figure S5D: Lactate level in microglia  Control (n = 6)  Susceptible (n = 6)  Resilient (n = 4) | One-way ANOVA  Stress: F (2, 13) = 4.110 | Bonferroni's post hoc test  Control vs. susceptible, *p* = 0.028  Control vs. resilient, *p* = 0.715 | Control  1.000 ± 0.089  Susceptible  1.387 ± 0.102  Resilient  1.145 ± 0.120 |
| Figure S5E: LDH activity of microglia  Control (n = 4)  Susceptible (n = 4)  Resilient (n = 3) | One-way ANOVA  Stress: F (2, 8) = 13.330 | Bonferroni's post hoc test  Control vs. susceptible, *p* = 0.007  Control vs. resilient, *p* = 0.726 | Control  1.000 ± 0.084  Susceptible  1.478 ± 0.065  Resilient  0.877 ± 0.121 |
| Figure S5F: PDH activity of microglia  Control (n = 6)  Susceptible (n = 6)  Resilient (n = 4) | One-way ANOVA  Stress: F (2, 13) = 34.890 | Bonferroni's post hoc test  Control vs. susceptible, *p* < 0.001  Control vs. resilient, *p*＞0.999 | Control  1.000 ± 0.050  Susceptible  0.523 ± 0.036  Resilient  1.011 ± 0.063 |
| Figure S5H: Fluorescence intensity of DHE (AU)  Control-GFP (n = 6)  SUS-GFP (n = 6)  Control-Cisd1^cKO^ (n = 6)  SUS-Cisd1^cKO^ (n = 6) | Two-way ANOVA  Stress: F (1, 20) = 11.330  Virus: F (1, 20) = 47.440 | Bonferroni's post hoc test  Control-GFP vs. SUS-GFP, *p* < 0.001  SUS-GFP vs. SUS-Cisd1^cKO^, *p* < 0.001 | Control-GFP  1.000 ± 0.065  SUS-GFP  2.023 ± 0.097  Control-Cisd1^cKO^  1.080 ± 0.098  SUS-Cisd1^cKO^  1.324 ± 0.103 |
| Figure S5I: NAD^+^/NADH ratio  Control-GFP (n = 5)  SUS-GFP (n = 5)  Control-Cisd1^cKO^ (n = 6)  SUS-Cisd1^cKO^ (n = 6) | Two-way ANOVA  Stress: F (1, 18) = 4.836  Virus: F (1, 18) = 7.141 | Bonferroni's post hoc test  Control-GFP vs. SUS-GFP, *p* = 0.014  SUS-GFP vs. SUS-Cisd1^cKO^, *p* = 0.021 | Control-GFP  2.641 ± 0.245  SUS-GFP  1.653 ± 0.142  Control-Cisd1^cKO^  2.720 ± 0.256  SUS-Cisd1^cKO^  2.537 ± 0.195 |
| Figure S5J: Complex I (nmol/min/mg protein)  Control-GFP (n = 6)  SUS-GFP (n = 6)  Control-Cisd1^cKO^ (n = 6)  SUS-Cisd1^cKO^ (n = 6) | Two-way ANOVA  Stress: F (1, 20) = 23.620  Virus: F (1, 20) = 28.940 | Bonferroni's post hoc test  Control-GFP vs. SUS-GFP, *p* < 0.001  SUS-GFP vs. SUS-Cisd1^cKO^, *p* < 0.001 | Control-GFP  21.471 ± 1.514  SUS-GFP  9.484 ± 1.037  Control-Cisd1^cKO^  23.773 ± 1.674  SUS-Cisd1^cKO^  20.745 ± 1.271 |
| Figure S5K: Complex II (nmol/min/mg protein)  Control-GFP (n = 6)  SUS-GFP (n = 6)  Control-Cisd1^cKO^ (n = 6)  SUS-Cisd1^cKO^ (n = 6) | Two-way ANOVA  Stress: F (1, 20) = 7.777  Virus: F (1, 20) = 5.450 | Bonferroni's post hoc test  Control-GFP vs. SUS-GFP, *p* = 0.005  SUS-GFP vs. SUS-Cisd1^cKO^, *p* = 0.002 | Control-GFP  15.750 ± 0.764  SUS-GFP  11.743 ± 1.017  Control-Cisd1^cKO^  15.897 ± 0.715  SUS-Cisd1^cKO^  16.118 ± 0.708 |
| Figure S5L: Relative mRNA expression  Control-GFP (n = 6)  SUS-GFP (n = 6)  Control-Cisd1^cKO^ (n = 6)  SUS-Cisd1^cKO^ (n = 6) | Two-way ANOVA  Stress x Virus: F (2, 60) = 0.735 | Bonferroni's post hoc test  *Hmox1*  Control-GFP vs. SUS-GFP, *p* < 0.001  SUS-GFP vs. SUS-Cisd1^cKO^, *p* < 0.001  *Sod1*  Control-GFP vs. SUS-GFP, *p* < 0.001  SUS-GFP vs. SUS-Cisd1^cKO^, *p* = 0.002  *Sod2*  Control-GFP vs. SUS-GFP, *p* < 0.001  SUS-GFP vs. SUS-Cisd1^cKO^, *p* < 0.001 | *Hmox1*  Control-GFP  1.000 ± 0.088  SUS-GFP  0.441 ± 0.050  Control-Cisd1^cKO^  0.967 ± 0.051  SUS-Cisd1^cKO^  0.948 ± 0.065  *Sod1*  Control-GFP  1.000 ± 0.087  SUS-GFP  0.503 ± 0.042  Control-Cisd1^cKO^  0.927 ± 0.072  SUS-Cisd1^cKO^  0.822 ± 0.063  *Sod2*  Control-GFP  1.000 ± 0.055  SUS-GFP  0.533 ± 0.068  Control-Cisd1^cKO^  0.918 ± 0.051  SUS-Cisd1^cKO^  1.020 ± 0.057 |
| Figure S6B: Immobility time (s)  Vehicle (n = 8)  1 (n = 8)  2 (n = 8)  4 (n = 8)  6 (n = 8) | One-way ANOVA  Pioglitazone: F (4, 35) = 9.863 | Bonferroni's post hoc test  Vehicle vs. 1, *p*＞0.999  Vehicle vs. 2, *p*＞0.999  Vehicle vs. 4, *p* = 0.001  Vehicle vs. 6, *p* < 0.001 | Vehicle  91.038 ± 5.756  1  90.175 ± 5.915  2  88.875 ± 5.081  4  61.813 ± 4.427  6  59.350 ± 4.362 |
| Figure S6C: Immobility time (s)  Vehicle (n = 8)  1 (n = 8)  2 (n = 8)  4 (n = 8)  6 (n = 8) | One-way ANOVA  Pioglitazone: F (4, 35) = 13.990 | Bonferroni's post hoc test  Vehicle vs. 1, *p*＞0.999  Vehicle vs. 2, *p*＞0.999  Vehicle vs. 4, *p* < 0.001  Vehicle vs. 6, *p* < 0.001 | Vehicle  95.988 ± 5.107  1  96.463 ± 4.649  2  89.038 ± 3.913  4  65.000 ± 4.564  6  59.438 ± 5.268 |
| Figure S6E: Social interaction ratio  SSDS-GFP-vehicle  (n = 7)  SSDS-*Cisd1*-vehicle  (n = 7)  SSDS-GFP-pioglitazone (n = 7)  SSDS-*Cisd1*-pioglitazone (n = 7) | Two-way ANOVA  Stress: F (1, 24) = 47.710  Pioglitazone: F (1, 24) = 15.750 | Bonferroni's post hoc test  SSDS-GFP-vehicle vs. SSDS-*Cisd1*-vehicle,  *p* < 0.001  SSDS-*Cisd1*-vehicle vs. SSDS-*Cisd1*-pioglitazone,  *p* < 0.001 | SSDS-GFP-vehicle  1.421 ± 0.078  SSDS-*Cisd1*-vehicle  0.700 ± 0.103  SSDS-GFP-pioglitazone  1.696 ± 0.093  SSDS-*Cisd1*-pioglitazone  1.690 ± 0.091 |
| Figure S6F: Time in interaction zone (s)  SSDS-GFP-vehicle  (n = 7)  SSDS-*Cisd1*-vehicle  (n = 7)  SSDS-GFP-pioglitazone (n = 7)  SSDS-*Cisd1*-pioglitazone (n = 7) | Two-way ANOVA  Stress: F (1, 48) = 34.590  Target: F (3, 48) = 9.618 | Bonferroni's post hoc test  *No target vs. Target*  SSDS-GFP-vehicle,  *p* = 0.003  SSDS-*Cisd1*-vehicle,  *p* = 0.016  SSDS-GFP-pioglitazone,  *p* < 0.001  SSDS-*Cisd1*-pioglitazone  *p* < 0.001 | *No target*  SSDS-GFP-vehicle  26.057 ± 1.366  SSDS-*Cisd1*-vehicle  27.900 ± 2.161  SSDS-GFP-pioglitazone  24.786 ± 1.427  SSDS-*Cisd1*-pioglitazone  24.643 ± 1.578  *Target*  SSDS-GFP-vehicle  36.986 ± 2.852  SSDS-*Cisd1*-vehicle  18.843 ± 2.417  SSDS-GFP-pioglitazone  41.643 ± 2.378  SSDS-*Cisd1*-pioglitazone  41.143 ± 2.278 |
| Figure S6G: Time in corner zone (s)  SSDS-GFP-vehicle  (n = 7)  SSDS-*Cisd1*-vehicle  (n = 7)  SSDS-GFP-pioglitazone (n = 7)  SSDS-*Cisd1*-pioglitazone (n = 7) | Two-way ANOVA  Stress: F (1, 48) = 4.012  Target: F (3, 48) = 3.664 | Bonferroni's post hoc test  *No target vs. Target*  SSDS-GFP-vehicle,  *p* ＞0.999  SSDS-*Cisd1*-vehicle,  *p* < 0.001  SSDS-GFP-pioglitazone,  *p* ＞0.999  SSDS-*Cisd1*-pioglitazone  *p* ＞0.999 | *No target*  SSDS-GFP-vehicle  24.371 ± 2.938  SSDS-*Cisd1*-vehicle  22.243 ± 1.570  SSDS-GFP-pioglitazone  22.714 ± 1.830  SSDS-*Cisd1*-pioglitazone  23.671 ± 1.899  *Target*  SSDS-GFP-vehicle  21.729 ± 1.712  SSDS-*Cisd1*-vehicle  35.786 ± 2.744  SSDS-GFP-pioglitazone  22.743 ± 1.783  SSDS-*Cisd1*-pioglitazone  24.914 ± 2.287 |
| Figure S6H: Sucrose preference  SSDS-GFP-vehicle  (n = 7)  SSDS-*Cisd1*-vehicle  (n = 7)  SSDS-GFP-pioglitazone (n = 7)  SSDS-*Cisd1*-  pioglitazone (n = 7) | Two-way ANOVA  Stress: F (1, 24) = 18.920  Pioglitazone: F (1, 24) = 11.770 | Bonferroni's post hoc test  SSDS-GFP-vehicle vs. SSDS-*Cisd1*-vehicle,  *p* < 0.001  SSDS-*Cisd1*-vehicle vs. SSDS-*Cisd1*-pioglitazone,  *p* < 0.001 | SSDS-GFP-vehicle  82.593 ± 1.922  SSDS-*Cisd1*-vehicle  68.104 ± 1.841  SSDS-GFP-pioglitazone  81.609 ± 1.727  SSDS-*Cisd1*-pioglitazone  84.189 ± 1.408 |
| Figure S7B: Social interaction ratio  Control (n = 8)  SUS-vehicle (n = 8)  SUS-vehicle- pioglitazone (n = 8)  SUS-T0070907-pioglitazone (n = 8) | One-way ANOVA  Stress x T0070907 x pioglitazone: F (3, 28) = 24.870 | Bonferroni's post hoc test  Control vs. SUS,  *p* = 0.241  SUS vs. SSDS-pioglitazone,  *p* < 0.001  SUS-vehicle- pioglitazone vs. SUS-T0070907-pioglitazone,  *p*＞0.999 | Control  1.750 ± 0.120  SUS  0.570 ± 0.079  SSDS-vehicle-pioglitazone  1.483 ± 0.116  SSDS-T0070907-pioglitazone  1.497 ± 0.094 |
| Figure S7C: Time in interaction zone (s)  Control (n = 8)  SUS-vehicle (n = 8)  SUS-vehicle- pioglitazone (n = 8)  SUS-T0070907-pioglitazone (n = 8) | Two-way ANOVA  Stress x T0070907 x pioglitazone: F (3, 56) = 3.937 | Bonferroni's post hoc test  *No target vs. Target*  Control, *p* = 0.003  SUS, *p* = 0.012  SUS-vehicle- pioglitazone, *p* = 0.019  SUS-T0070907-pioglitazone, *p* = 0.028 | *No target*  Control  20.188 ± 2.503  SUS  26.000 ± 2.349  SSDS-vehicle-pioglitazone  22.038 ± 1.895  SSDS-T0070907-pioglitazone  22.700 ± 2.005  *Target*  Control  33.913 ± 2.907  SUS  14.025 ± 1.521  SSDS-vehicle-pioglitazone  33.438 ± 4.363  SSDS-T0070907-pioglitazone  33.563 ± 3.275 |
| Figure S7D: Time in corner zone (s)  Control (n = 8)  SUS-vehicle (n = 8)  SUS-vehicle- pioglitazone (n = 8)  SUS-T0070907-pioglitazone (n = 8) | Two-way ANOVA  Stress x T0070907 x pioglitazone: F (3, 56) = 6.308 | Bonferroni's post hoc test  *No target vs. Target*  Control, *p*＞0.999  SUS, *p* < 0.001  SUS-vehicle- pioglitazone, *p*＞0.999  SUS-T0070907-pioglitazone, *p*＞0.999 | No target  Control  25.338 ± 1.806  SUS  22.338 ± 1.778  SSDS-vehicle-  pioglitazone  24.250 ± 1.721  SSDS-T0070907-pioglitazone  22.600 ± 1.664  *Target*  Control  24.313 ± 1.506  SUS  38.925 ± 3.857  SSDS-vehicle-pioglitazone  22.963 ± 1.276  SSDS-T0070907-pioglitazone  22.225 ± 1.660 |
| Figure S7E: Sucrose preference (%)  Control (n = 8)  SUS-vehicle (n = 8)  SUS-vehicle- pioglitazone (n = 8)  SUS-T0070907-pioglitazone (n = 8) | One-way ANOVA  Stress x T0070907 x pioglitazone: F (3, 28) = 23.570 | Bonferroni's post hoc test  Control vs. SUS,  *p*＞0.999  SUS vs. SSDS-pioglitazone,  *p* < 0.001  SUS-vehicle- pioglitazone vs. SUS-T0070907-pioglitazone,  *p*＞0.999 | Control  84.154 ± 1.559  SUS  66.564 ± 2.081  SSDS-vehicle-pioglitazone  84.353 ± 1.774  SSDS-T0070907-pioglitazone  82.629 ± 1.633 |
